# Combined Effect of Shegandilong Granule and Doxycycline on Immune Responses and Protection Against Avian Infectious Bronchitis Virus in Broilers

**DOI:** 10.3389/fvets.2021.756629

**Published:** 2021-12-20

**Authors:** Haipeng Feng, Xuezhi Wang, Jingyan Zhang, Kang Zhang, Wenshu Zou, Kai Zhang, Lei Wang, Zhiting Guo, Zhengying Qiu, Guibo Wang, Ruihua Xin, Jianxi Li

**Affiliations:** Engineering and Technology Research Center of Traditional Chinese Veterinary Medicine of Gansu Province, Lanzhou Institute of Husbandry and Pharmaceutical Sciences of Chinese Academy of Agricultural Sciences, Lanzhou, China

**Keywords:** Shegandilong granule, doxycycline, avian infectious bronchitis virus, immune response, broilers

## Abstract

Infectious bronchitis (**IB**) causes significant economic losses to commercial chicken farms due to the failures of vaccine immunization or incomplete protection. In this study, we evaluated the combination effect of Shegandilong (**SGDL**) granule (a traditional Chinese veterinary medicine) and doxycycline on the prevention of IBV infection and injury in the respiratory tract in broilers. A total of 126, 7-day-old broilers were randomly divided into four groups after vaccination. Group I served as a control. Broilers in Group II were given doxycycline, and Group III was given SGDL granule through drinking water. Broilers in Group IV were given SGDL granule and doxycycline by drinking water. Broilers in all groups were challenged with IBV through intraocular and intranasal routes at day 28. Results showed that the anti-IBV antibody level was higher in group IV compared with the level in other groups. Immunohistochemistry and ELISA results showed that an increase of immunoglobulin A **(IgA**) was observed in the trachea with the maximum level observed at day 14. In addition, SGDL granule + doxycycline effectively inhibited IBV replication and stopped IBV propagation from the trachea to the lung; modulated the mRNA expressions of IL-1β, IL-6, TNF-α, and IFN-γ; and extenuated the histopathology lesions in trachea and lung. These data imply that a combination of SGDL granule and doxycycline is effective in preventing IBV infection and respiratory tract injury in broilers.

## Introduction

Avian infectious bronchitis (**IB**) is an acute and highly contagious respiratory tract disease caused by avian infectious bronchitis virus (**IBV**) belonging to the *Gamma-coronavirus* genus (*Coronaviridae* family, *Nidovirales* order), which causes high economic burden on the global poultry industry (1). IBV is characterized by tracheitis, conjunctivitis, and ciliostasis (loss of ciliary activity) in the respiratory tract and affects different varieties and age of chicken (2, 3). IBV causes huge economic problems due to reduction in weight in broilers and decrease in production or weak egg quality in layers (4, 5). In 2017, production loss was estimated at $65,000 per week for a farm with a weekly production of 1 million broilers each weighing 7 pounds according to a survey (6). Currently, there is no effective therapeutic approach for IB. Vaccination with a live bivalent vaccine of Newcastle disease virus (**NDV**) and IBV is a conventional approach for controlling IB in most chicken farms in China. Despite advances in immune protection procedures, frequent outbreaks of IB have been reported among vaccinated farms causing respiratory tract disease and production losses in densely populated poultry regions (7, 8). Therefore, it is important to develop novel prophylactic strategies to control IB outbreaks.

Recent studies had shown tremendous advances in the development of Chinese herbal medicines against virus. In China, the traditional Chinese medicines have been playing an indispensable role in fighting against COVID-19 (9). Shegandilong (**SGDL**) granule is a commercial traditional Chinese medicine formula consisting of seven herbal preparations, including *Belamcanda chinensis (L.) Redouté (radix)*., *Pheretima, Glycyrrhiza uralensis Fisch (radix)*., *Schisandra chinensis, Menispermi Rhizoma (radix), Platycodon grandiflorus (Jacq.) A. DC*, and *DarkPlumFruit*, as shown in Supplementary Table 1. A previous study reported that oral administration of SGDL granule has no toxicity effect in chicken at normal doses (10). SGDL granule has been certified and authorized as a new veterinary drug in China [*(2015) New Veterinary Drug Certificate No. 17*]. Xie et al. showed that SGDL granule effectively reduced the morbidity of IB in broilers compared with the control group in the commercial poultry farm (Gansu province, China) (11).

Doxycycline, commonly known for its anti-inflammatory and antibacterial activity, is a tetracycline derivative. Doxycycline is a widely used broad-spectrum antibiotic in clinical practice (12). Studies have shown that doxycycline has antiviral effects *in vitro*, and doxycycline was shown to inhibit replication of flavivirus (13), porcine reproductive and respiratory syndrome virus (14), vesicular stomatitis virus (15), dengue virus (16), and severe acute respiratory syndrome coronavirus in cell culture (17). Moreover, a combination of doxycycline and ribavirin showed an effective inhibition on CHIKV replication in Vero cells and attenuated its infectivity in mouse models, attenuated the swelling of spleen, and reduced the infiltration of inflammatory cells in mice (18).

Aiyegoro and Okoh reported that antibiotics combined with plant extracts were a novel method for treatment of infectious diseases (19). However, to our knowledge, few studies have reported the potential antiviral activity of doxycycline combined with traditional Chinese medicine. Therefore, according to the therapeutic effect of SGDL granule on IB and broad-spectrum antiviral activities of doxycycline reported in previous studies, we hypothesized that the combination of SGDL granule and doxycycline has antiviral activity. In this study, we explored anti-IBV activity and the mechanism of a combination of SGDL granule and doxycycline *in vivo*. The finding of this study showed that a combination of SGDL granule and doxycycline effectively protects chickens from IBV infection by improving the immune response of chickens, inhibiting IBV replication, and reducing production of inflammatory cytokines.

## Materials and Methods

### Animals

One-day-old unimmunized male white feather broiler-type commercial chickens (No. 6202955682) weighing 40 ± 4 g were purchased from a Jiuquan Wangmiao commercial hatchery (Jiuquan, Gansu province, China). Chicken forage (No. 19091121) was purchased from Beijing Keao Xieli Feed Co., Ltd. (Beijing, China). Birds were raised in negative-pressure isolation units. All birds were kept under 24-h constant lighting and allowed to take food and water *ad libitum*. Temperature in the units was controlled at 34–36°C from day 1 to 14. After day 14, temperature was gradually decreased to 26°C for the rest of the experiment period. All procedures were carried out following guidelines by the Animal Care and Use Committee, Lanzhou Institute of Husbandry and Pharmaceutical Sciences of the Chinese Academy of Agricultural Sciences [the Permission Number: **SYXK**(Gan) 2019-0002].

### Virus and Vaccine

A standard strain of avian infectious bronchitis virus (IBV-M41) was purchased from the China Institute of Veterinary Drug Control (Beijing, China). Nine-day-old SPF chicken embryos were inoculated with IBV through the allantoic cavity. Allantoic fluid was collected after three generations of blind passage, and **EID50** was 10^−5.84^/0.1 ml. Live bivalent NDV (strain LaSota) and IBV (strain H120) vaccines were purchased from Qilu Animal Health Products Co., Ltd. (Jinan, China).

### Reagents and Drugs

Doxycycline was purchased from Guangxi Baokang Biotechnology Co., Ltd. (Nanning, China). Shegandilong granule was purchased from Chengdu Zhongmu Biopharmaceutical Co., Ltd. (Chengdu, China). Chicken IBV Ab **ELISA** Kit was purchased from USA TSZ Biological Trade Co., Ltd. (Shanghai, China). The Chicken IgA ELISA kit was purchased from Beijing Solarbio Science & Technology Co., Ltd. (Beijing, China). The mouse monoclonal antibody against chicken IgA was purchased from Southern Biotechnology Inc. (Birmingham, AL, USA). The anti-mouse/rabbit universal immunochemistry detection kit was purchased from Wuhan Sanying Biotechnology Co., Ltd. (Wuhan, China).

### Experimental Design

A total of 126 1-day-old chicks were randomly divided into four groups. At day 7, all groups received intraocular and intranasal immunization with a live bivalent vaccine of NDV and IBV in saline solution (50 μl). Group I was set as a control. At days 8–12, days 15–19, and days 22–26, chicks in group II were given doxycycline (0.2 g/kg BW per chick) with drinking water, whereas chicks in group III were given SGDL granule (1.0 g/kg BW per chick) with drinking water. Further, chicks in group IV were given SGDL granule in the morning and doxycycline in the afternoon through drinking water, once per day. At day 28, broilers in all groups were infected with IBV-M41 (10^4^ EID50/0.3 ml per bird) through an ocular–nasal route, and clinical symptoms were recorded daily.

### Collection of Samples

Serum samples (six birds/group) were collected at days 7, 14, 21, and 28 before sacrificing birds. Six chickens from each group were humanely euthanized for collection of ~2 cm of the upper trachea. The trachea samples were frozen instantly in liquid nitrogen for immunohistochemistry (**IHC**). The other 5 cm of the trachea was used to carry out tracheal washes, and collected samples were centrifuged at 3,000 rpm for 7 min. The supernatant from tracheal washes was stored at −20°C until further use. After days 1, 3, 5, and 7 post-infection (dpi), three birds from each group were humanely necropsied, and lung and trachea samples were collected. Partial tracheas were placed in RNAlater solution and stored at −80°C and later processed for determination of viral RNA loads and cytokines. The other tracheal portions were fixed in neutral formalin solution for histopathology observation. Lung tissue was harvested for histopathology, determination of viral RNA loads, and cytokine examination.

### ELISA for Anti-IBV and IgA Antibody Level Detection

IBV-specific antibody levels in serum and IgA level in tracheal washes were detected using a commercial chicken IBV Ab ELISA kit (CG6845, USA TSZ Biological Trade Co., Ltd.) and IgA ELISA kit (SEKCN-0018-96T, Beijing Solarbio Science & Technology Co., Ltd.) according to the manufacturers' instructions. The detection range of the IBV Ab ELISA kit is from 2.5 to 180 ng/l, and the IgA ELISA kit is from 94 to 6,000 pg/ml with a sensitivity of 18 pg/ml. Samples were added to antigen-coated 96-well microplates and incubated for 1 h at room temperature. The antibody was added to each well and incubated for 1 h at room temperature. Further, streptavidin–**HRP** and substrate solution were added to the wells. Then, the reaction was stopped and the optical density was determined at 450 nm. The corresponding concentration was calculated using a standard curve.

### Immunohistochemistry Detection of IgA^+^ Cells in Trachea Mucosa

Optimal cutting temperature (**OCT**) compound-embedded trachea tissues were frozen for 15 s in liquid nitrogen. Frozen tissues were cut into 4-μm thickness and dried for 30 min at room temperature. Tissue sections were fixed in pre-cooled acetone for 10 min at 4°C, dried for 30 min at 37°C, and washed three times with Tris-buffered saline (**TBS**) for 5 min. Further, 3% hydrogen peroxide was added to tissue sections and incubated for 15 min at room temperature. Then, tissue sections were washed three times with TBS for 5 min and blocked with 10% normal goat serum for 30 min at 37°C. After blocking with goat serum, tissue sections were incubated overnight at 4°C in a humidified chamber to detect IgA^+^ cells using the mouse monoclonal antibody (8330-01, Southern Biotechnology Inc.) against chicken IgA at 1:200. After 1 h of room temperature recovery, tissue sections were washed three times with Tris-Buffered Saline-Tween (**TBST**) for 2 min. Further, tissue sections were incubated with 37°C of secondary antibody for 30 min and washed three times with TBS for 2 min. Tissue sections were incubated with diaminobenzidine (**DAB**) for 5 min, washed with distilled water, counterstained with hematoxylin for 3 min, dehydrated, and observed under a microscope.

### Histopathological Analysis

At days 1, 3, 5, and 7 post-infection, lung and tracheal tissues were collected and fixed in 10% formalin. Tissues were embedded in paraffin wax (60°C) and cut into 5-μm-thick sections, followed by dewaxing, dehydration by gradient alcohol, and then H&E dyeing. Sections were dehydrated using gradient alcohol, dewaxed, sealed using a cover slide, and observed under a microscope.

### Detection of Virus RNA Loads and Cytokines Using RT-qPCR

Total RNA was extracted from tracheas and lungs at days 1, 3, 5, and 7 post-infection using TRIzol Reagent (Invitrogen, Carlsbad, CA, USA) according to the manufacturer's instructions. RNA purity and concentration were measured using Eppendorf spectrophotometer. cDNA was synthesized using the PrimeScript RT Reagent Kit with gDNA Eraser (Takara, Dalian, China) according to the manufacturer's instructions. Then, cDNA was stored at −20°C until further use.

Absolute quantitative RT-PCR was used to quantify viral RNA using SYBR^®^ Premix Ex Taq™ II (Takara, Dalian, China). The concentration of the constructed plasmid was converted into the RNA copies according to the formula: copies/mL = (6.02 × 10^23^) × (plasmid concentration × 10^−9^)/(DNA length × 660) = 1.32 × 10^12^. The cycle threshold (**CT**) value was converted to copies of viral RNA by generating a standard curve of six 10-fold dilutions of plasmid. mRNA expression levels of cytokines (IL-6, IL-1β, TNF-α, IFN-γ) were determined by relative quantitative RT-PCR, using SYBR^®^ Premix Ex Taq™ II (Takara, Dalian, China). mRNA expression levels of each sample were standardized by using the CT value of β-actin. The relative expression of genes was calculated using the 2 ^−Δ*ΔCt*^ method, ΔΔCt = [CT (tested sample, target gene) – CT (tested sample, housekeeping gene)] – [CT (control sample, target gene) – CT (control sample, housekeeping gene)]. Sequences of primers used in this study are listed in Supplementary Table 2.

### Statistical Analysis

Student's *T*-test (two groups) or one-way analysis of variance (ANOVA) test followed by Tukey's test (more than two groups) were used to analyze differences between and within groups using Prism 7 software (GraphPad, La Jolla, CA, USA). *p* < 0.05 was considered statistically significant.

## Results

### SGDL Granule Combined With Doxycycline Increases the Anti-IBV Antibody Level

**Figure 1:**
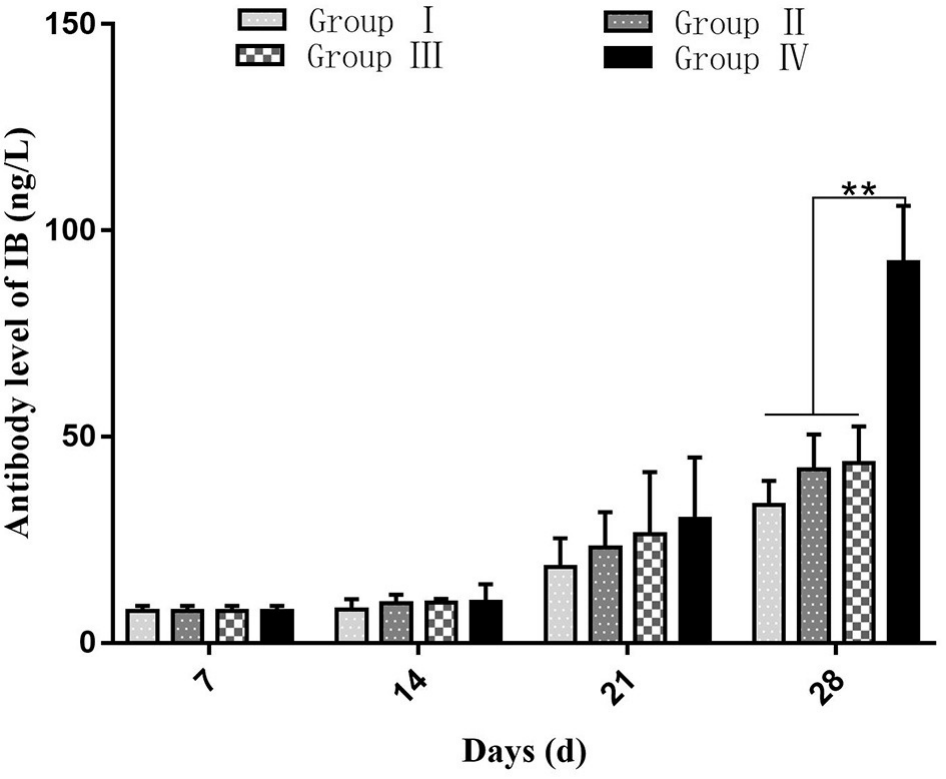
Effect of SGDL granule or doxycycline and both on the antibody response to vaccination against IBV. Group I served as a control. Broilers in Group II were given doxycycline whereas Group III was given SGDL granule through drinking water. Broilers in Group IV were given SGDL granule and doxycycline in drinking water. Extremely significant (*p* < 0.01) different values are shown with **, and no signal was not significantly (*p* > 0.05) different between the groups at those time points. SGDL, Shegandilong.

At day 7, the average value of maternally derived anti-IBV antibody titer was 4.027 ± 2.21 ng/l in all groups. The antibody level in all groups showed a significant increase with the highest level observed at day 28, and the antibody level in group IV was higher (*p* < 0.01) than other three groups. At day 14 and 21, the antibody level showed no difference (*p* > 0.05) among the four groups, as shown in Figure 1.

### Effects of SGDL Granule Combined With Doxycycline on Trachea Mucosa Immunity

**Figure 2:**
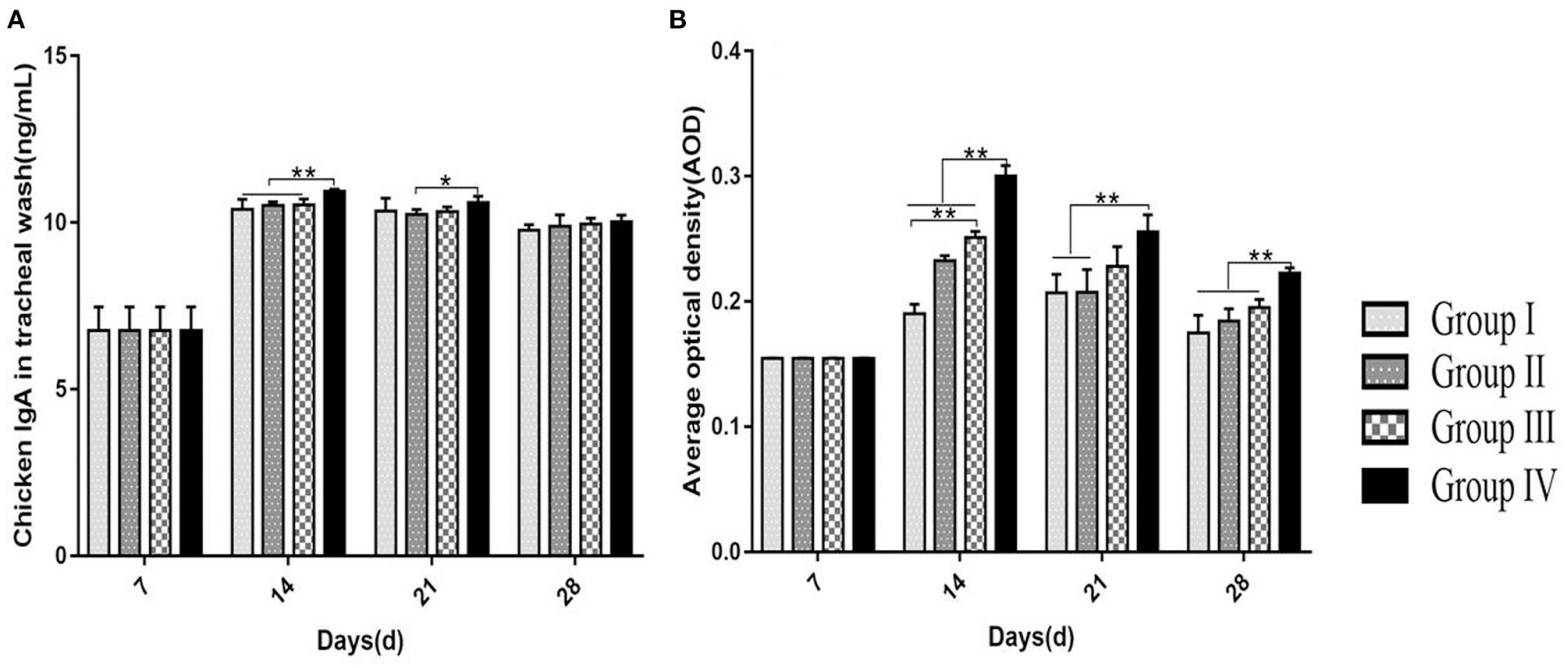
Effect of doxycycline or SGDL granule and both on IgA concentration in tracheal washing using ELISA kit **(A)** and AOD using ImageJ analysis of the expression of IgA^+^ cells in the tracheal mucosa of chicken **(B)**. Group I served as a control. Broilers in Group II were given doxycycline whereas Group III was given SGDL granule through drinking water. Broilers in Group IV were given SGDL granule and doxycycline in drinking water. Extremely significant (*p* < 0.01) different values are shown with **. Significantly (*p* < 0.05) different values are shown with *, and no signal was not significantly (*p* > 0.05) different between the groups at those time points. SGDL, Shegandilong.

The concentration of IgA in the tracheal washing increased gradually after immunization with the highest level which appeared at day 14. Group IV showed a high IgA level compared with the IgA levels in groups I, II, and III (*p* < 0.01). The IgA level showed a slight decline in all groups from day 21 to 28. At day 21, the IgA level in group IV was higher (*p* < 0.05) compared with the IgA level in group II. At day 28, the IgA level showed no difference (*p* > 0.05) among all groups (Figure 2A).

**Figure 3:**
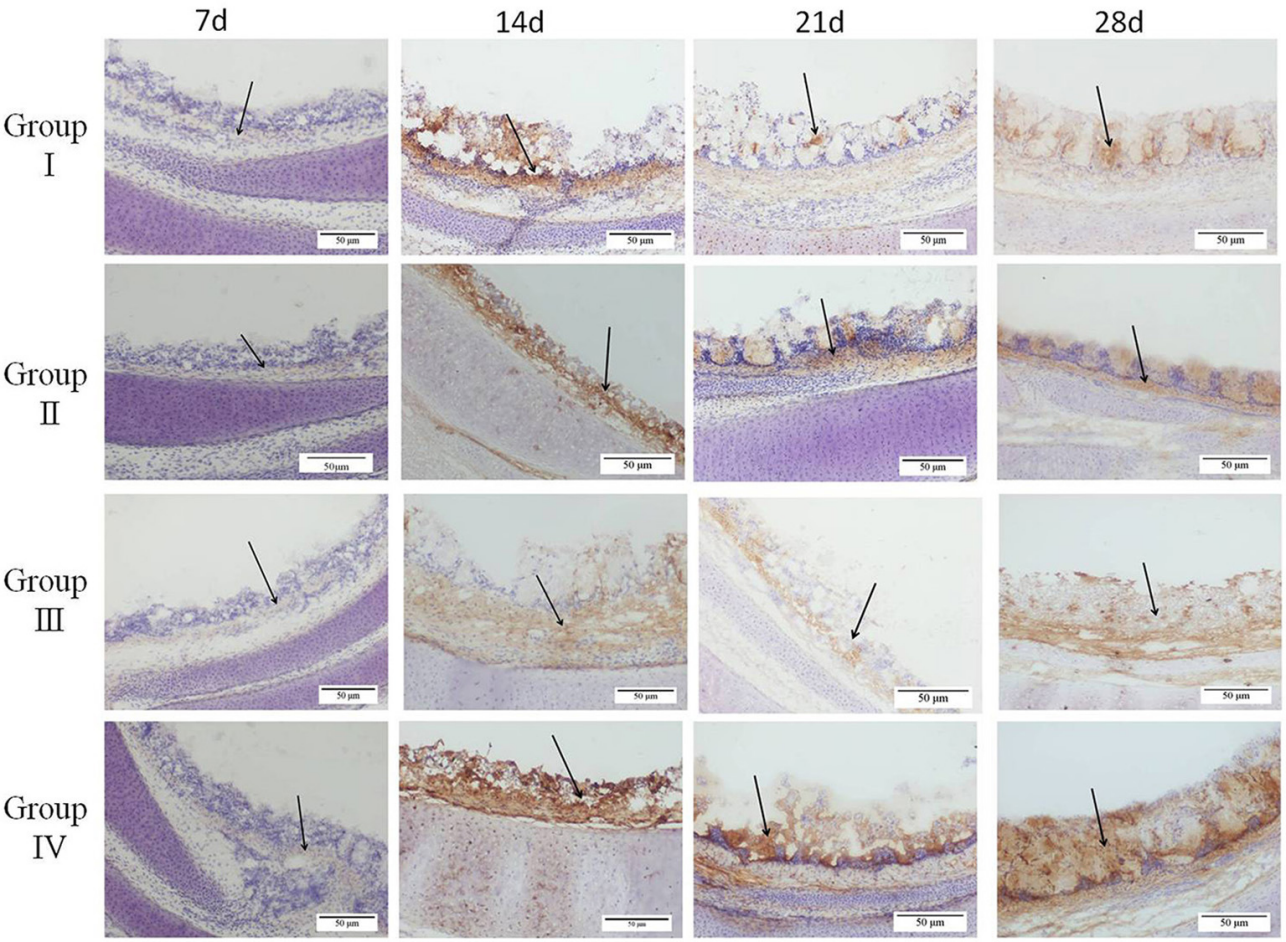
Effect of doxycycline or SGDL granule and both on IgA^+^ cells in the tracheal mucosa of chickens by immunohistochemical detection. Group I served as a control. Broilers in Group II were given doxycycline whereas Group III was given SGDL granule through drinking water. Broilers in Group IV were given SGDL granule and doxycycline in drinking water. Magnification, ×200. Black arrows indicate positive cells. SGDL, Shegandilong.

The level of IgA^+^ cells on the mucosal surface of the trachea was determined by IHC. At day 7, IgA^+^ cells on the mucosal surface of the trachea were few and mainly distributed in the mucosa lamina propria of the trachea. From day 14 to 28, most of the IgA^+^ cells were distributed in the lamina propria and upper layer of the trachea mucosa with a few IgA^+^ cells in the submucosa (Figure 3). The number of IgA^+^ cells increased gradually and reached a maximum level at day 14 in groups II, III, and IV. The number of IgA^+^ cells in group IV was higher (*p* < 0.01) compared with the number in other groups at day 14. In addition, group III showed higher (*p* < 0.01) level of IgA^+^ cells at day 14 compared with the level in group I. The number of IgA^+^ cells in groups II, III, and IV showed a slight decrease from day 21 to 28. The number of IgA^+^ cells in group I was highest at day 21, and being decreased till day 28. At day 21 and 28, the number of IgA^+^ cells was higher (*p* < 0.01) in group IV compared with other groups (Figure 2B).

### Clinical Signs and Pathological Damage Induced by IBV Infection

**Figure 4:**
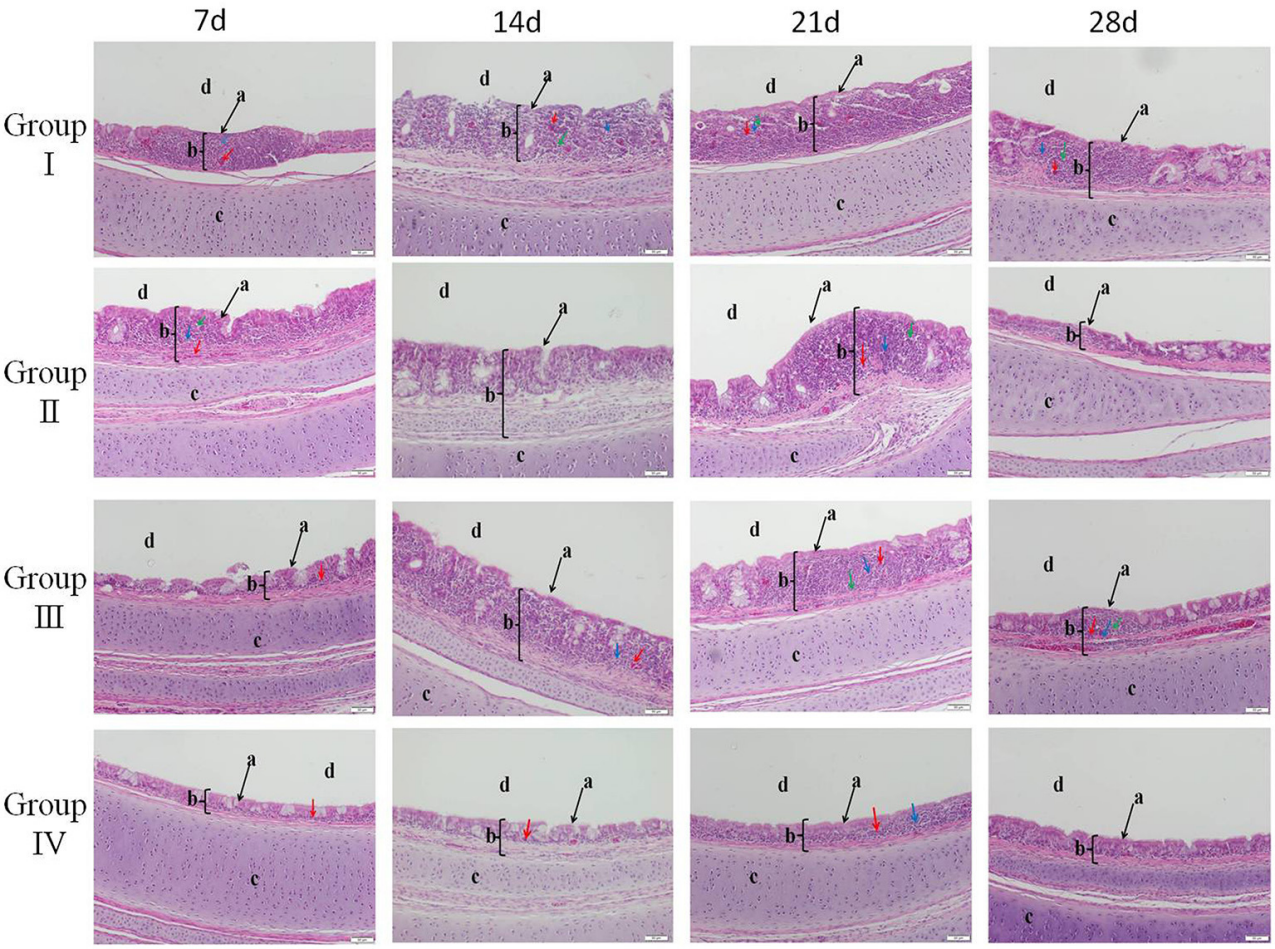
**HE** staining to delineate the relieving effect of SGDL granule combined with doxycycline on IBV-induced trachea histopathogic changes (magnification, ×200). Group I served as a control. Broilers in Group II were given doxycycline whereas Group III was given SGDL granule through drinking water. Broilers in Group IV were given SGDL granule and doxycycline in drinking water. It was intraocularly and intranasally challenged by IBV at day 28. a indicates cilia; b indicates trachea mucosa; c indicates trachea cartilage; and d indicates trachea lumen. Red arrow: congestion; blue arrow: lymphoid cell; green arrow: mononuclear cell. SGDL, Shegandilong; HE, hematoxylin–eosin.

Clinical symptoms were recorded at days 1, 3, 5, and 7 post-infection of IBV. There was no death case in all groups. Histopathological results showed that the trachea of chicks in group IV were normal with no pathological changes at day 1 post-infection. However, severe lesions were observed in the trachea of group I chicks. Histological changes mainly included tracheal cilia shedding, and high levels of inflammatory cells were infiltrated in the submucosa. The infiltration of monocytes and lymphocytes was manifested mainly in the submucosa. Tracheal lesions in groups II and III were fewer compared with lesions in group I. At day 3 post-infection, the most significant pathological changes occurred in group I. These changes included loss of cilia and epithelial cells, congestion, degeneration of glands, and infiltration of monocytes and lymphocytes in the mucosa. Pathological changes were mild in group IV and mainly included slight cilia exfoliation, degeneration of glands, and congestion. At day 5 post-infection, group II showed the most severe pathological changes, which included loss of cilia, degeneration of glands, and infiltration of monocytes and lymphocytes in the mucosa. At day 7 post-infection, group III showed significant monocyte and lymphocyte infiltration, hemorrhage, and glandular hyperplasia, as shown in Figure 4.

**Figure 5:**
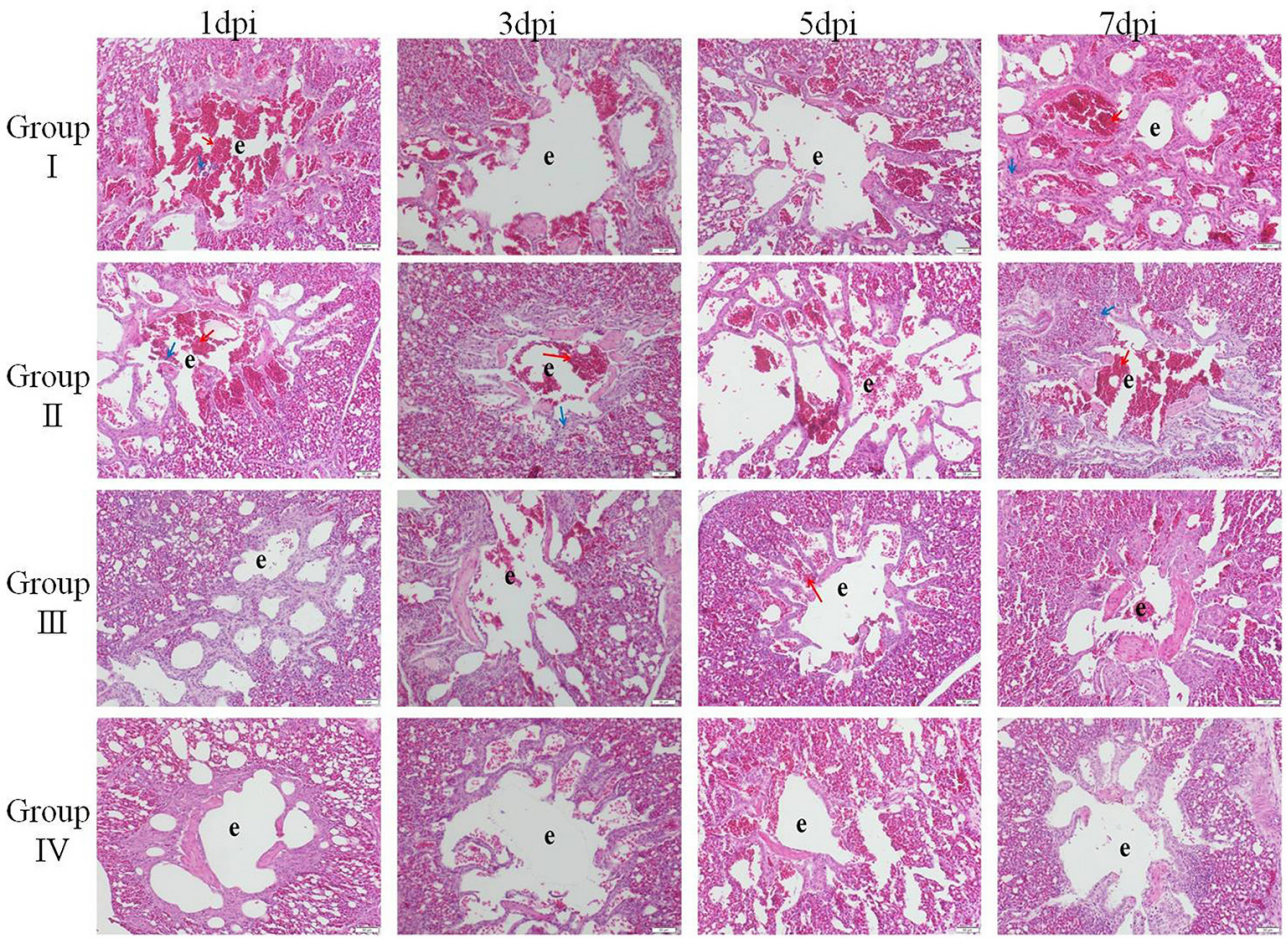
**HE** staining to delineate the relieve effect of SGDL granule combined with doxycycline on IBV-induced lung histopathogic changes (magnification, ×200). Group I served as a control. Broilers in Group II were given doxycycline whereas Group III was given SGDL granule through drinking water. Broilers in Group IV were given SGDL granule and doxycycline in drinking water. It was intraocularly and intranasally challenged by IBV at day 28. e indicates parabroncheal lumen. Red arrow: congestion; blue arrow: lymphoid cell. SGDL, Shegandilong; HE, hematoxylin–eosin.

Lungs of chicks in group I showed severe hemorrhages in the parabroncheal lumen and vascular lumen at day 1 post-infection. In addition, chicks in this group showed small amounts of monocyte and lymphocyte infiltration in the alveolar space at day 5 post-infection. At day 7 post-infection, chicks in group II showed hemorrhages in parabroncheal lumen, and chicks in group III showed slight monocytes and lymphocytes infiltration. Interestingly, chicks in group IV did not show significant pathological changes, as shown in Figure 5.

### Viral Loads in the Lungs and Trachea of Infected Birds

**Figure 6:**
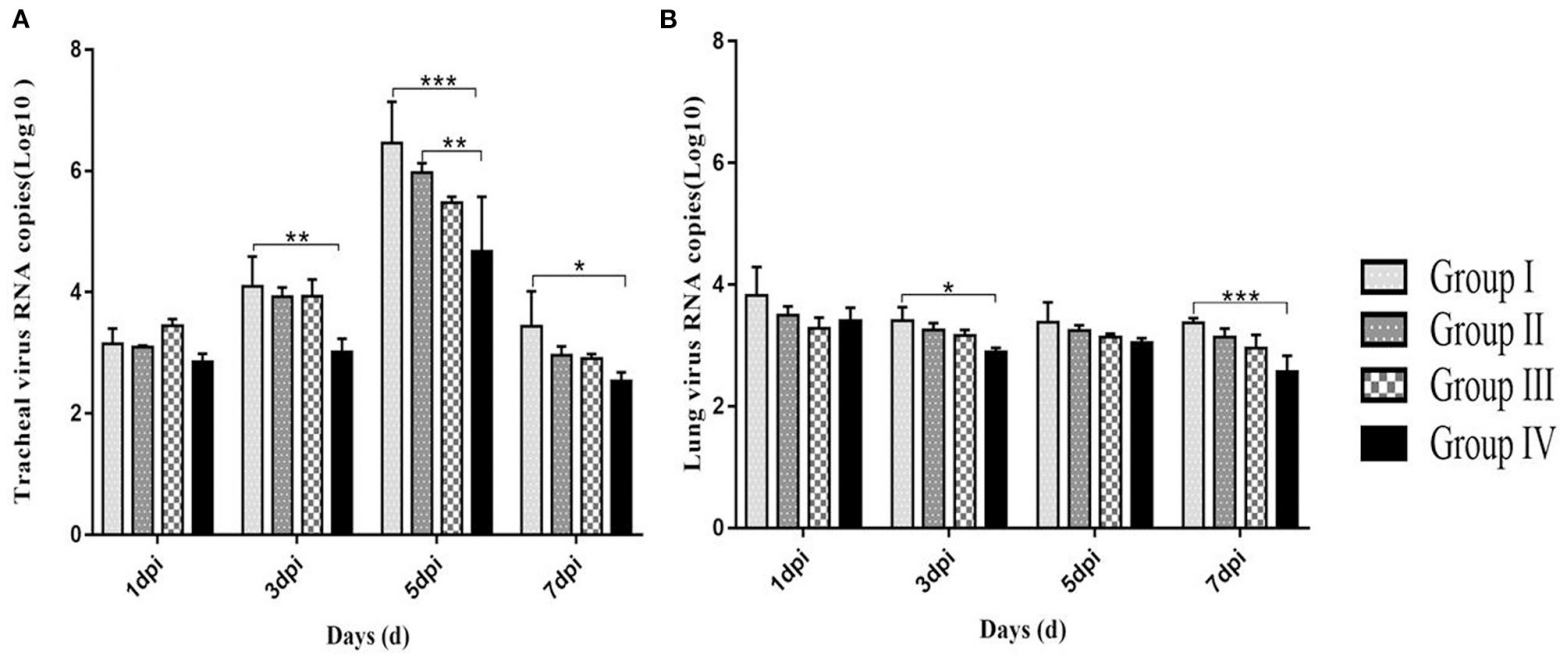
Log10 of IBV RNA copies from tracheas **(A)** and lungs **(B)** in 1, 3, 5, and 7 dpi. Group I served as a control. Broilers in Group II were given doxycycline whereas Group III was given SGDL granule through drinking water. Broilers in Group IV were given SGDL granule and doxycycline in drinking water. It was intraocularly and intranasally challenged by IBV at day 28. Extremely significant (*p* < 0.005) different values are shown with ***, extremely significant (*p* < 0.01) different values are shown with **, significantly (*p* < 0.05) different values are shown with *, and no signal was not significantly (*p* > 0.05) different between the groups at those time points. SGDL, Shegandilong.

Viral RNA loads in the trachea showed a gradual increase in all groups at days 1, 3, and 5 post-infection. Viral loads in tracheas reached the highest at day 5 post-infection. Notably, tracheal viral loads were lower (*p* < 0.01) in group IV compared with viral loads in groups I and II. Tracheal viral RNA loads at day 7 post-infection were equal to viral loads at day 1 post-infection. At day 7 post-infection, viral loads in group IV were lower (*p* < 0.05) compared with viral loads in group I (Figure 6A). Viral RNA loads in lungs showed no significant changes from day 1 to 7 post-infection. Further, viral loads in lungs from group IV were lower compared with viral loads of lungs from group I (*p* < 0.05) at day 3 and 7 (*p* < 0.01) post-infection (Figure 6B). These findings showed that virus RNA copies were lower in lungs compared with viral RNA loads in the trachea.

### Relative Expression of Cytokines in Infected Birds

**Figure 7:**
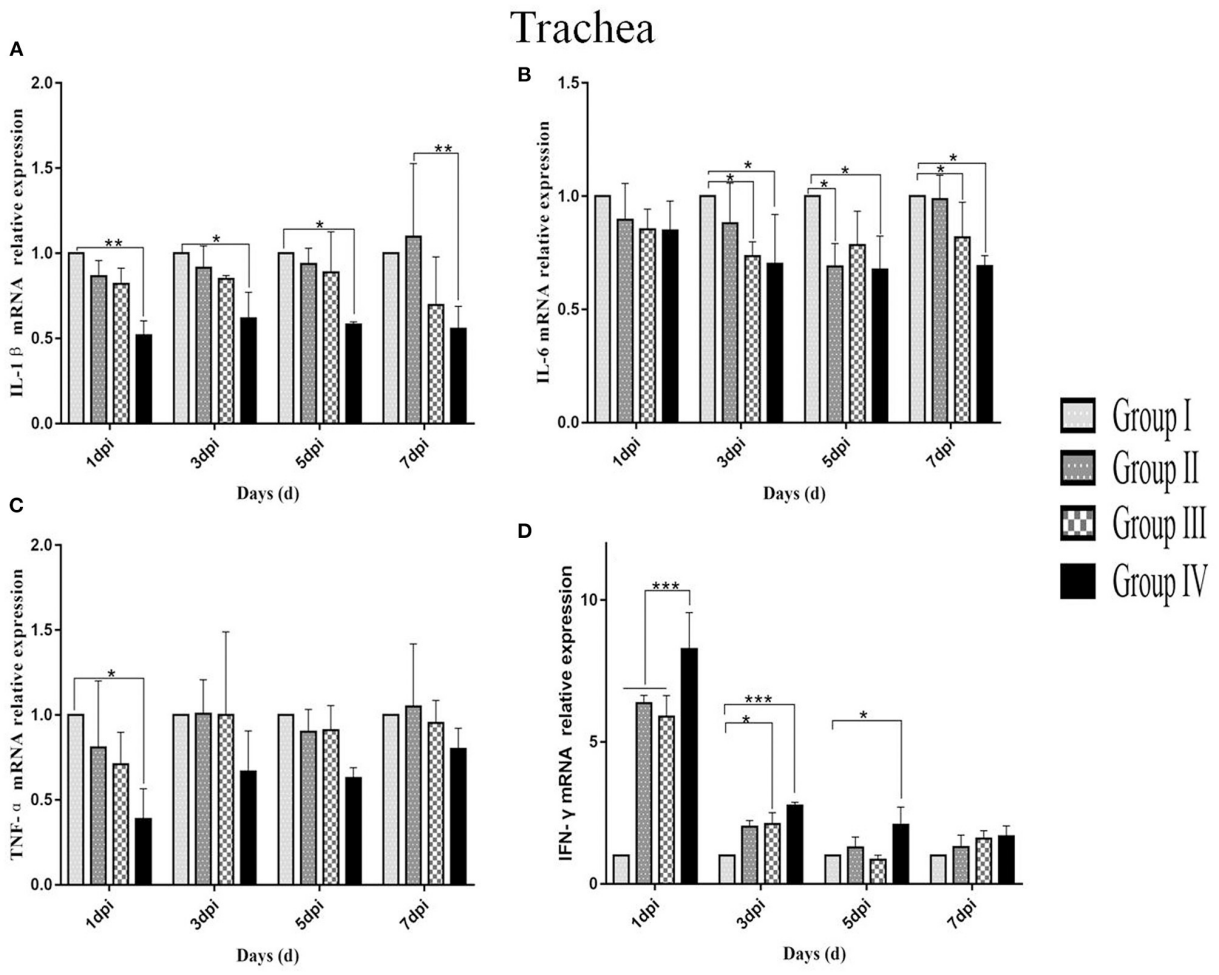
Relative mRNA expressions of IL-1β, IL-6, TNF-α, and IFN-γ in tracheas at 1, 3, 5, and 7 dpi. **(A**–**D)** Represent IL-1β, IL-6, TNF-α, and IFN-γ mRNA relative expression in trachea, respectively. Group I served as a control. Broilers in Group II were given doxycycline whereas Group III was given SGDL granule through drinking water. Broilers in Group IV were given SGDL granule and doxycycline in drinking water. It was intraocularly and intranasally challenged by IBV at day 28. Extremely significant (*p* < 0.005) different values are shown with ***, extremely significant (*p* < 0.01) different values are shown with **, significantly (*p* < 0.05) different values are shown with *, and no signal was not significantly (*p* > 0.05) different between the groups at those time points. SGDL, Shegandilong.

**Figure 8:**
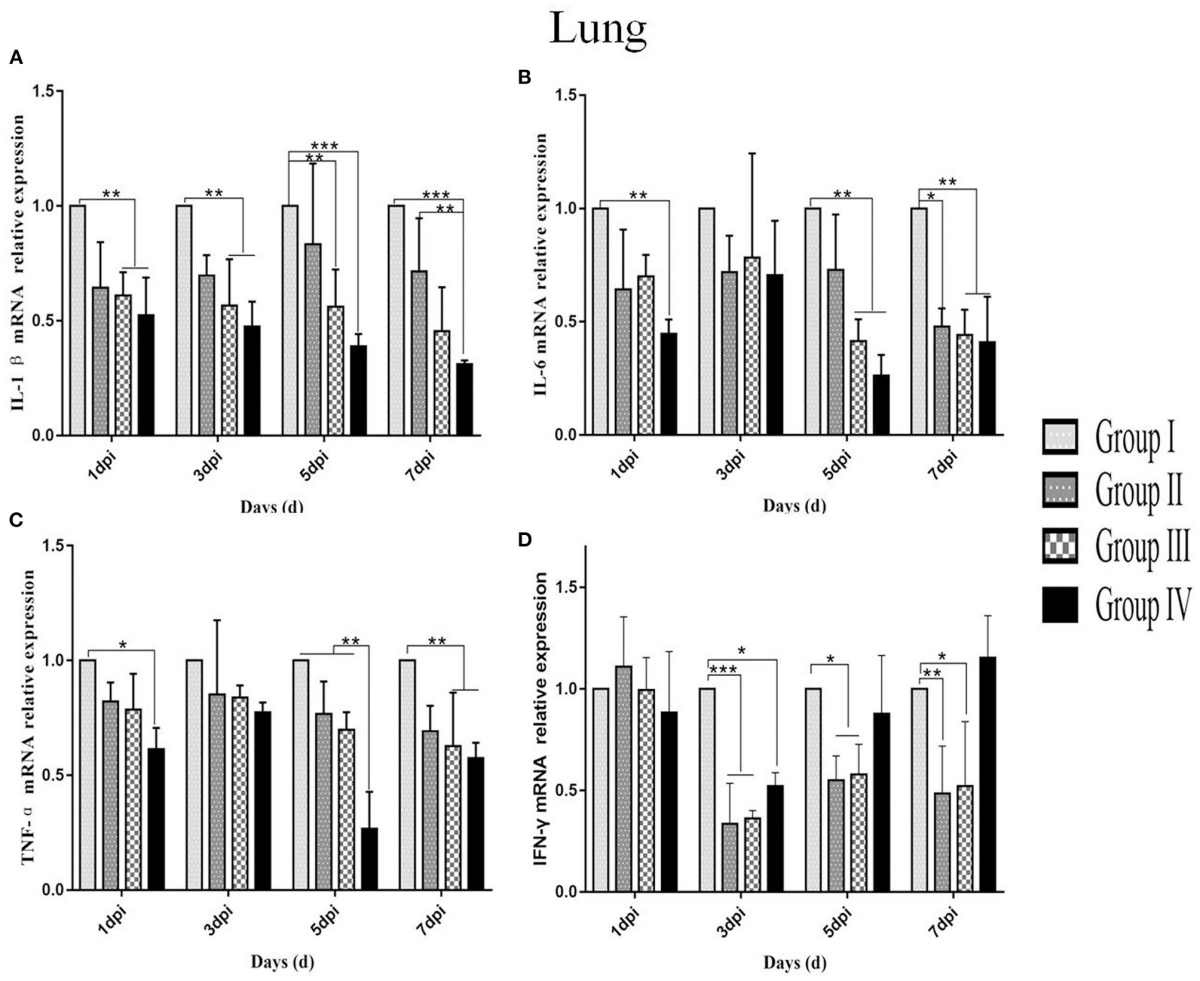
Relative mRNA expressions of IL-1β, IL-6, TNF-α, and IFN-γ in the lungs at 1, 3, 5, and 7 dpi. **(A**–**D)** Represent IL-1β, IL-6, TNF-α, and IFN-γ mRNA relative expressions in the lung, respectively. Group I served as a control. Broilers in Group II were given doxycycline whereas those in Group III were given SGDL granule through drinking water. Broilers in Group IV were given SGDL granule and doxycycline in drinking water. It was intraocularly and intranasally challenged by IBV at day 28. Extremely significant (*p* < 0.005) different values are shown with ***, extremely significant (*p* < 0.01) different values are shown with **, significantly (*p* < 0.05) different values are shown with *, and no signal was not significantly (*p* > 0.05) different between the groups at those time points. SGDL, Shegandilong.

IL-1β mRNA expression levels in the lung and trachea of group IV were lower compared with other three groups from day 1 to 7 post-infection. In trachea, the IL-1β mRNA expression level in group IV showed no significant changes from day 1 to 5 post-infection. However, the IL-1β mRNA expression in trachea was downregulated in group IV compared with IL-1β mRNA expression in group I from day 1 to 5 post-infection (*p* < 0.05) and compared with level in group II (*p* < 0.01) at day 7 post-infection (Figure 7A). In the lung, the IL-1β mRNA expression in group IV decreased gradually from day 1 to 7 post-infection. The IL-1β mRNA expression level in group IV and group III between day 1 and 3 post-infection were lower (*p* < 0.01) compared with the expression level of IL-1β mRNA in group I. Group IV showed a lower IL-1β mRNA expression level compared with group I (*p* < 0.001) and group II (*p* < 0.01) at day 5–7 post-infection (Figure 8A).

The IL-6 mRNA expression level in group IV was lower in the trachea and lung compared with other groups at day 1–7 post-infection. In the trachea, the IL-6 mRNA expression in group IV was significantly lower (*p* < 0.05) compared with IL-6 mRNA expression in group I at day 3–7 post-infection. Further, the IL-6 mRNA expression in the trachea of group III was lower (*p* < 0.05) compared with levels in group I at day 3 and 7 post-infection. The IL-6 mRNA expression in group II was lower (*p* < 0.05) compared with levels in group I at day 5 post-infection (Figure 7B). The IL-6 mRNA expression in the lungs of group IV was lower (*p* < 0.05) compared with levels in group I at day 1 post-infection. Furthermore, the IL-6 mRNA expression level in the lungs of groups IV and III were lower (*p* < 0.05) compared with group I at days 5 and 7 post-infection. In addition, the IL-6 mRNA expression level in group II was lower (*p* < 0.05) compared with group I at day 7 post-infection (Figure 8B).

The TNF-α mRNA expression in the trachea and lung of group IV was lower compared with expression levels in groups I, II, and III from day 1 to 7 post-infection. In the trachea, TNF-α mRNA expression in group IV was significantly different (*p* < 0.05) compared with the expression level in group I at day 1 post-infection, as shown in Figure 7C. In the lung, the TNF-α mRNA expression in group IV was lower (*p* < 0.05) compared with expression level in group I at day 1 post-infection. Moreover, the TNF-α mRNA expression level was lower (*p* < 0.01) in the lungs of group IV compared with the level in groups I, II, and III at day 5 post-infection. The TNF-α mRNA expression levels in groups IV and III were lower (*p* < 0.01) compared with the expression levels in group I at day 7 post-infection, as shown in Figure 8C.

The IFN-γ mRNA expression in the trachea of group IV was higher compared with the expression levels in groups I, II, and III from day 1 to 7 post-infection. The IFN-γ mRNA expression in group IV was different (*p* < 0.01) compared with the expression levels in groups I, II, and III at day 1 post-infection. At day 3 post-infection, the IFN-γ mRNA expressions in group IV (*p* < 0.01) and group III (*p* < 0.05) were different compared with the expression level in group I. The IFN-γ mRNA expression in group IV was different (*p* < 0.05) compared with the expression level in group I at day 5 post-infection. There is no difference (*p* > 0.05) in all groups at day 7 post-infection (Figure 7D). In the lung, the IFN-γ mRNA expressions in the other three groups were lower than that in group I and the IFN-γ mRNA expression in group IV was higher than those of groups II and III at days 3, 5, and 7 post-infection. The IFN-γ mRNA expressions in group IV (*p* < 0.05) and groups III and II (*p* < 0.01) were different compared with the expression level in group I at day 3 post-infection. The IFN-γ mRNA expressions in groups II and III (*p* < 0.05) were different compared with the expression level in group I at day 5 post-infection. The IFN-γ mRNA expressions in groups II (*p* < 0.01) and III (*p* < 0.05) were significantly different compared with the expression level in group I at day 7 post-infection. There is no difference (*p* > 0.05) in all groups at day 1 post-infection (Figure 8D).

## Discussion

The findings of this study showed that a combination of SGDL granule and doxycycline induced strong humoral and mucosal immunity in chicken. In this study, we determined the inhibitory effect of doxycycline combined with SGDL granule against IBV-M41 infection on 28-day-old broiler chicken. Results showed a significant inhibitory effect against IBV after administration with SGDL granule individually or in combination with doxycycline. Further, simultaneous administration of the two drugs reduced the expression level of cytokines (IL-1β, IL-6, TNF-α, and IFN-γ) in the trachea and lung. Therefore, SGDL granule and doxycycline alleviate pathological damage of the trachea and lung caused by IBV infection. Interestingly, a combination of SGDL granule and doxycycline was more effective compared with doxycycline alone.

Previous studies reported that antibody level has a low correlation with protection against IBV infection (20, 21). However, a study reported that humoral immunity plays a key role in virus elimination and lesion recovery (22). A combination of SGDL granule and doxycycline boosted the IBV antibody level in serum after immunization with live IBV vaccine. Similar findings have been reported by Ma et al., which demonstrated that the combination of ginseng stem-leaf saponins and selenium improved the antibody level after immunization with live IBV vaccine (23). In addition, antibody levels in the doxycycline-treated group were higher than that of the control group. It is consistent with the research results of Pomorska-Mól that doxycycline improved the antibody level significantly after administration of inactivated porcine erysipelas vaccine (24).

Immunoglobulin A (IgA) plays an important role in immune responses in respiratory tract mucosal surfaces. Ganapathy et al. reported that higher levels of IgA in tears and tracheal washes were positively correlated with reduction in tracheal histopathological damage and reduced virus invasion (25). Therefore, the high level of IgA prevents pathogen entry at mucosal surfaces and neutralizes virus in infected epithelial cells. Results from our study showed a gradual increase in IgA concentration in tracheal washes with a maximum level observed at day 14 of age. Notably, the IgA level in group IV was significantly higher compared with IgA levels in other groups. Similar findings were reported in a previous study that explored IgA production in tracheal washing following immunization using two commercially live IBV vaccines (3). Although the IgA level in tracheal washes showed a slight decrease at day 21, the IgA levels in three treatment groups were significantly higher compared with the control group. In addition, immunohistochemical results showed that the level of IgA^+^ cells was maximum at day 14 of age, and the level of IgA^+^ cells was significantly higher in group IV compared with the level of IgA^+^ cells in other groups. Furthermore, kinetic change in the IgA concentration determined by ELISA was consistent with change of IgA^+^ cells shown in IHC results. A similar pattern of IgA expression was reported in a previous study that showed that the presence of the IgA antibody on the mucosal surface is vital for IBV clearance (26).

Studies confirmed that the process of tissue damage in chickens following IBV infection was significantly correlated with viral RNA loads (27–29). Cavanagh et al. demonstrated that IBV was initially replicated at the infection site (tracheal epithelial cell) and then gradually spread to other organs (21). In this study, the trachea showed higher viral RNA loads compared with the lung. Further, viral RNA loads were significantly lower in group IV compared with viral loads in group I from day 1 to 7 post-infection. In addition, histopathology results of the trachea and lung showed moderate damage caused by the IBV-M41 in groups I, II, and III compared with the damage in group IV. Histological changes of tracheal tissue showed deciliation, hyperplasia of the glandular epithelium with infiltration of monocytes and lymphocytes in submucosa, and sloughing of epithelial cells. Histological analysis of the lung showed congestion of blood vessels and slight infiltration of inflammatory cells with hemorrhage in the parabroncheal lumen. As a result of IBV infection, the production of high levels of inflammatory cytokines such as TNF-α, IL-6, and IL-1β contributes to pathological damage of the trachea and lungs (30, 31). In the present study, the mRNA expression of inflammatory cytokines (IL-1β, IL-6, TNF-α) in the trachea and lung of group IV was lower compared with the expression levels of other groups from day 1 to 7 post-infection. The IFN-γ mRNA expression in the trachea of group IV was higher compared with the expression level of other groups from day 1 to 7 post-infection. However, the IFN-γ mRNA expression in the lung of other groups was lower compared with the expression level of group I from day 3 to 7 post-infection. This might be related to the viral loads in the trachea and lung; as the viral load in the lung was gradually decreased, so did the expression of IFN-γ. These findings implied that administration of SGDL granule individually or in combination with doxycycline inhibited the expression of inflammatory cytokines and increased the mRNA expression of IFN-γ. Interestingly, the combination of SGDL granule and doxycycline showed a higher inhibitory effect than SGDL granule or doxycycline. Trachea and lung tissue histopathological results showed that chicken in group IV had higher protection from IBV infection compared with chicken in other groups.

The anti-IBV antibody level and IgA level were higher, and pathological lesion was less in the trachea and lung of group IV than those of other three groups after IBV infection, which implied that SGDL granule combined with doxycycline improved the humoral and mucosal immunity of chicken. Therefore, the SGDL granule combined with doxycycline prevented IBV infection. These findings are consistent with previous studies that IgA concentration and antibody level play an important role in viral clearance and repair of tissue lesions following respiratory virus infection (27, 32). Therefore, it was confirmed that SGDL granule combined with doxycycline could prevent IBV infection through improving the immune function of broilers.

Furthermore, our results showed that viral RNA loads and mRNA expression levels of IL-1β, IL-6, and TNF-α following IBV infection in group IV were significantly lower compared with those in group I. These data showed that the combination of two drugs could reduce inflammation caused by virus infection. IFN-γ mRNA expression in the trachea of group IV was higher compared with the expression level of other groups from day 1 to 7 post-infection. In addition, pathological damages of the trachea and lung in group IV were less compared with pathological changes of the trachea and lung from other groups. Previous studies reported that viral RNA loads and mRNA expression of IL-1β, IL-6, and TNF-α were upregulated after IBV infection; thereby, pathological damage in tissue was aggravated (33–35). Usually, the viral RNA loads in the trachea and lung decrease significantly at 3–4 days post-challenge. However, in this study, viral loads in the trachea peaked at 5 dpi in all groups and then began to gradually decline, and this finding is the same as the report of He. Their research indicated that viral loads in the trachea peaked at 5 dpi and in the kidney at 8 dpi after IBV infection in SPF chickens (36), the viral loads in the trachea were consistent with our results, and the viral loads in the lung were different, which may be due to the fact that birds used in this study are non-SPF broilers in farms instead of SPF chickens. We speculate that virus replication is slower in non-SPF broiler than in SPF chickens, or SPF chicken is more susceptible to IBV-M41 than non-SPF broiler chicken. Furthermore, in this study, all broilers were vaccinated; this is the main reason why the peak was reached at 5 dpi. There was no significant change in the viral load in the lung, which may be because the two-drug combination blocked the spread of the virus from the trachea to the lung. Accordingly, we concluded that SGDL granule combined with doxycycline alleviated the pathological damage of the trachea and lung caused by IBV infection through lowering viral loads and reducing the mRNA expression of IL-1β, IL-6, and TNF-α cytokines in the trachea and lung. Simultaneously, we speculated that the viral load decrease in trachea may be caused by elevation of interferon expression. Our results also confirmed this hypothesis that the combination of SGDL granules and doxycycline both increased the IFN-γ mRNA expression in the tracheas and lungs compared to doxycycline and SGDL granules individually; this is contrary to the expression of viral loads of IBV in tracheas and lungs.

In conclusion, after being simultaneously administering Shegandilong granule and doxycycline through drinking water, broilers showed a higher anti-IBV antibody level in serum and IgA level in tracheal washes. The combination of Shegandilong granule and doxycycline inhibited the replication of IBV, modulated the production of cytokines, and reduced histopathological lesions caused by IBV infection in the trachea and lung. These data suggested that the combination of Shegandilong granule and doxycycline is a novel approach to effectively preventing avian IB in broilers.

## Data Availability Statement

The original contributions presented in the study are included in the article/Supplementary Material, further inquiries can be directed to the corresponding authors.

## Ethics Statement

The animal study was reviewed and approved by the Animal Care and Use Committee of Lanzhou Institute of Husbandry and Pharmaceutical Sciences of the Chinese Academy of Agricultural Sciences.

## Author Contributions

JL conceived and designed the project. HF and XW wrote and reviewed the manuscript. HF, JZ, RX, WZ, KaiZ, KanZ, LW, ZG, ZQ, and GW performed the animal experiments. All authors have read and agreed to the published version of the manuscript.

## Funding

Financial support of this study was supported by the National Key Research and Development Program of China (No. 2017YFE0114400), the Agricultural Sciences and Technology Innovation Program (ASTIP-2020), major output research topics of the Chinese Academy of Agricultural Sciences (CAAS-ZDXT2018008), and Key Research and Development Fund of Gansu Province (20YF8FA030).

## Conflict of Interest

The authors declare that the research was conducted in the absence of any commercial or financial relationships that could be construed as a potential conflict of interest.

## Publisher's Note

All claims expressed in this article are solely those of the authors and do not necessarily represent those of their affiliated organizations, or those of the publisher, the editors and the reviewers. Any product that may be evaluated in this article, or claim that may be made by its manufacturer, is not guaranteed or endorsed by the publisher.
