# Supplementary figures and images for: Combined Effect of Shegandilong Granule and Doxycycline on Immune Responses and Protection Against Avian Infectious Bronchitis Virus in Broilers
Source: Front Vet Sci. 2021 Dec 20;8:756629. doi: 10.3389/fvets.2021.756629 (PMC8721878; doi:10.3389/fvets.2021.756629)

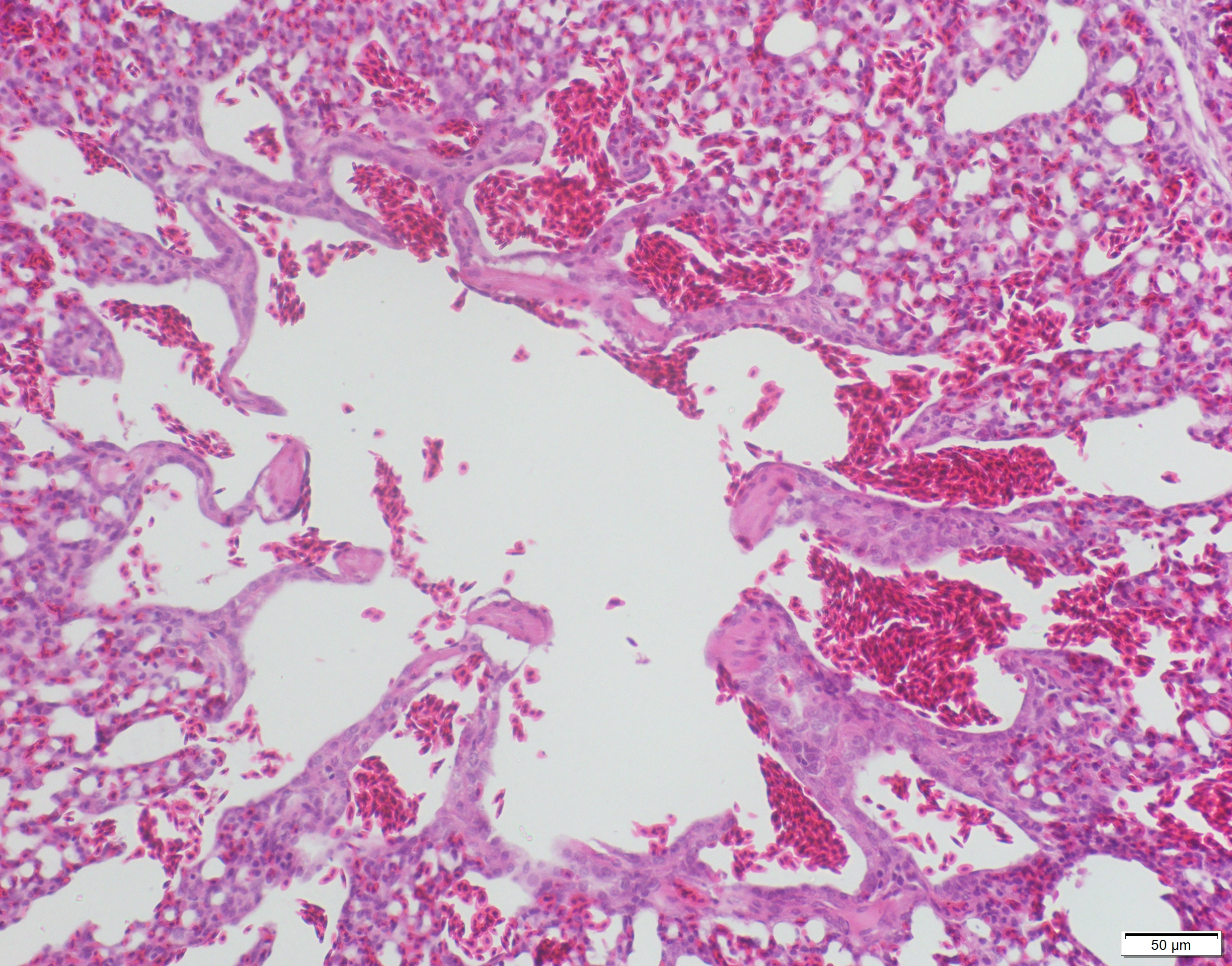

Supplement: Supplementary file 2 [file Data_Sheet_2.ZIP › ╨┬╜¿╬─╝■╝╨ (2)/groupI 5dpi.jpg]

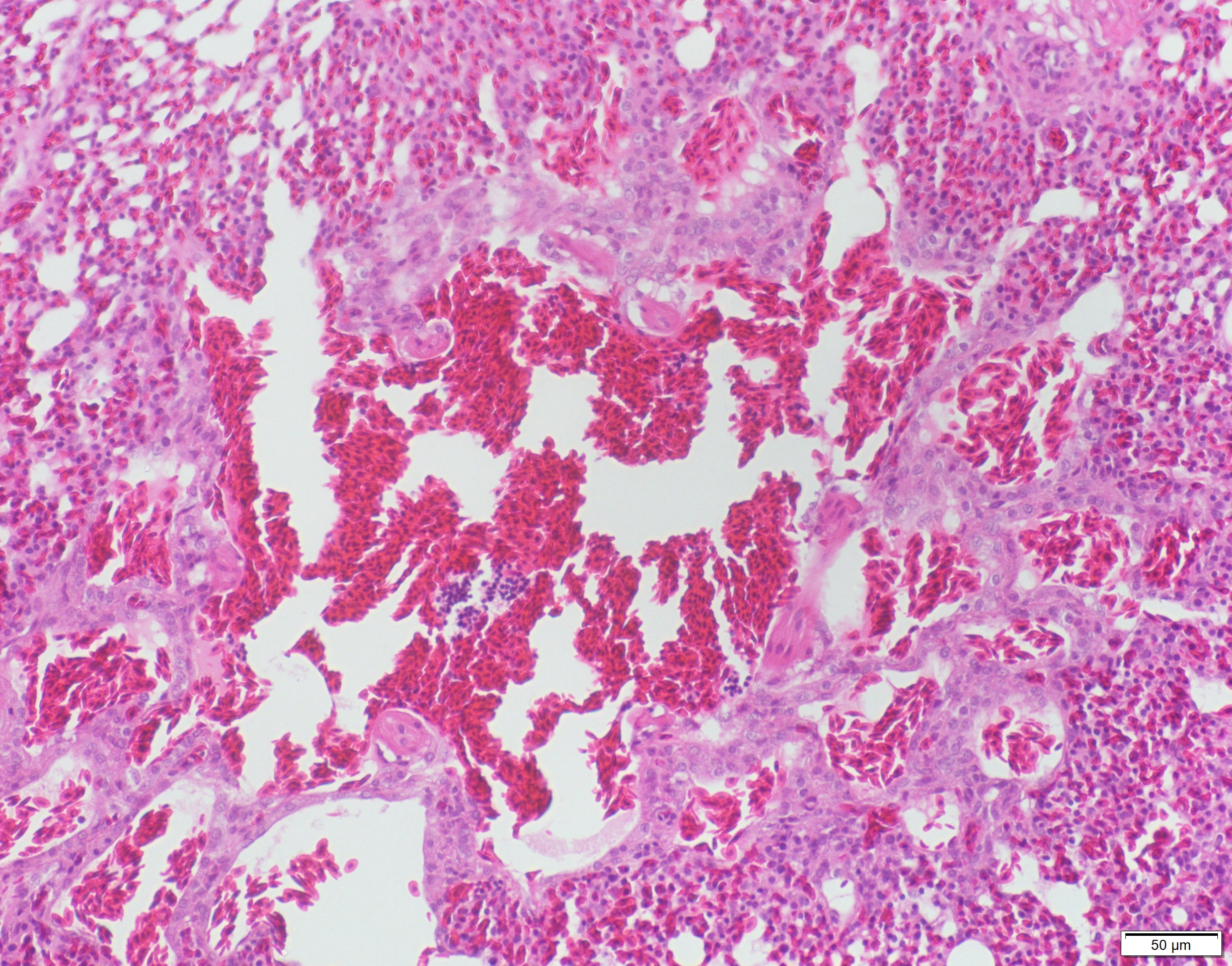

Supplement: Supplementary file 2 [file Data_Sheet_2.ZIP › ╨┬╜¿╬─╝■╝╨ (2)/groupI 1dpi.jpg]

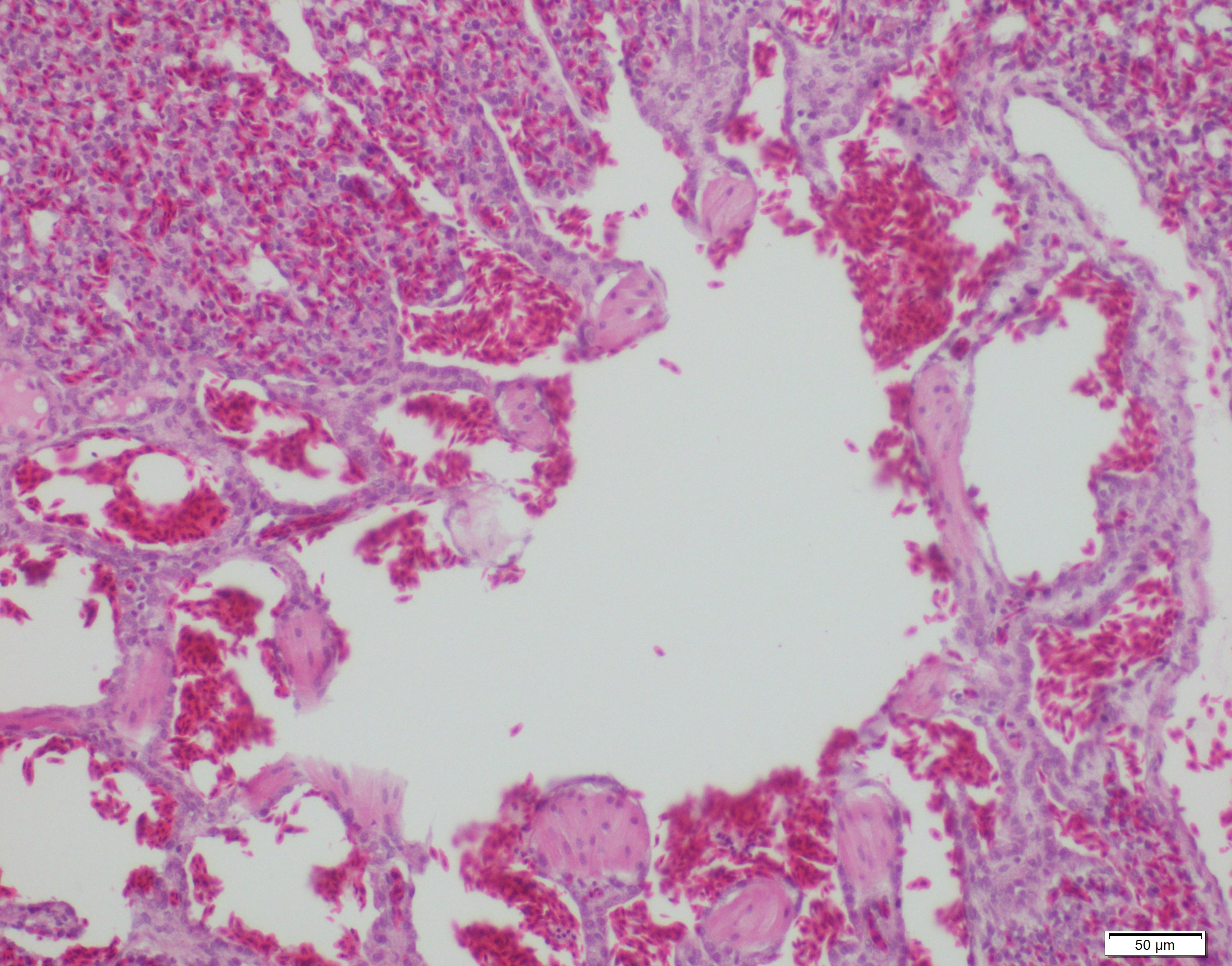

Supplement: Supplementary file 2 [file Data_Sheet_2.ZIP › ╨┬╜¿╬─╝■╝╨ (2)/groupI 3dpi.jpg]

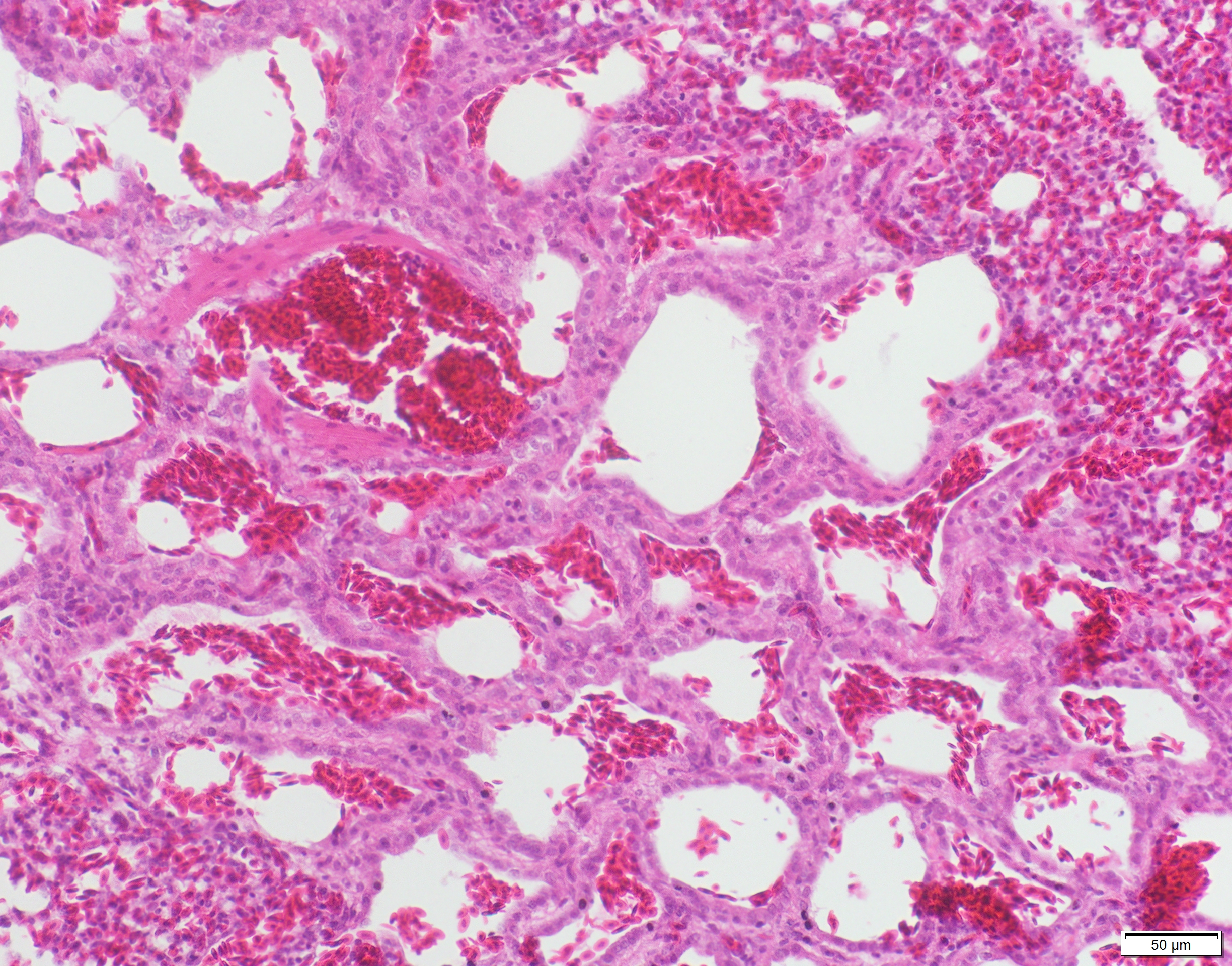

Supplement: Supplementary file 2 [file Data_Sheet_2.ZIP › ╨┬╜¿╬─╝■╝╨ (2)/groupI 7dpi.jpg]

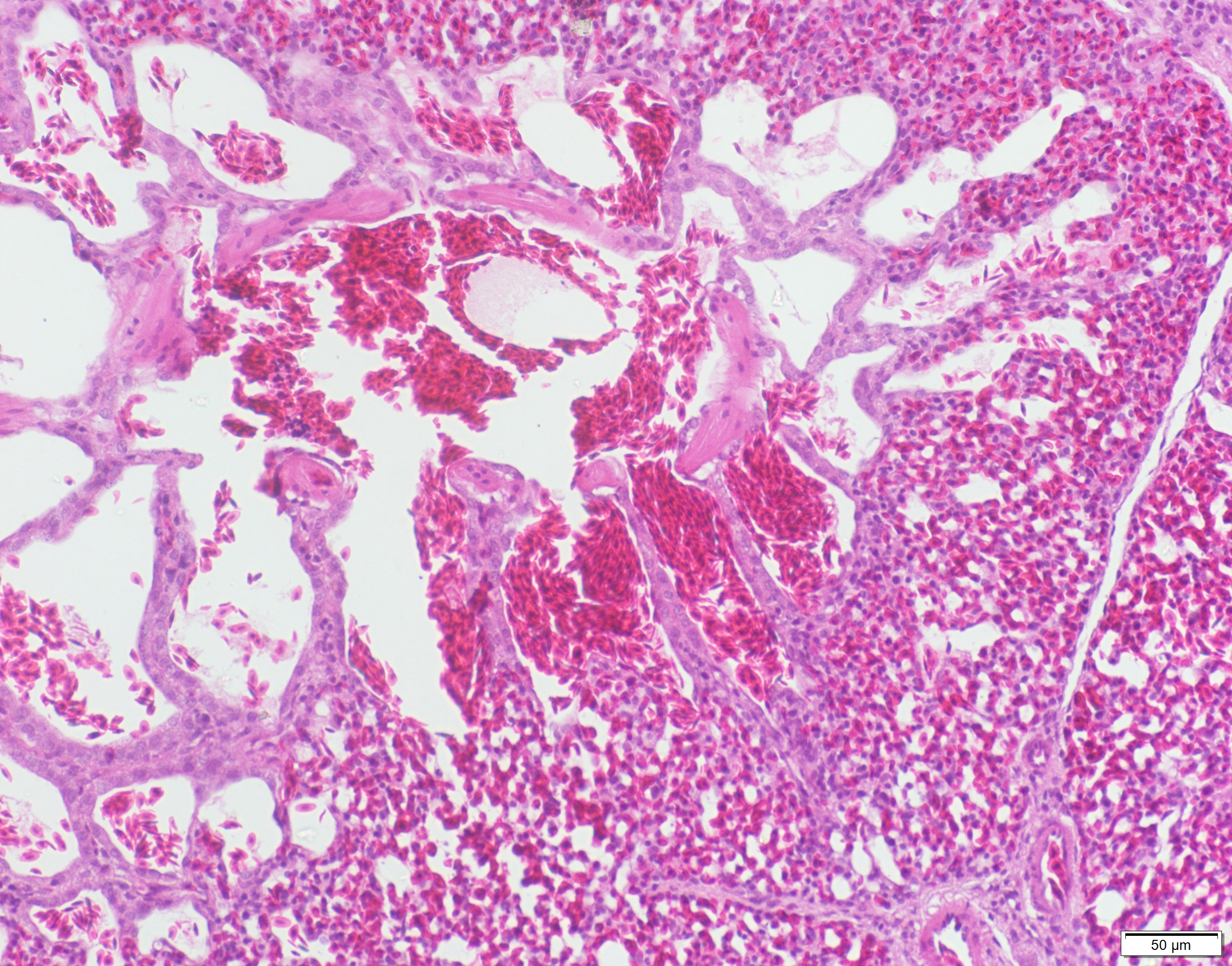

Supplement: Supplementary file 2 [file Data_Sheet_2.ZIP › ╨┬╜¿╬─╝■╝╨ (2)/groupII 1dpi.jpg]

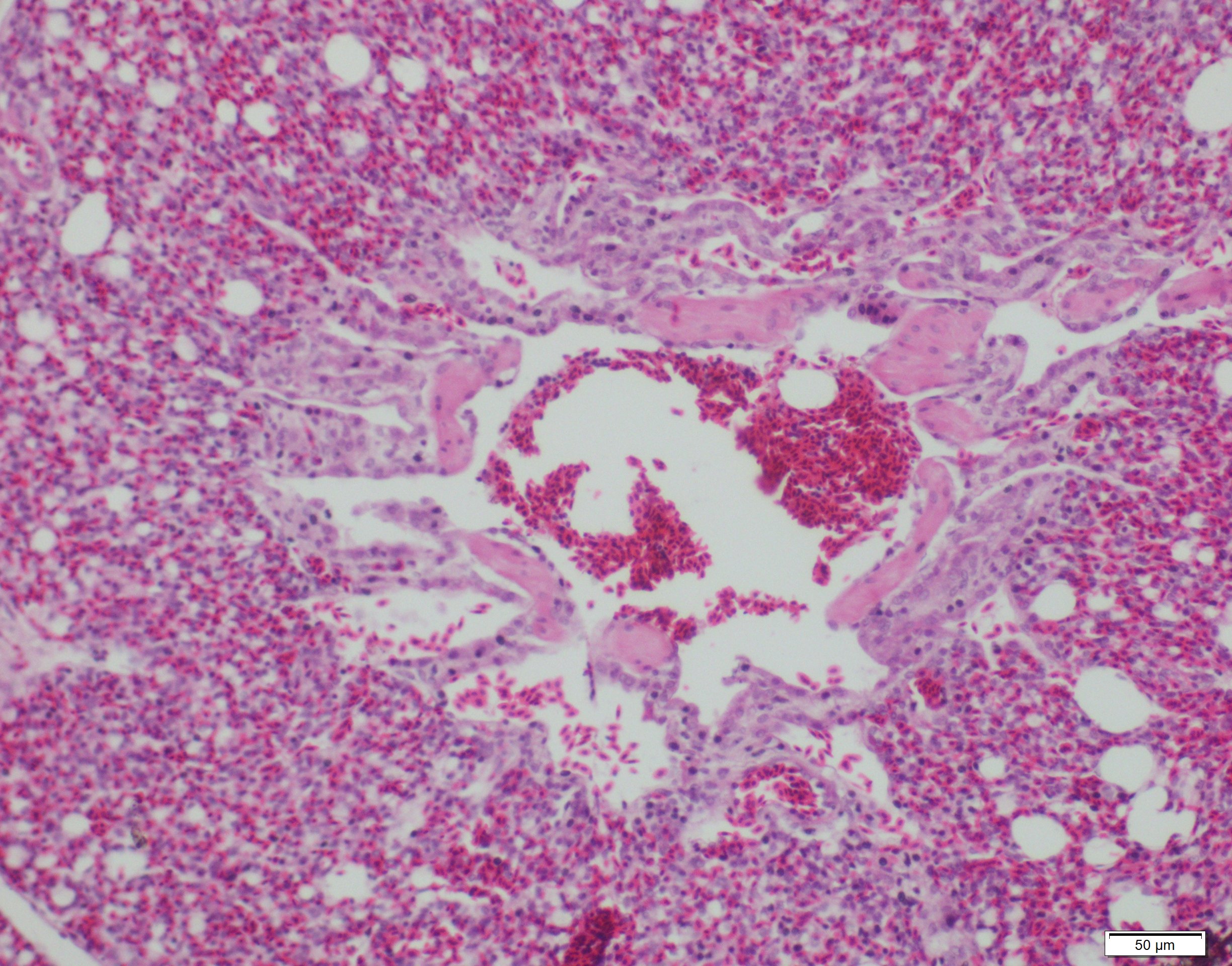

Supplement: Supplementary file 2 [file Data_Sheet_2.ZIP › ╨┬╜¿╬─╝■╝╨ (2)/groupII 3dpi.jpg]

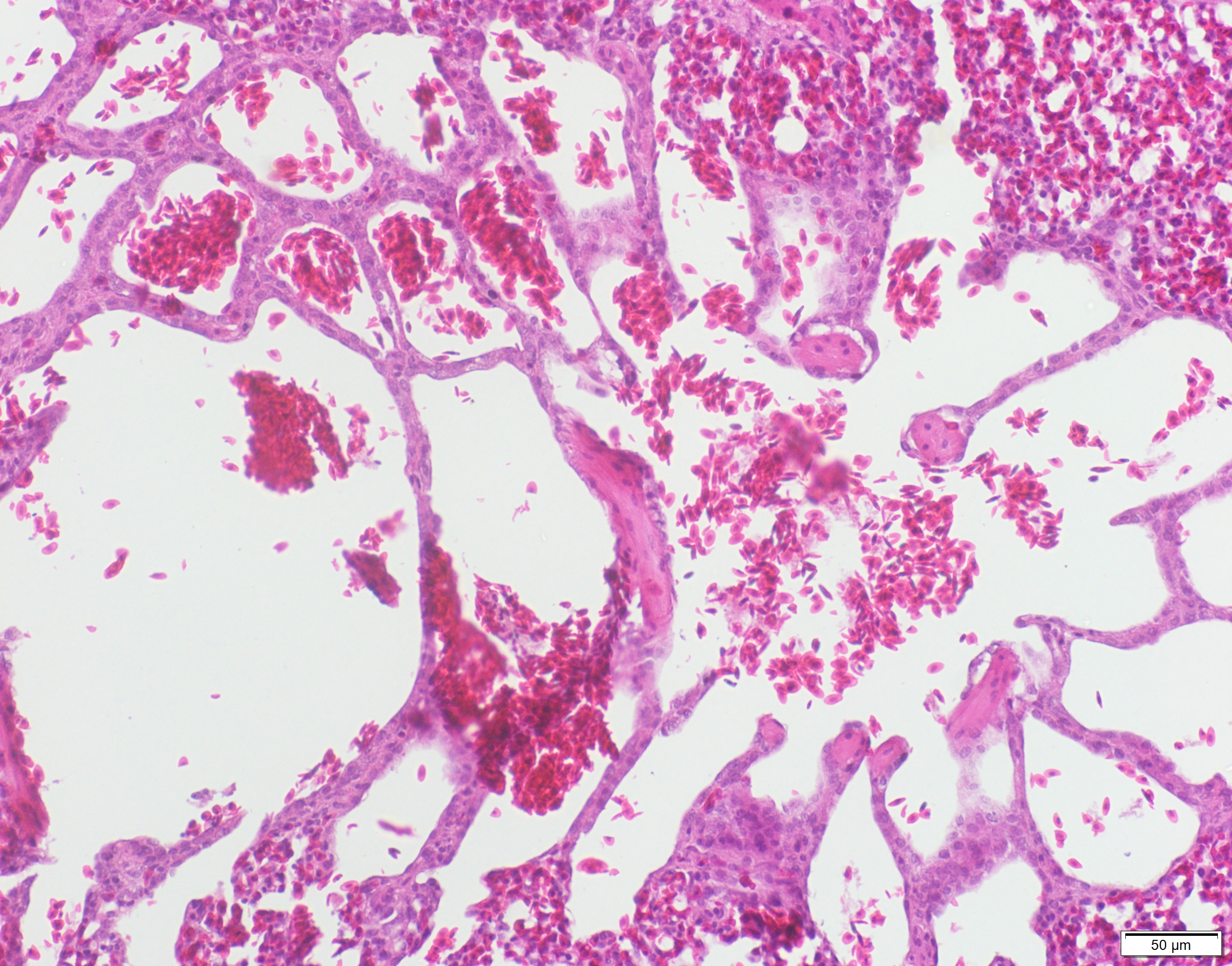

Supplement: Supplementary file 2 [file Data_Sheet_2.ZIP › ╨┬╜¿╬─╝■╝╨ (2)/groupII 5dpi.jpg]

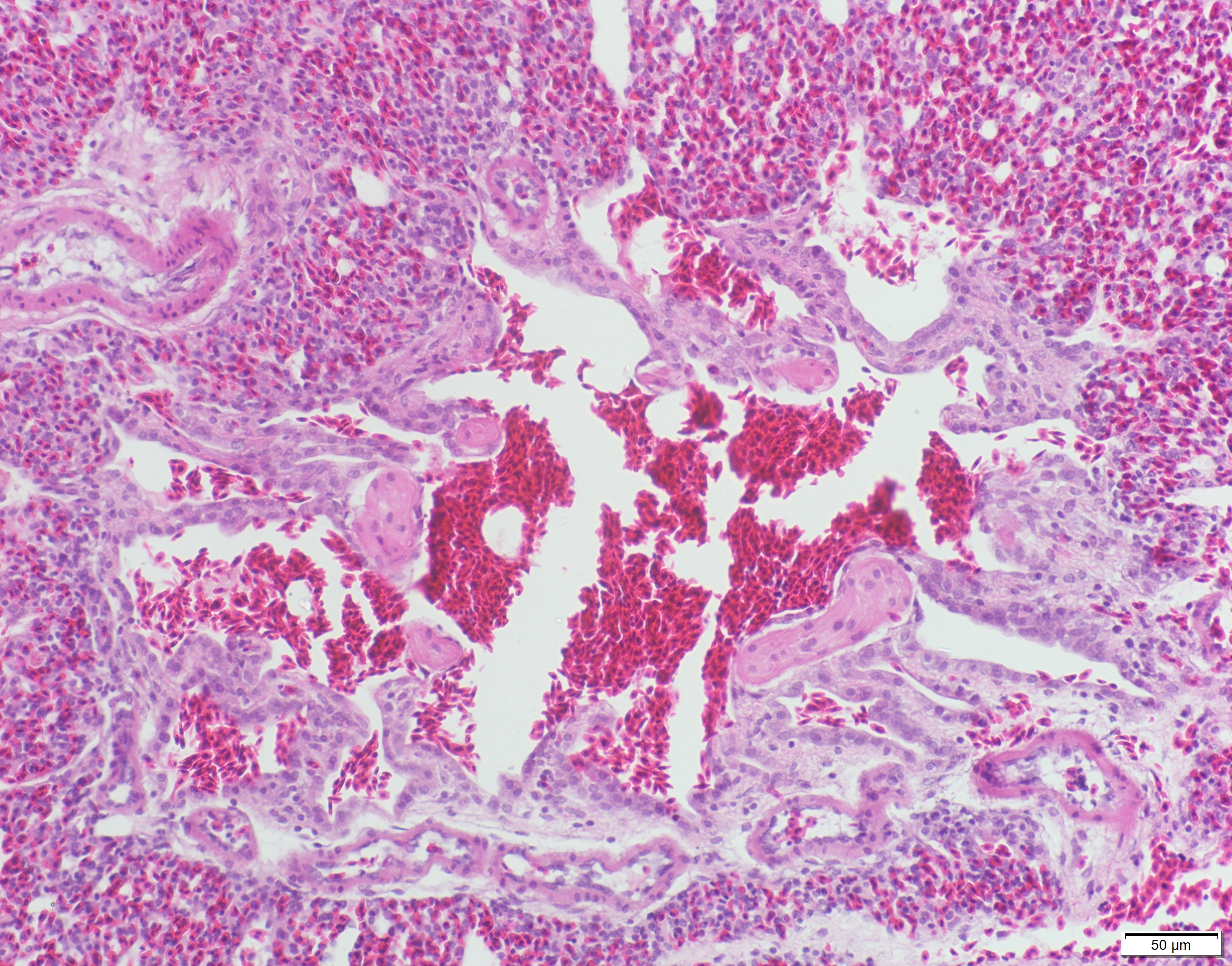

Supplement: Supplementary file 2 [file Data_Sheet_2.ZIP › ╨┬╜¿╬─╝■╝╨ (2)/groupII 7dpi.jpg]

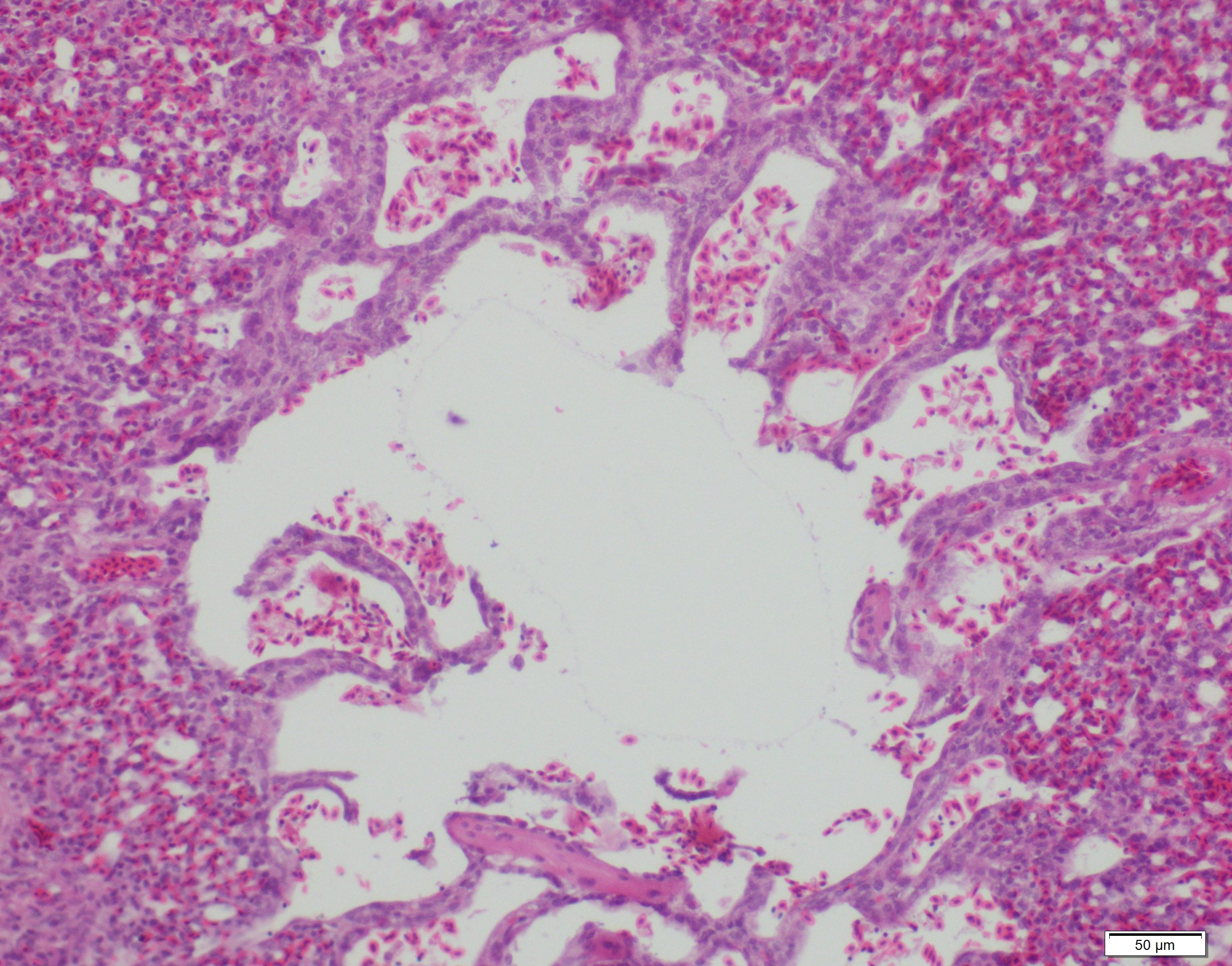

Supplement: Supplementary file 3 [file Data_Sheet_3.ZIP › ╨┬╜¿╬─╝■╝╨/group IV 3dpi.jpg]

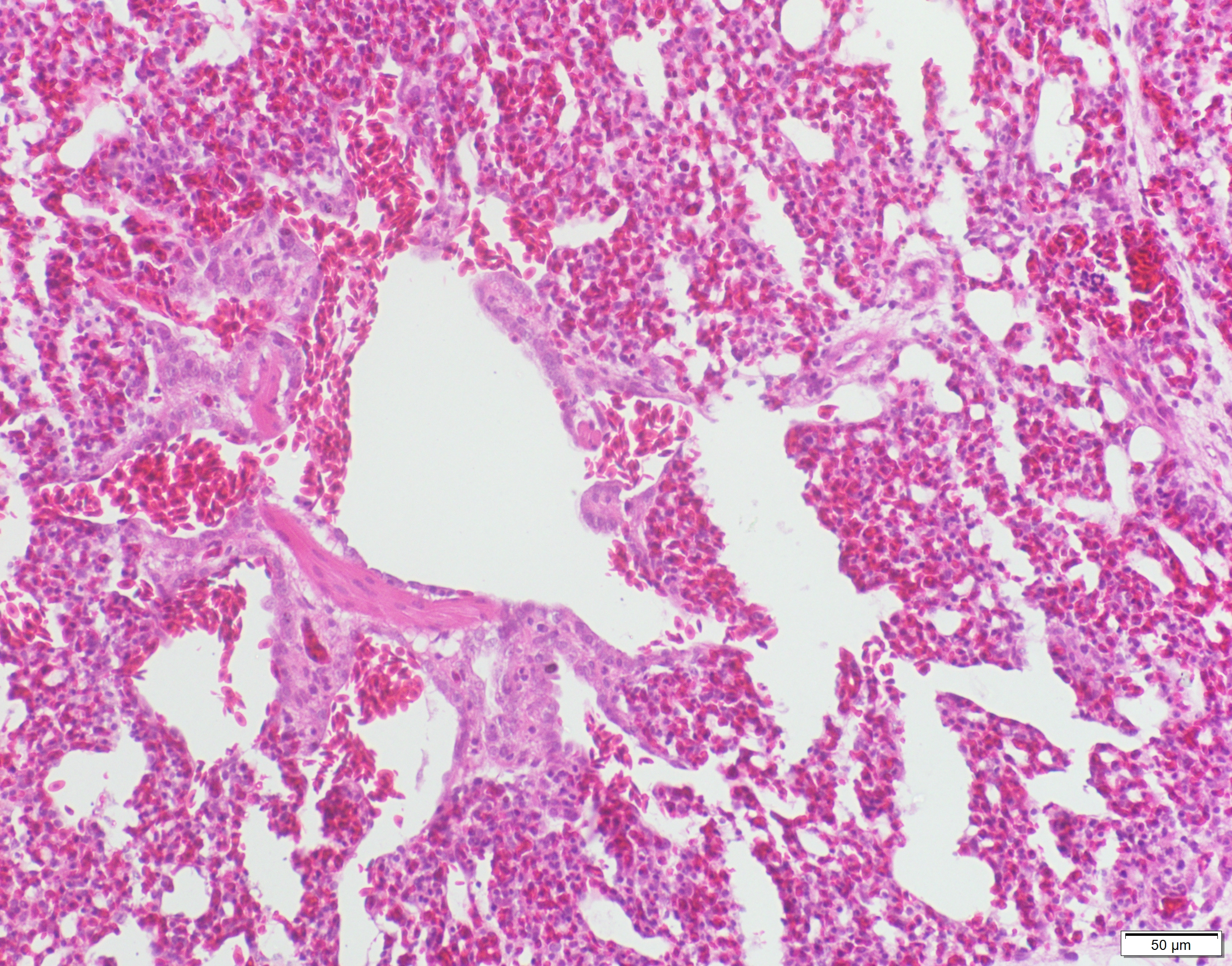

Supplement: Supplementary file 3 [file Data_Sheet_3.ZIP › ╨┬╜¿╬─╝■╝╨/group IV 5dpi.jpg]

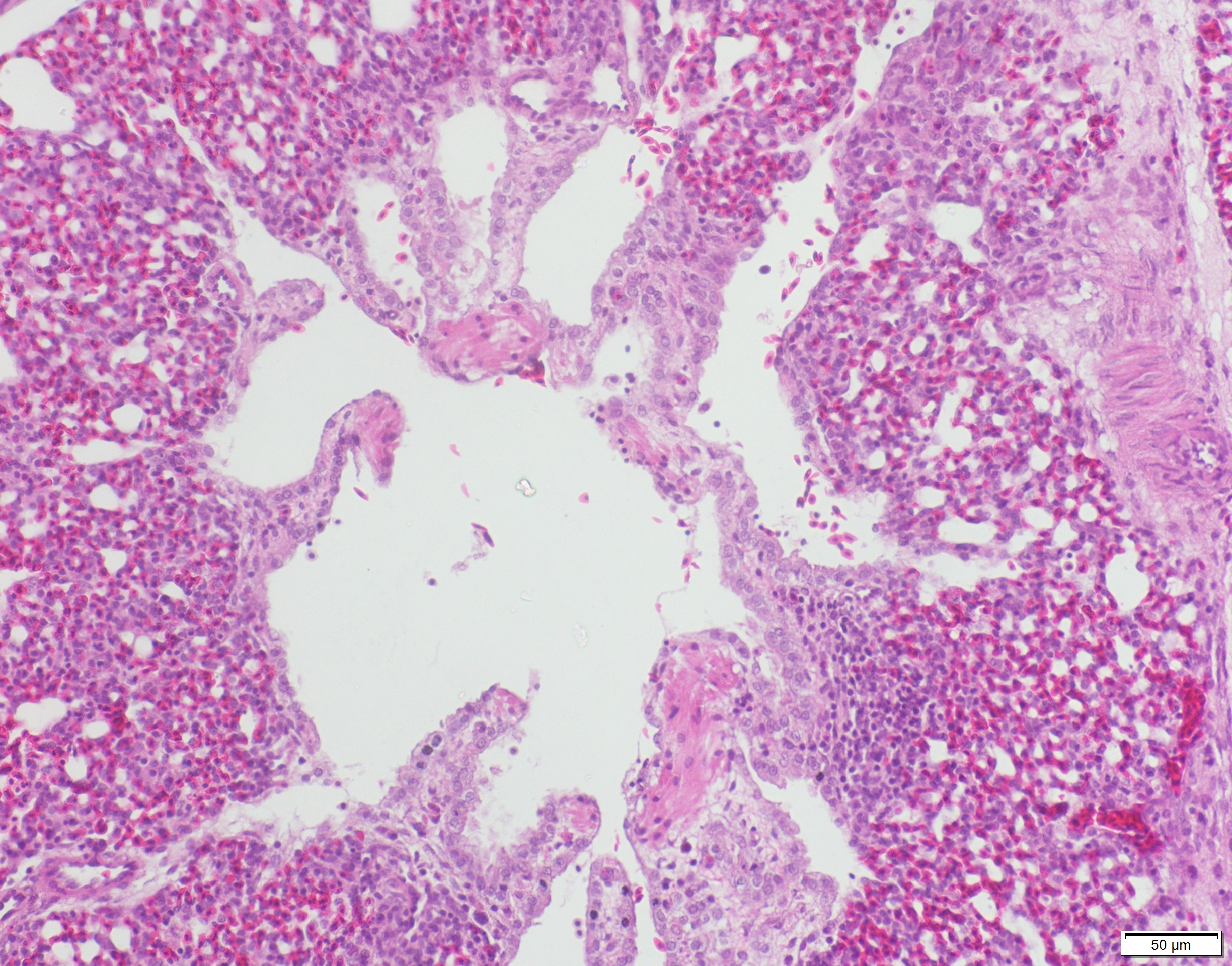

Supplement: Supplementary file 3 [file Data_Sheet_3.ZIP › ╨┬╜¿╬─╝■╝╨/group IV 7dpi.jpg]

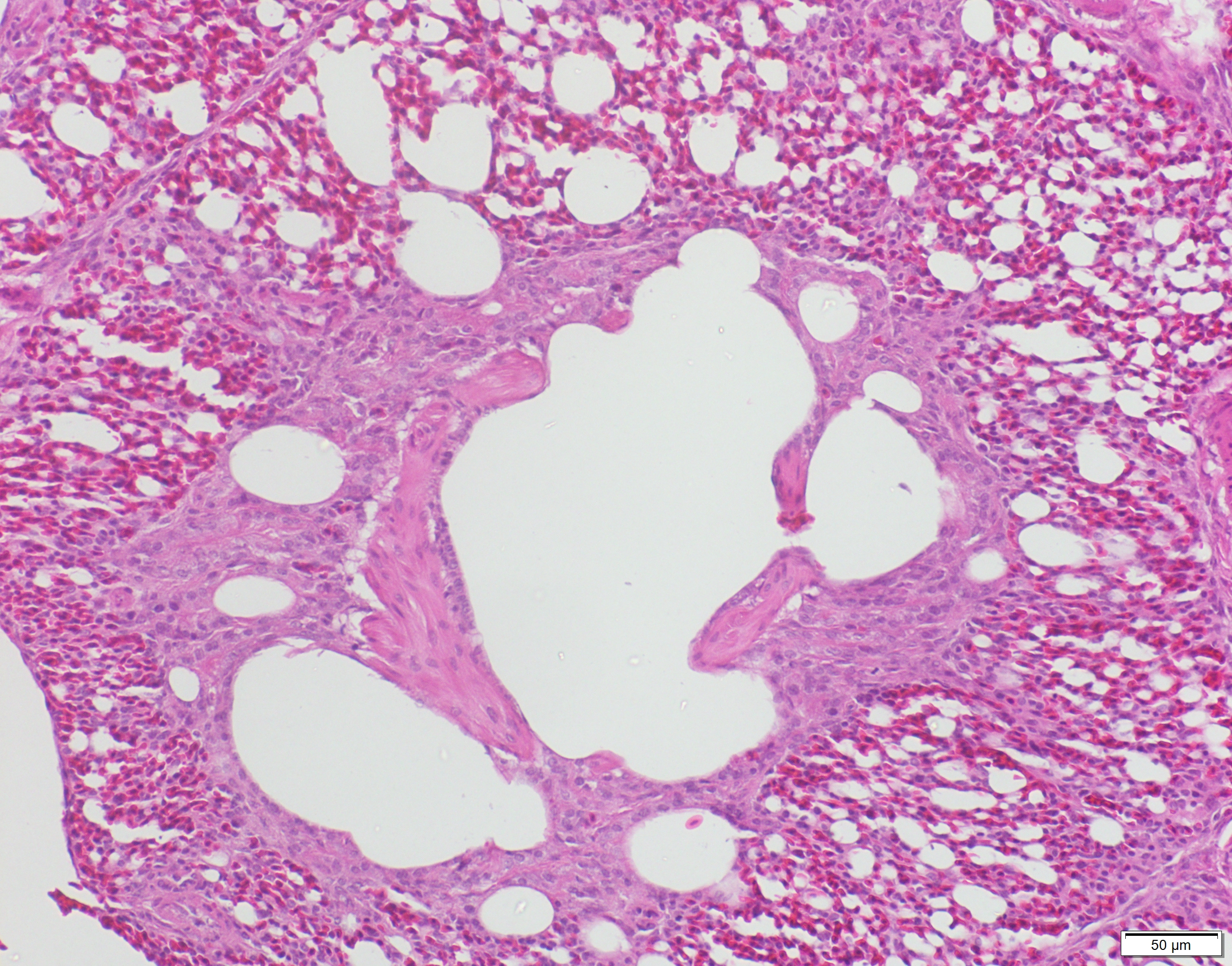

Supplement: Supplementary file 3 [file Data_Sheet_3.ZIP › ╨┬╜¿╬─╝■╝╨/group IV 1dpi.jpg]

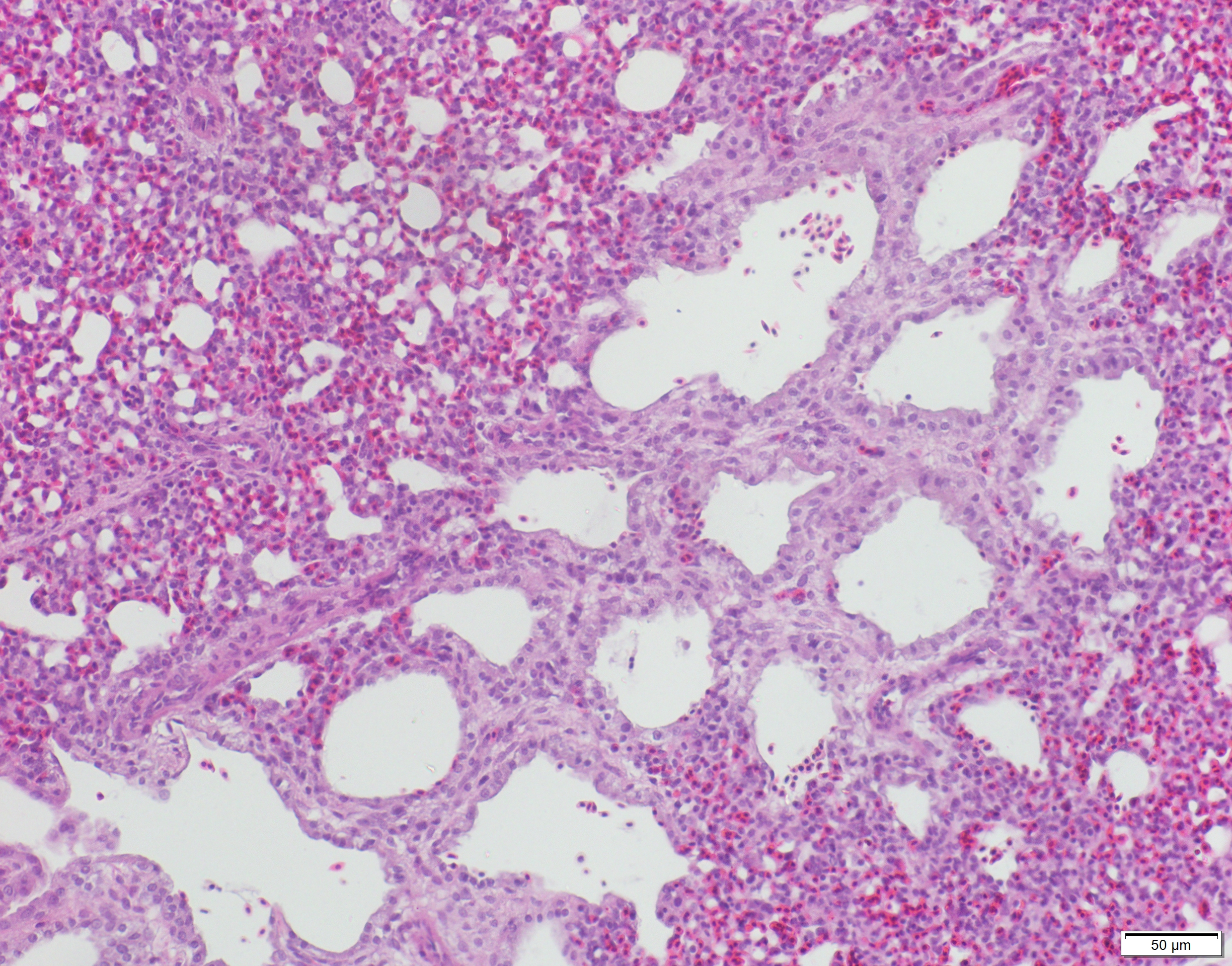

Supplement: Supplementary file 3 [file Data_Sheet_3.ZIP › ╨┬╜¿╬─╝■╝╨/groupIII 1dpi.jpg]

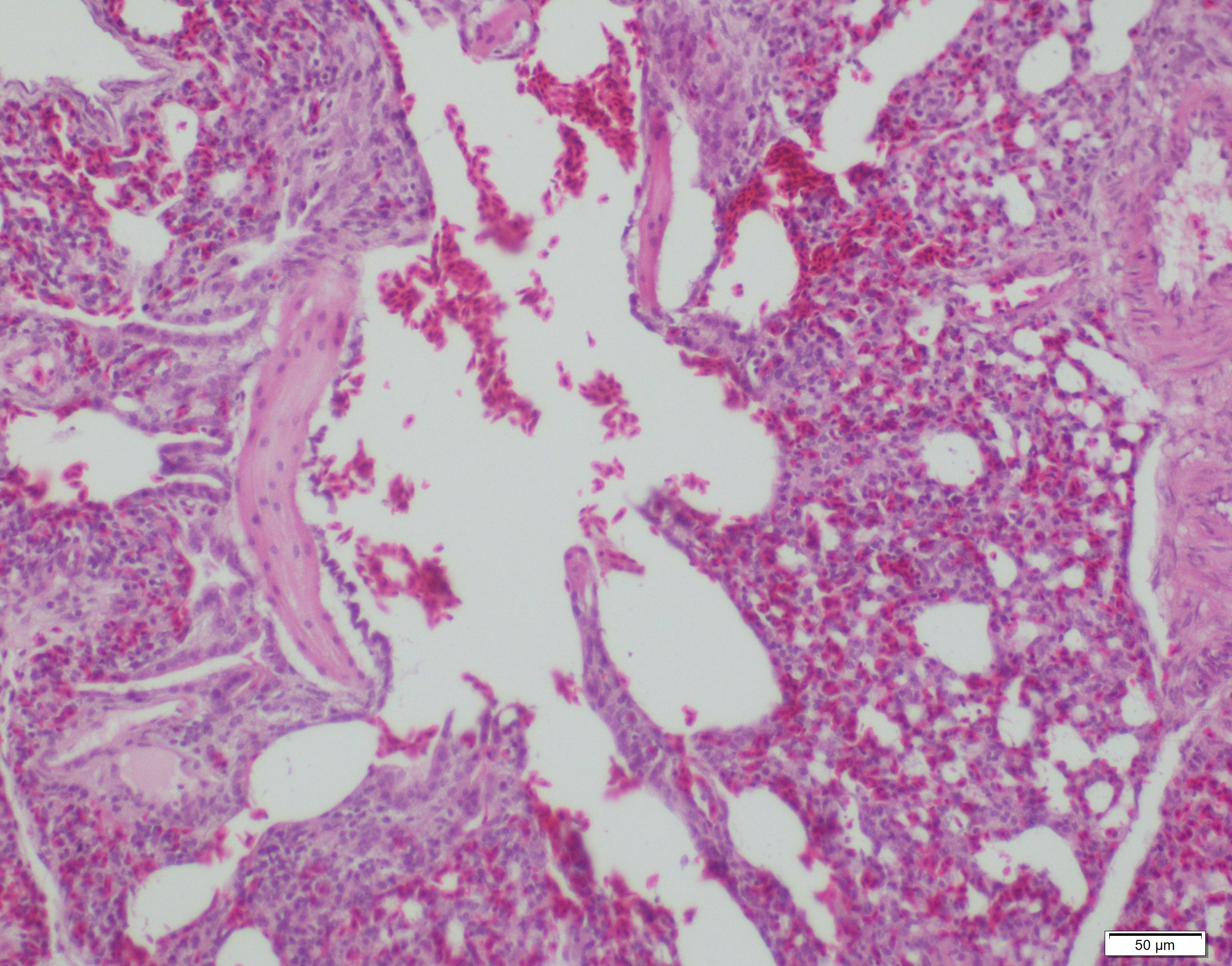

Supplement: Supplementary file 3 [file Data_Sheet_3.ZIP › ╨┬╜¿╬─╝■╝╨/groupIII 3dpi.jpg]

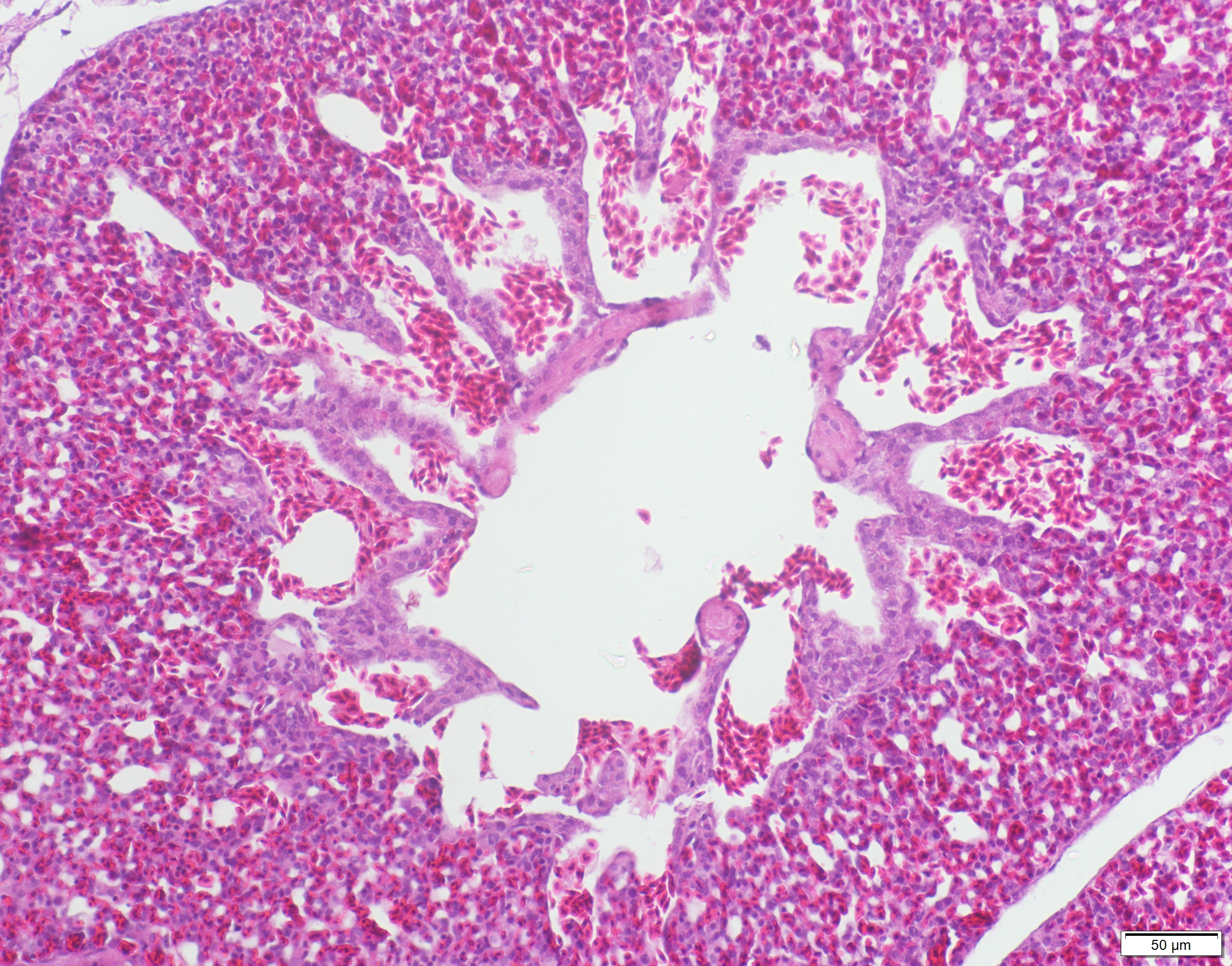

Supplement: Supplementary file 3 [file Data_Sheet_3.ZIP › ╨┬╜¿╬─╝■╝╨/groupIII 5dpi.jpg]

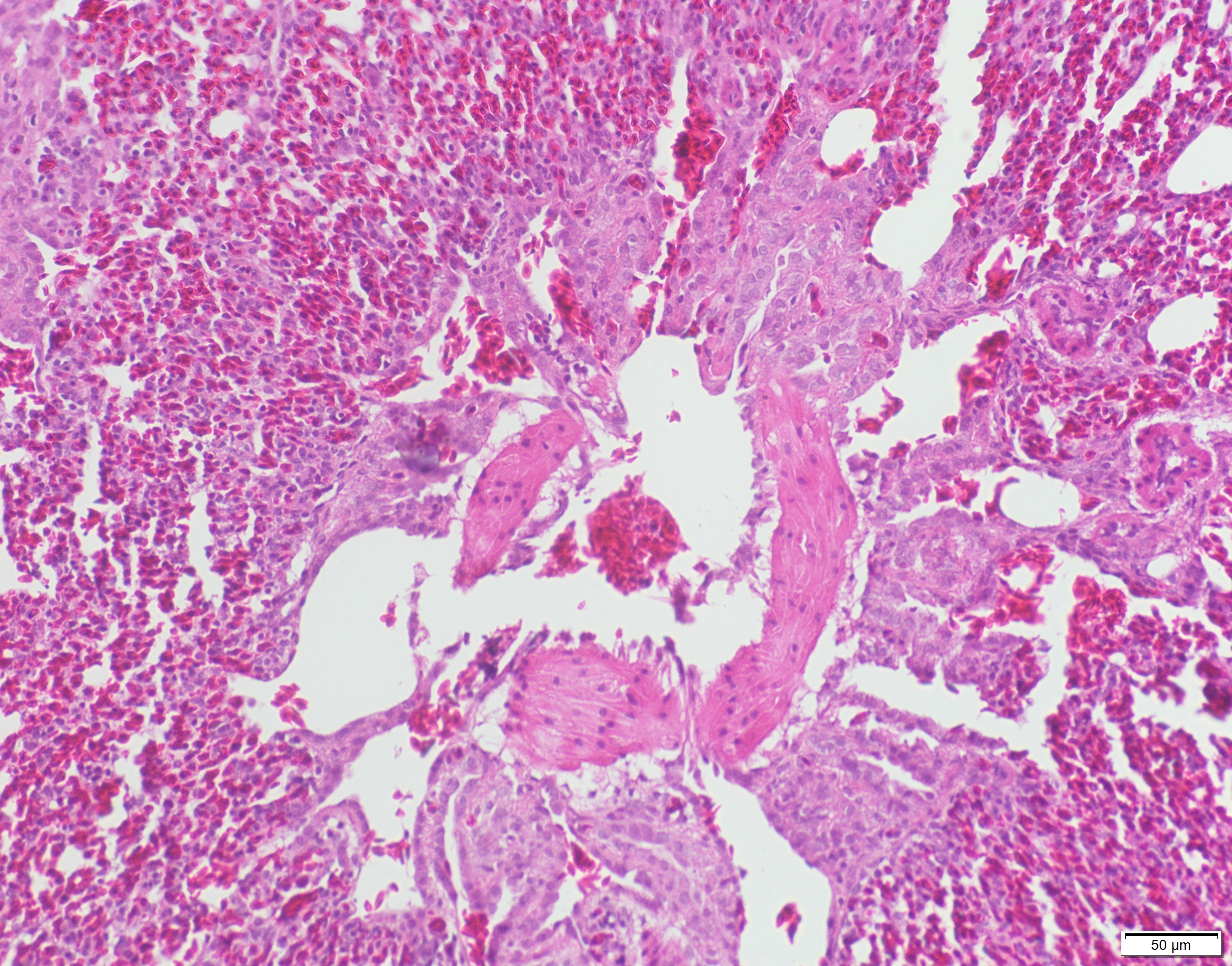

Supplement: Supplementary file 3 [file Data_Sheet_3.ZIP › ╨┬╜¿╬─╝■╝╨/groupIII 7dpi.jpg]

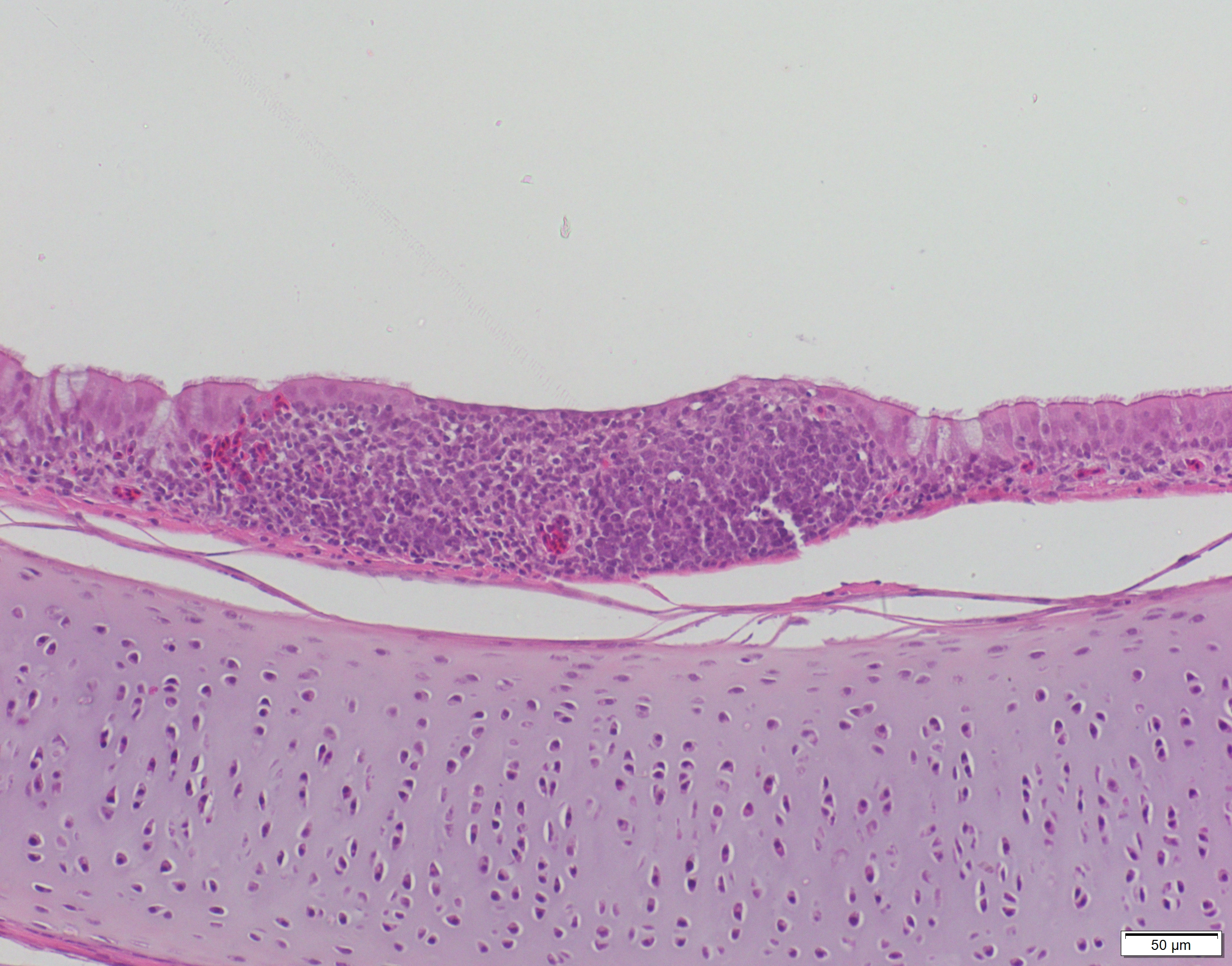

Supplement: Supplementary file 4 [file Data_Sheet_4.ZIP › Histopathological changes of trachea1/groupI 1dpi.jpg]

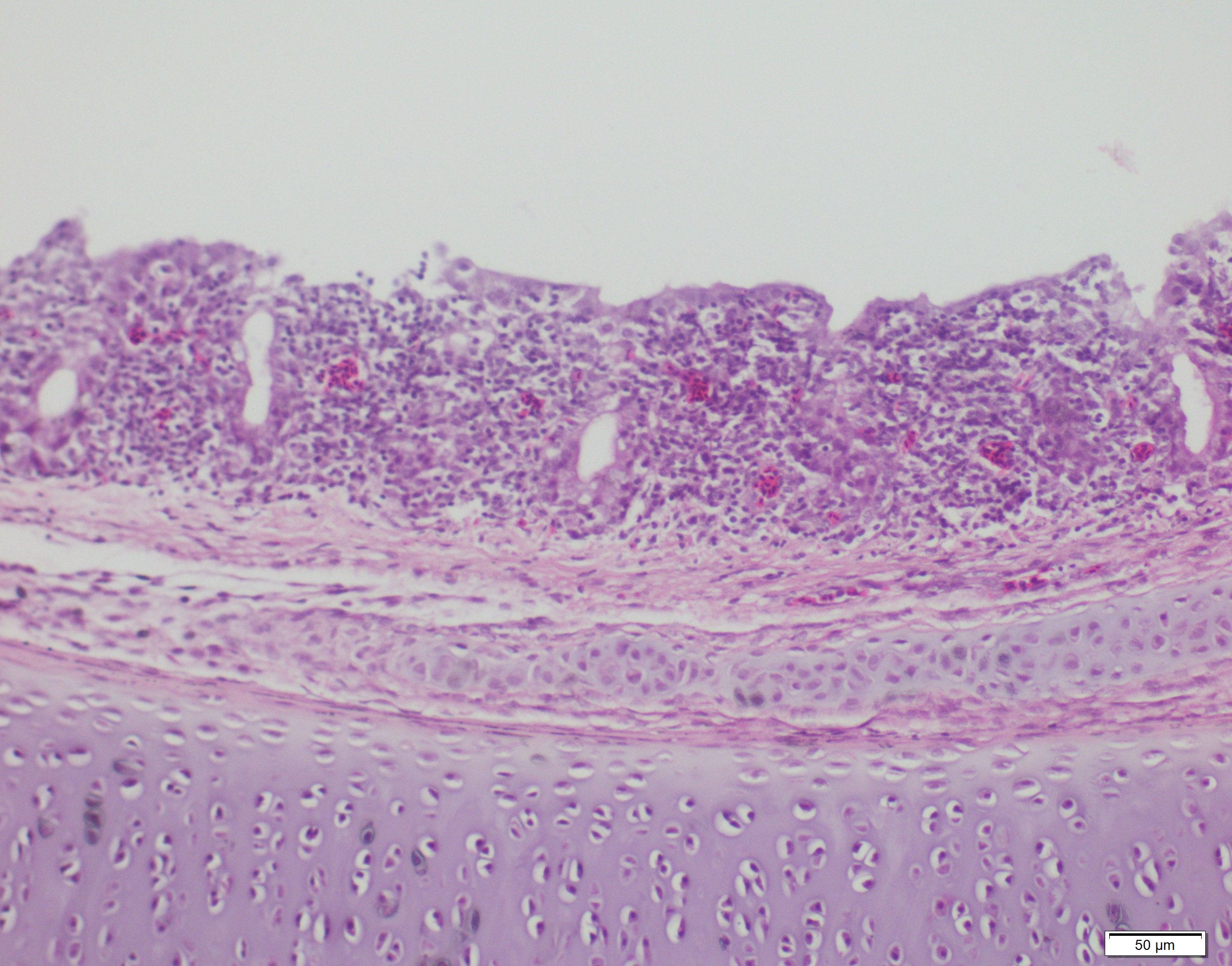

Supplement: Supplementary file 4 [file Data_Sheet_4.ZIP › Histopathological changes of trachea1/groupI 3dpi.jpg]

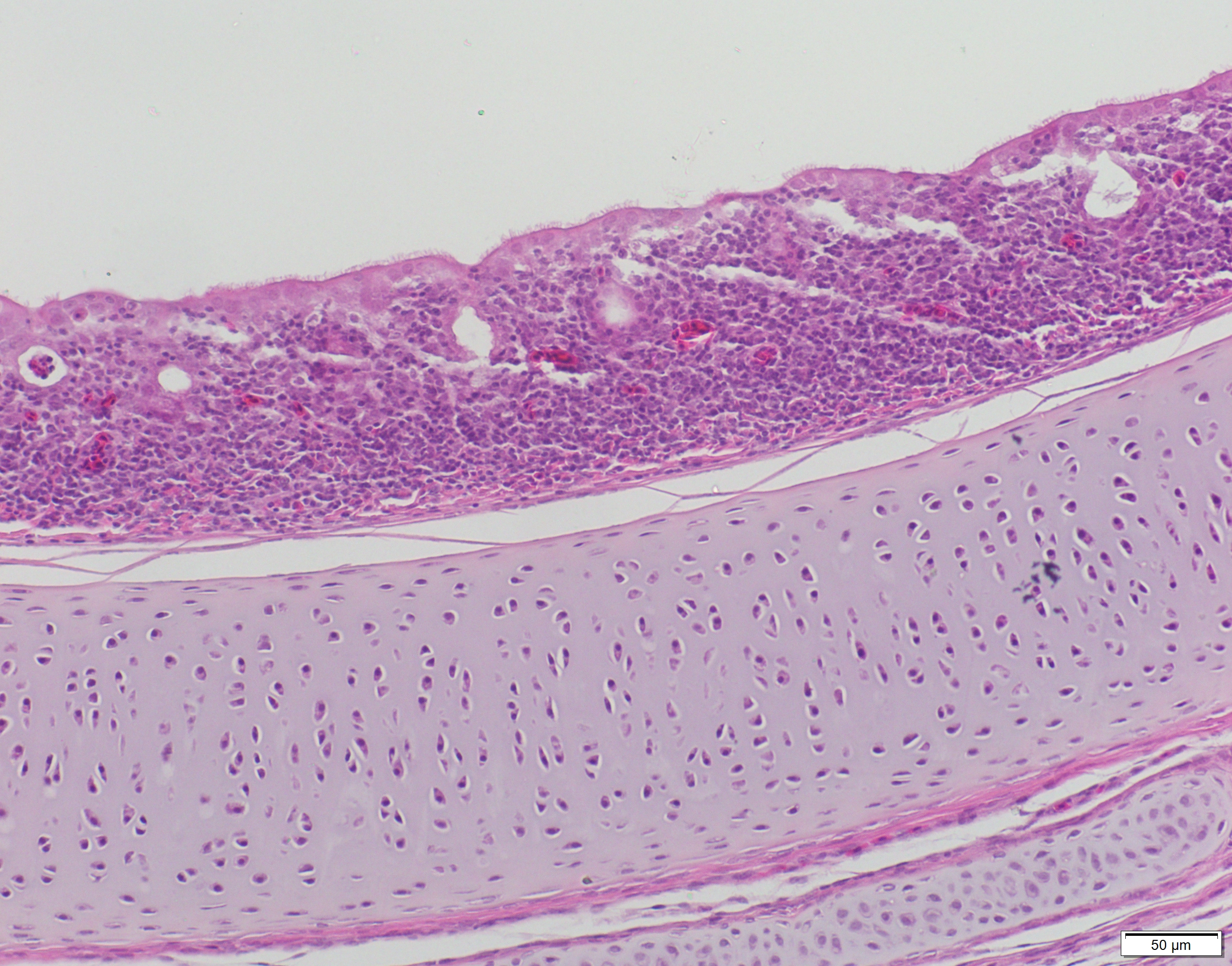

Supplement: Supplementary file 4 [file Data_Sheet_4.ZIP › Histopathological changes of trachea1/groupI 5dpi.jpg]

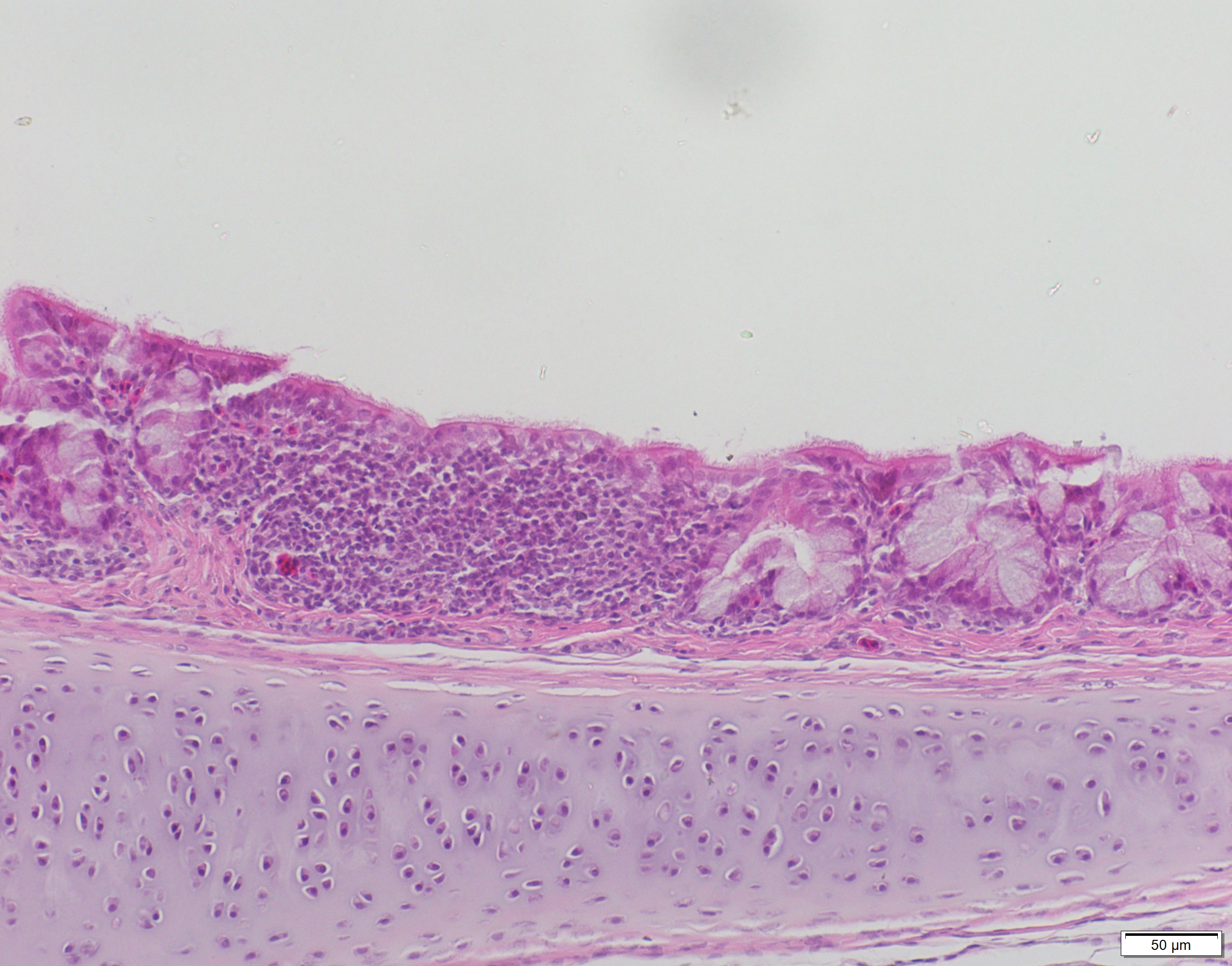

Supplement: Supplementary file 4 [file Data_Sheet_4.ZIP › Histopathological changes of trachea1/groupI 7dpi.jpg]

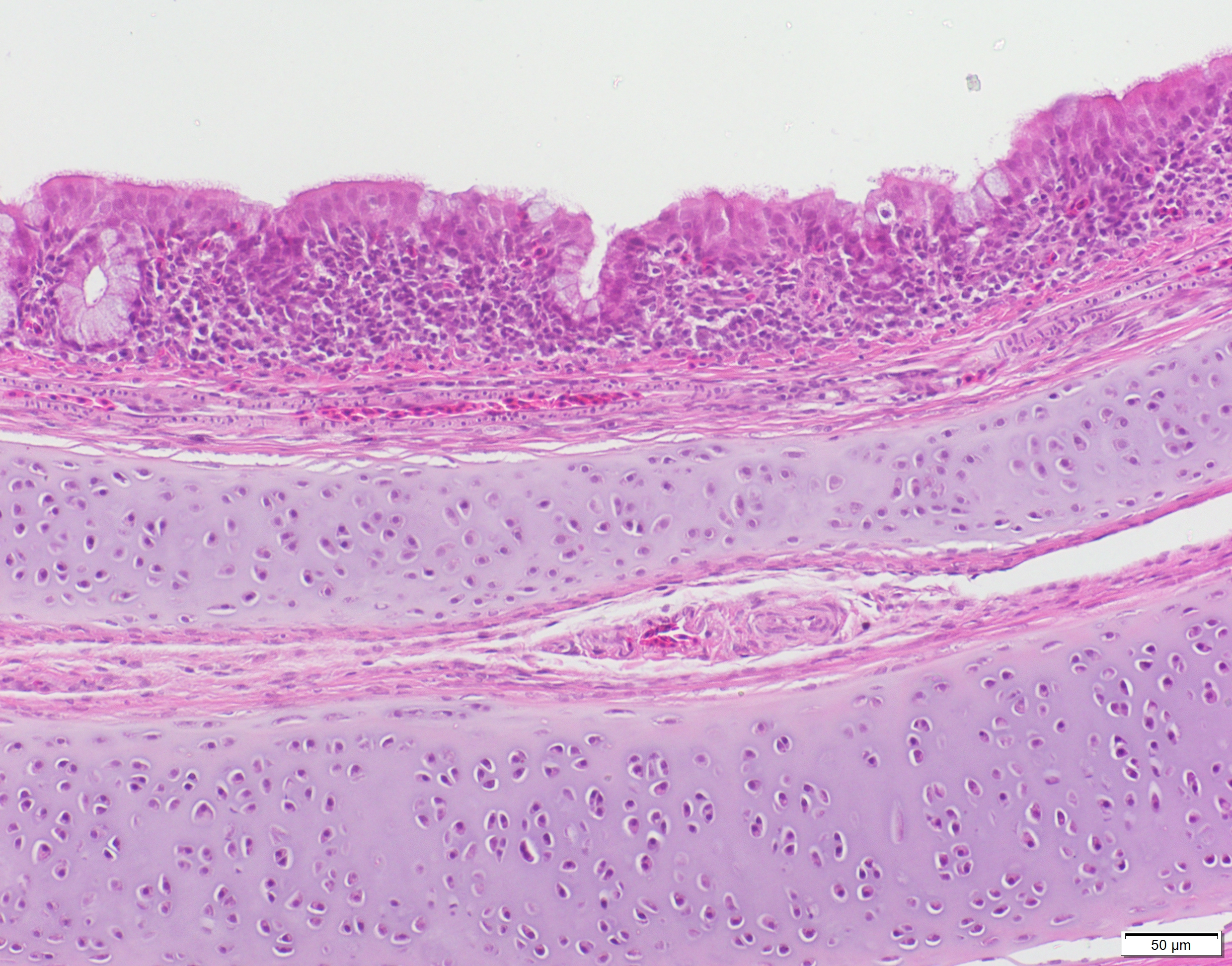

Supplement: Supplementary file 4 [file Data_Sheet_4.ZIP › Histopathological changes of trachea1/groupII 1dpi.jpg]

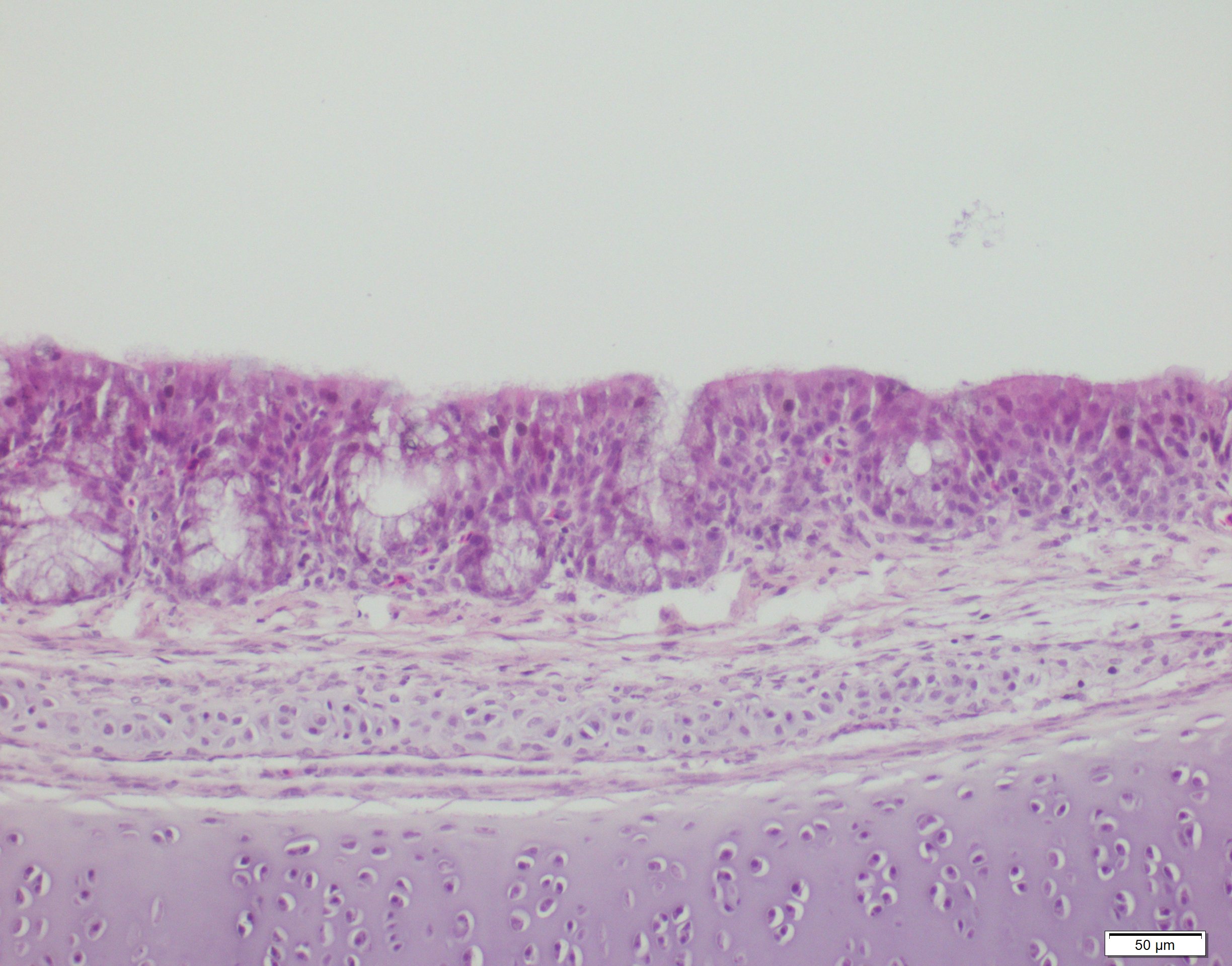

Supplement: Supplementary file 4 [file Data_Sheet_4.ZIP › Histopathological changes of trachea1/groupII 3dpi.jpg]

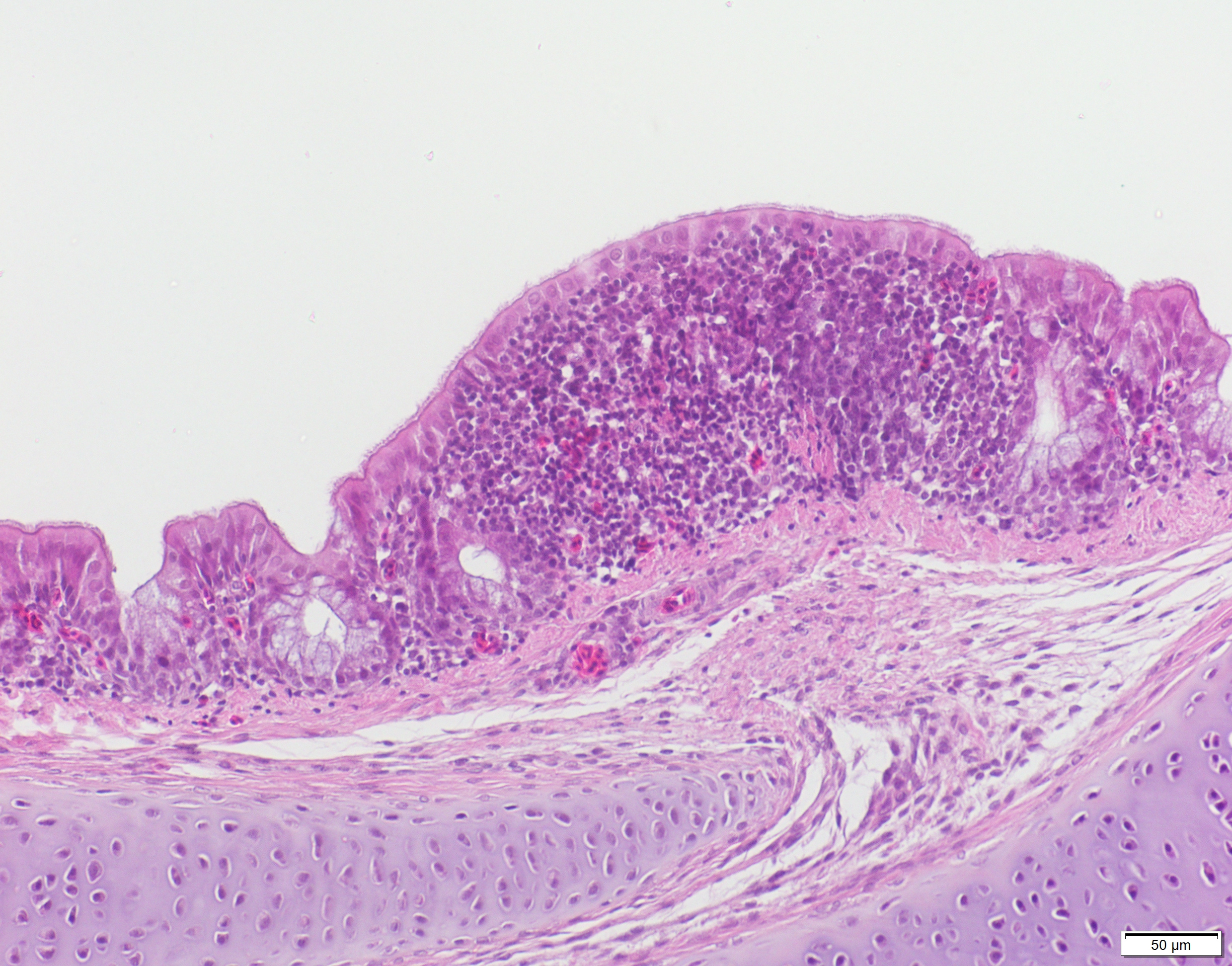

Supplement: Supplementary file 4 [file Data_Sheet_4.ZIP › Histopathological changes of trachea1/groupII 5dpi.jpg]

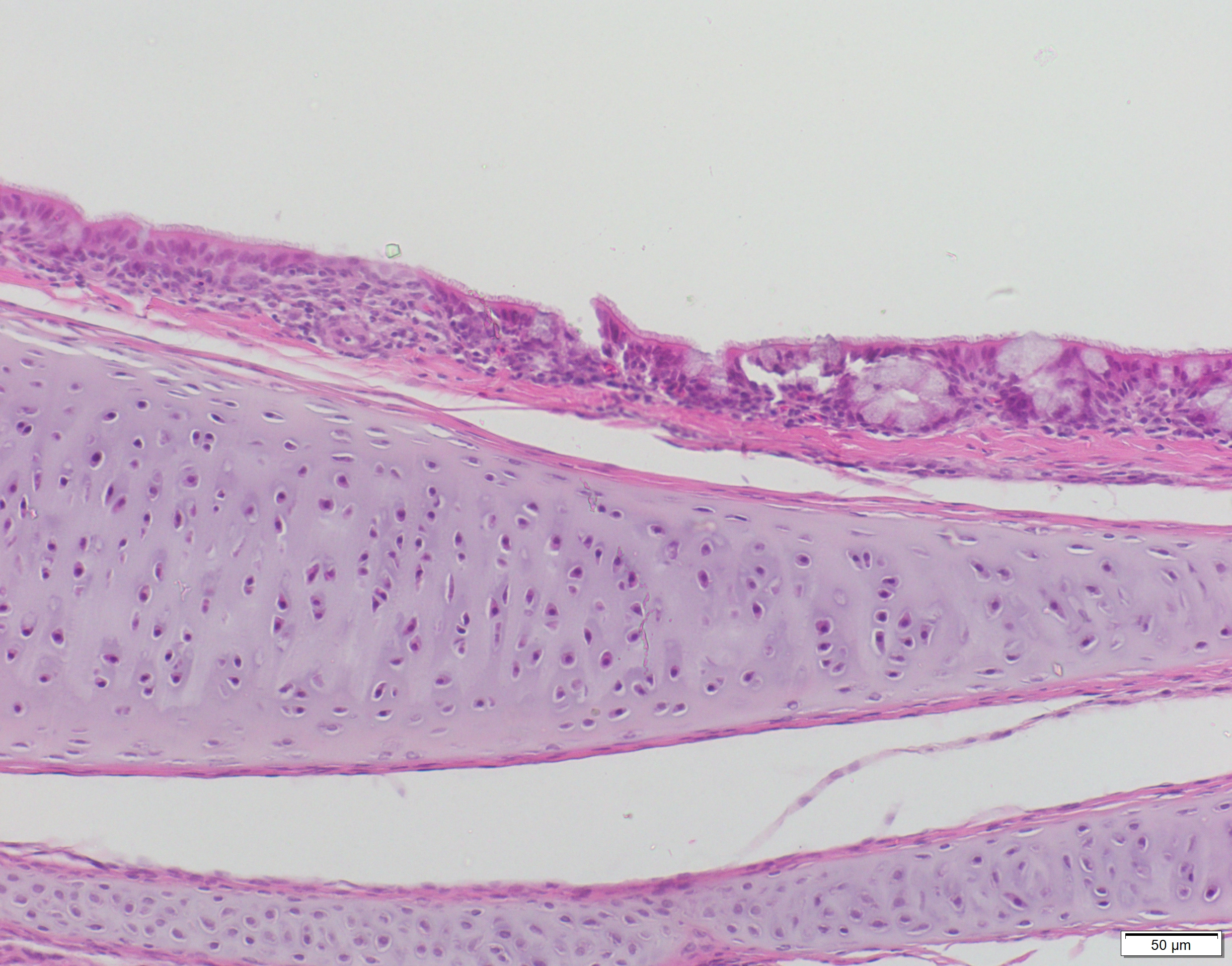

Supplement: Supplementary file 4 [file Data_Sheet_4.ZIP › Histopathological changes of trachea1/groupII 7dpi.jpg]

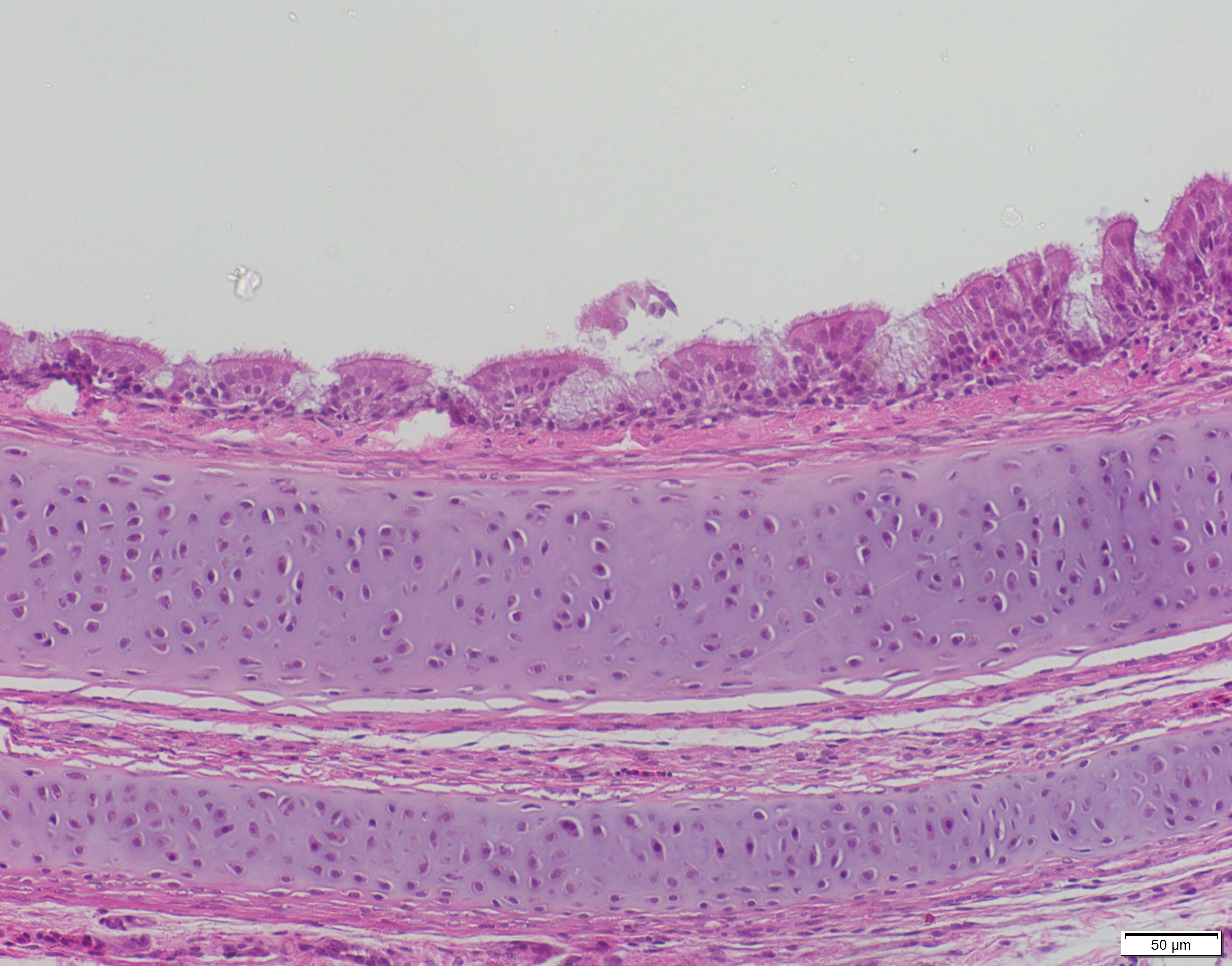

Supplement: Supplementary file 5 [file Data_Sheet_5.ZIP › Histopathological changes of trachea2/groupIII 1dpi.jpg]

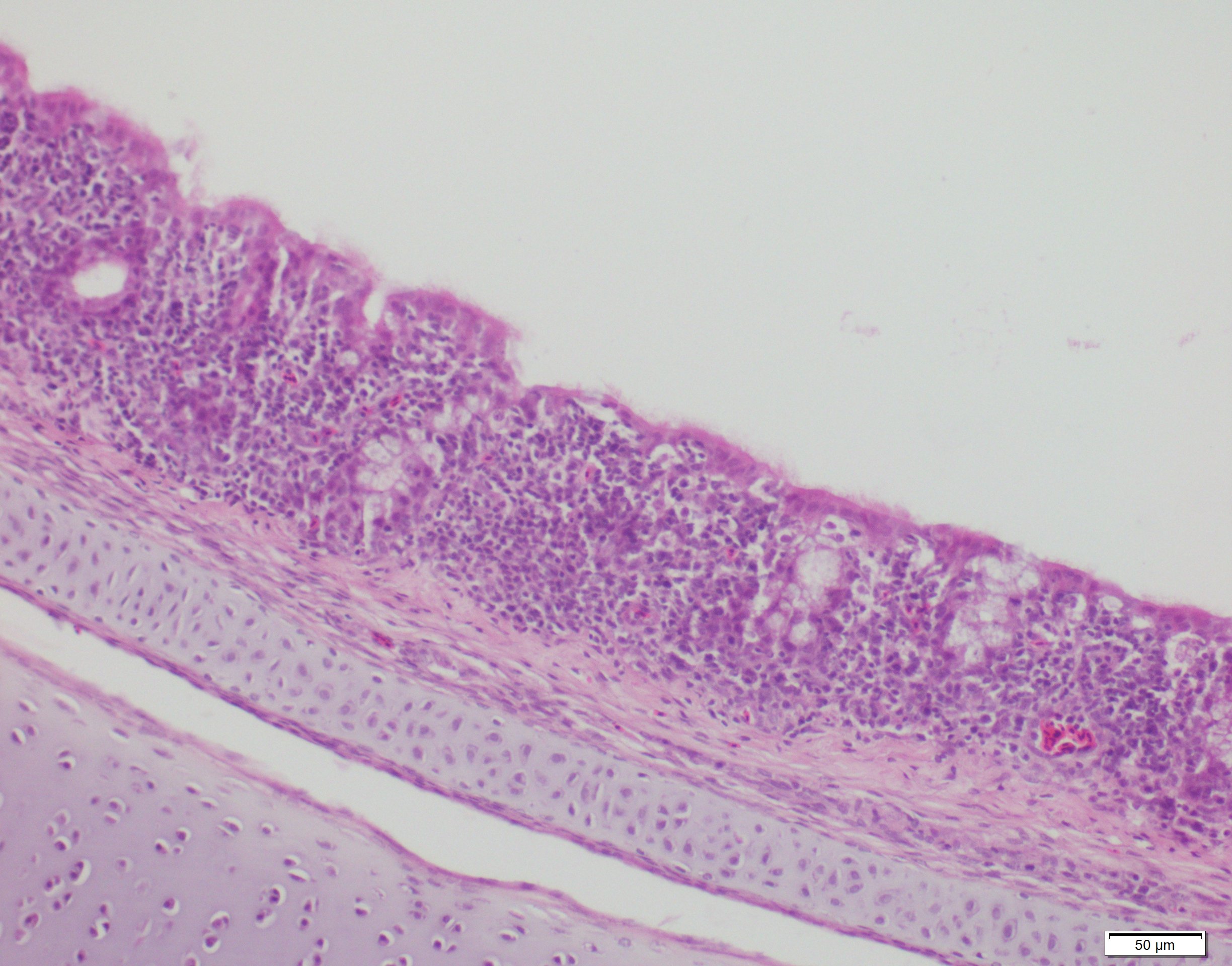

Supplement: Supplementary file 5 [file Data_Sheet_5.ZIP › Histopathological changes of trachea2/groupIII 3dpi.jpg]

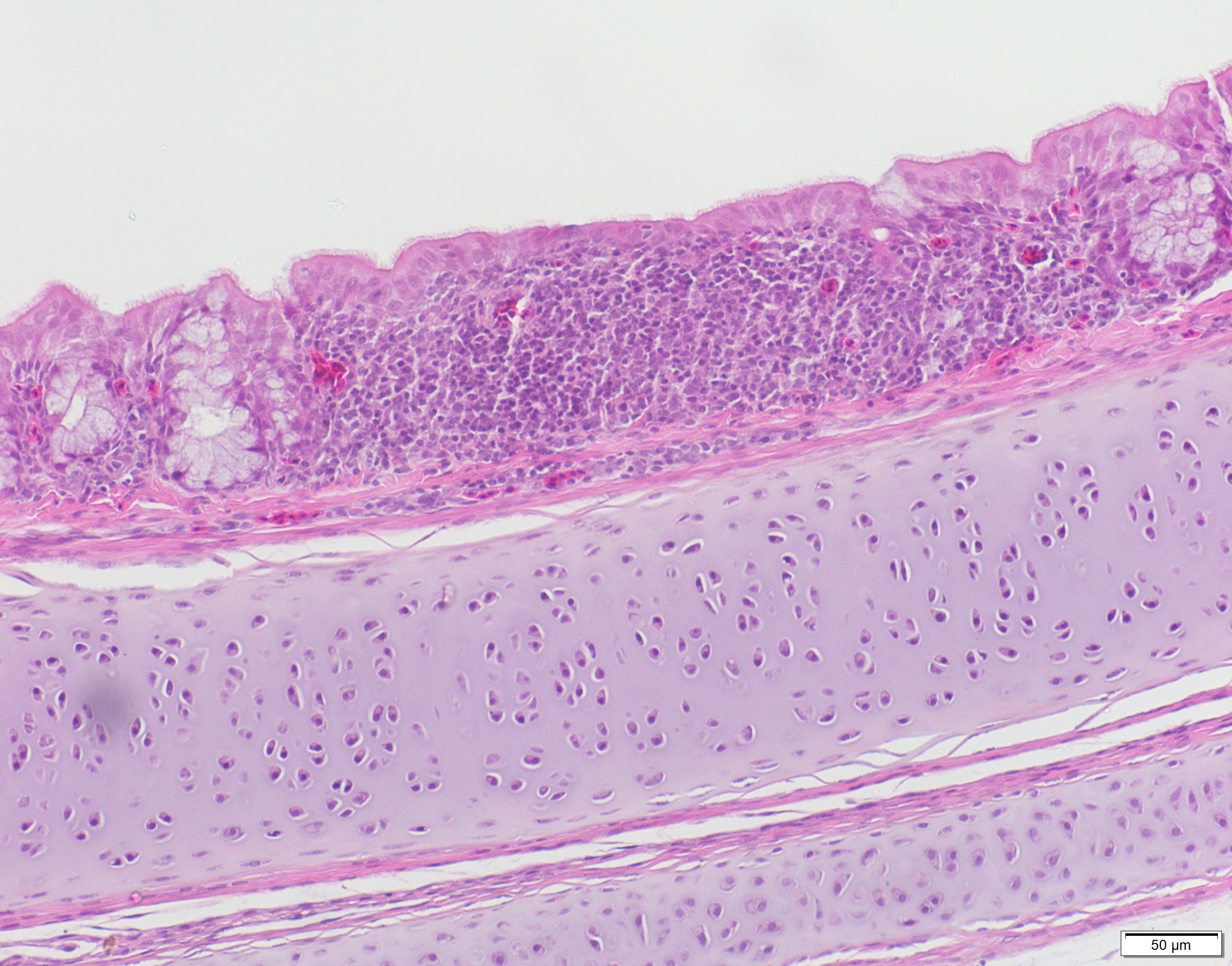

Supplement: Supplementary file 5 [file Data_Sheet_5.ZIP › Histopathological changes of trachea2/groupIII 5dpi.jpg]

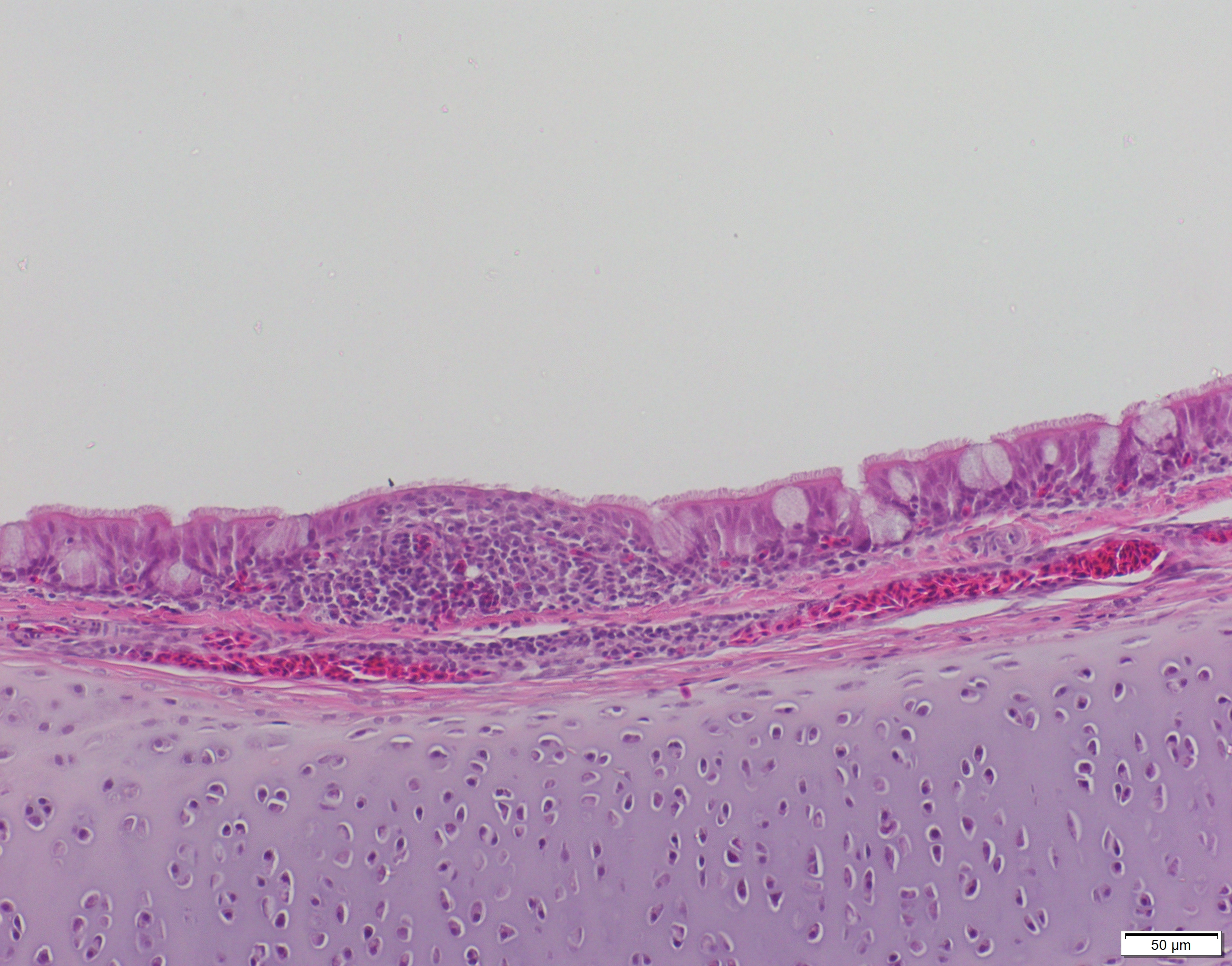

Supplement: Supplementary file 5 [file Data_Sheet_5.ZIP › Histopathological changes of trachea2/groupIII 7dpi.jpg]

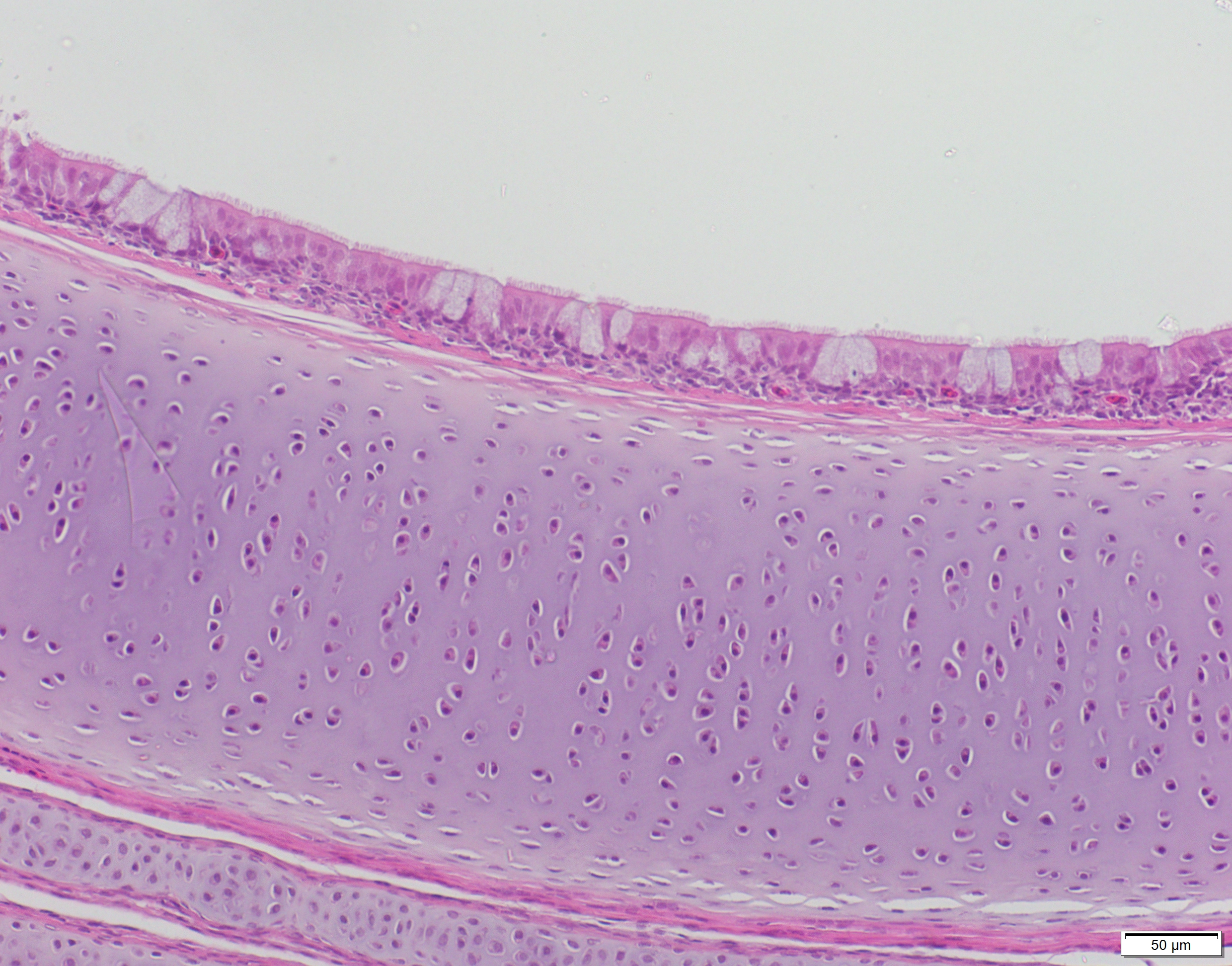

Supplement: Supplementary file 5 [file Data_Sheet_5.ZIP › Histopathological changes of trachea2/groupIV 1dpi.jpg]

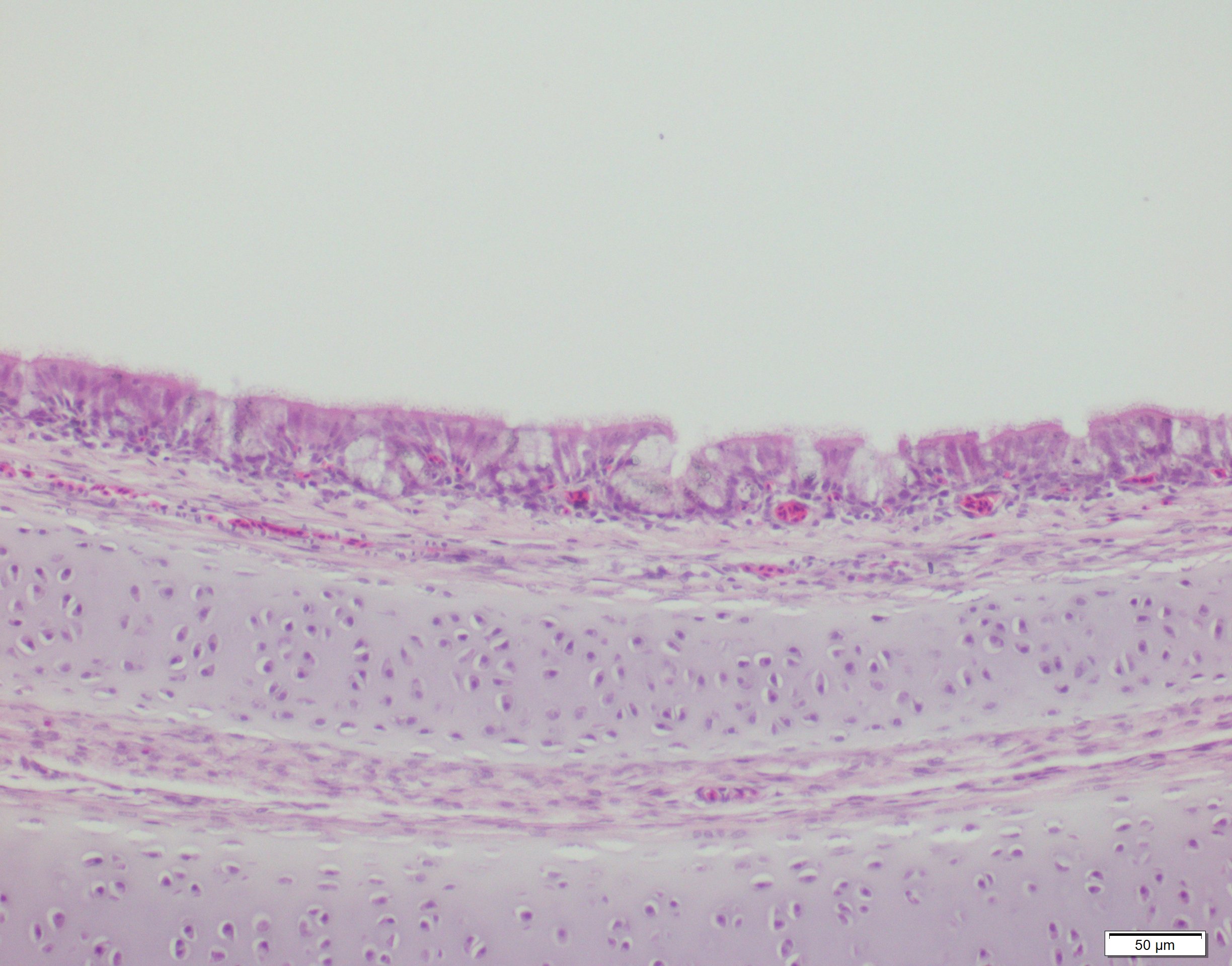

Supplement: Supplementary file 5 [file Data_Sheet_5.ZIP › Histopathological changes of trachea2/groupIV 3dpi.jpg]

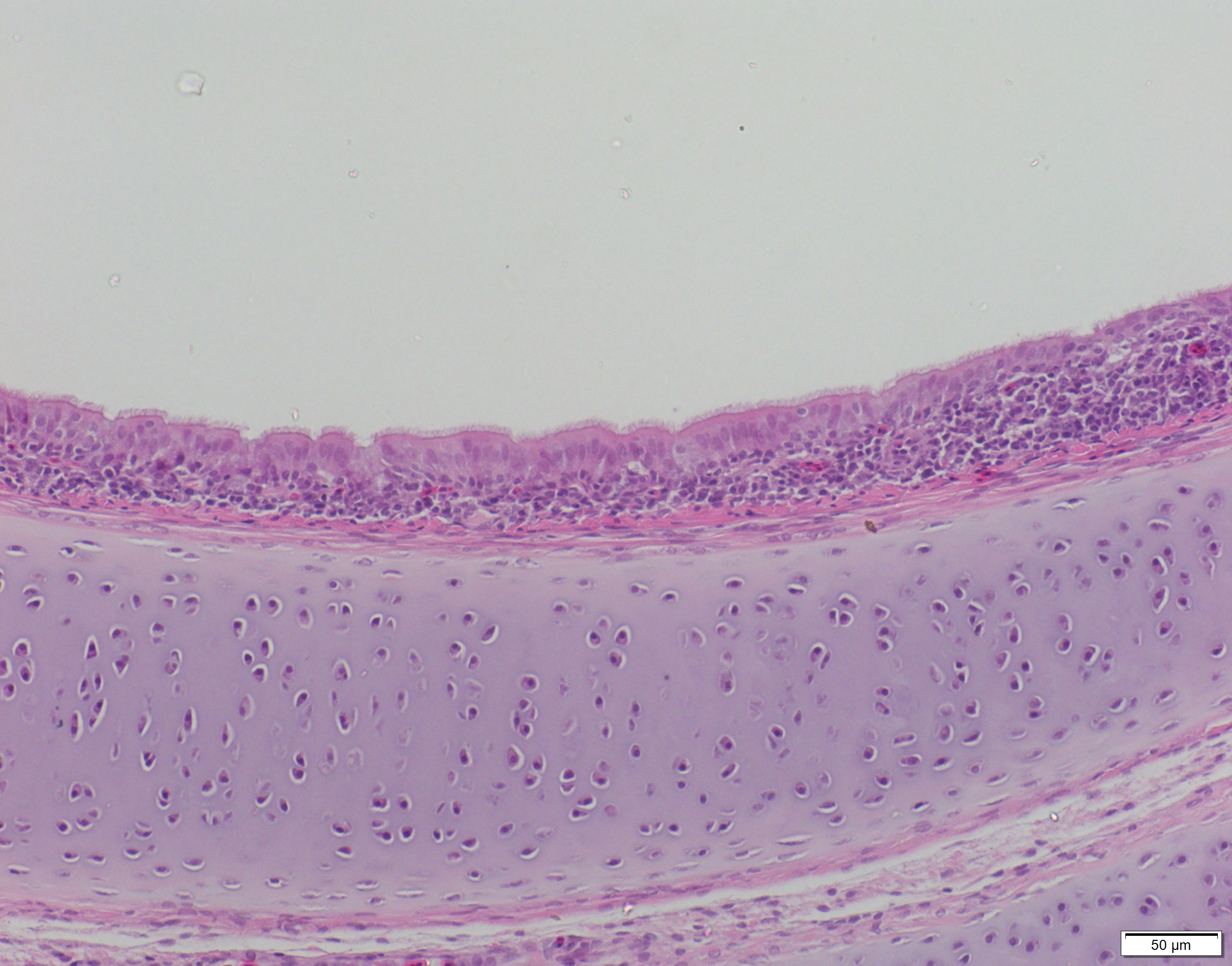

Supplement: Supplementary file 5 [file Data_Sheet_5.ZIP › Histopathological changes of trachea2/groupIV 5dpi.jpg]

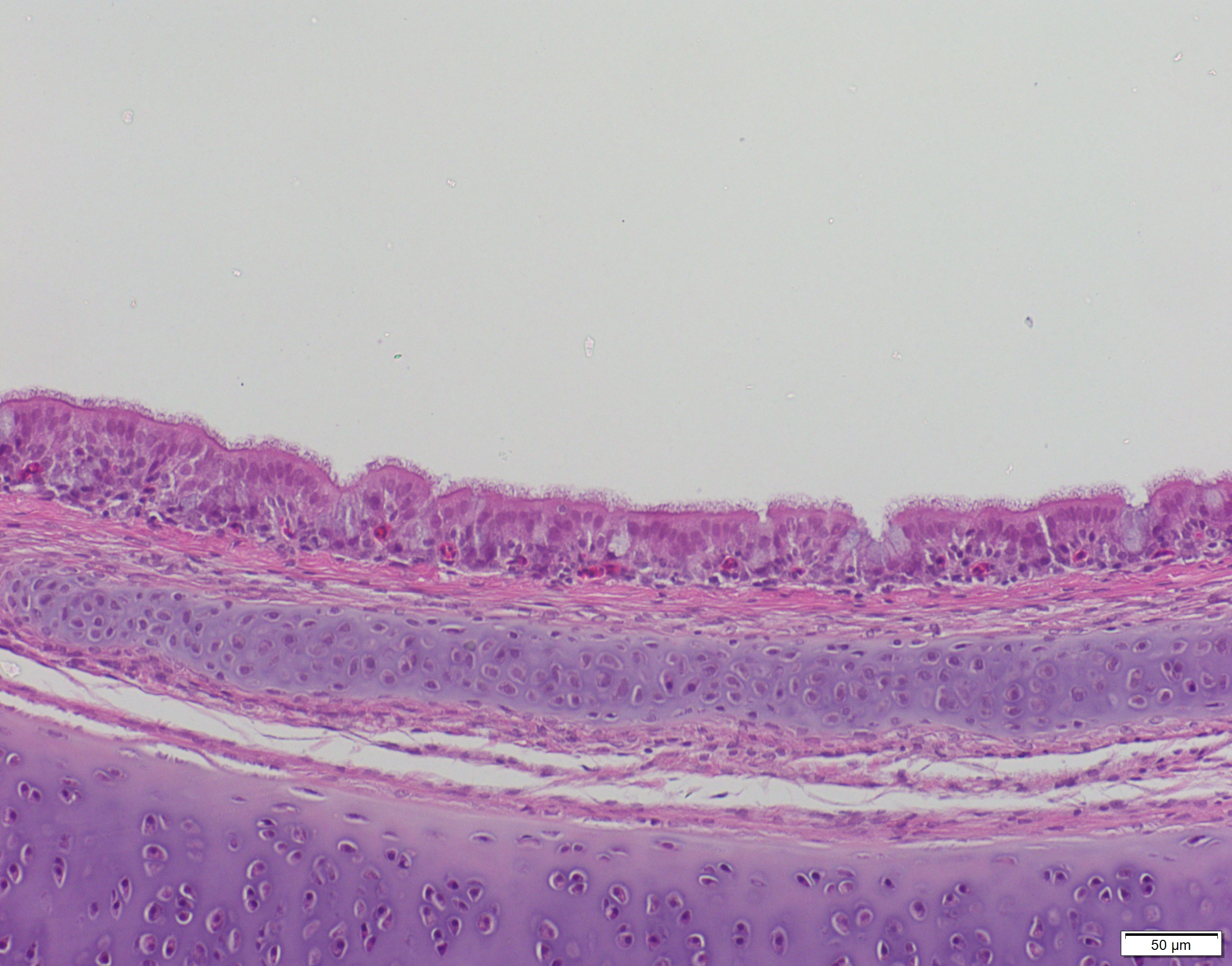

Supplement: Supplementary file 5 [file Data_Sheet_5.ZIP › Histopathological changes of trachea2/groupIV 7dpi.jpg]

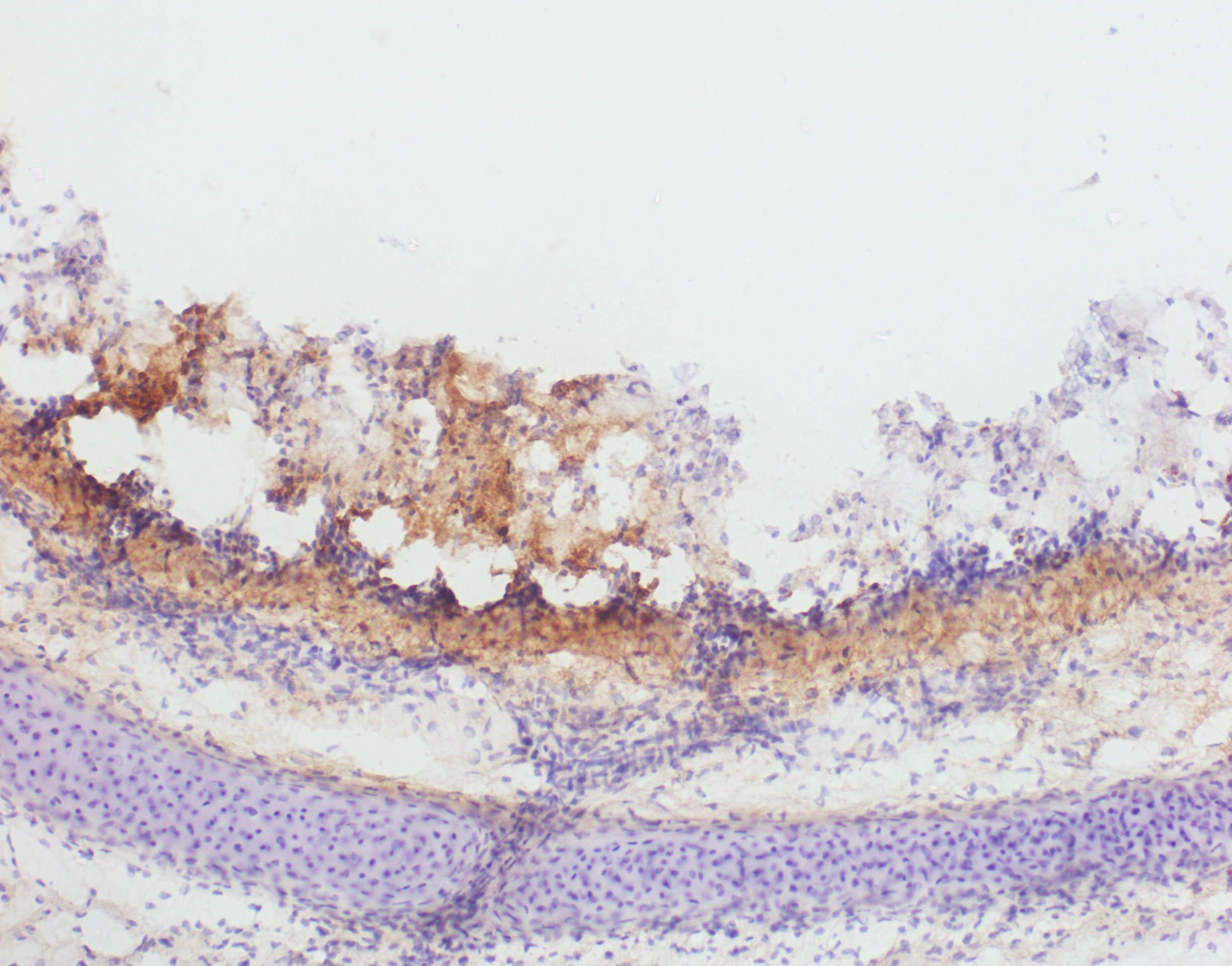

Supplement: Supplementary file 6 [file Data_Sheet_6.ZIP › Immunohistochemistry1/14doa groupI .tif]

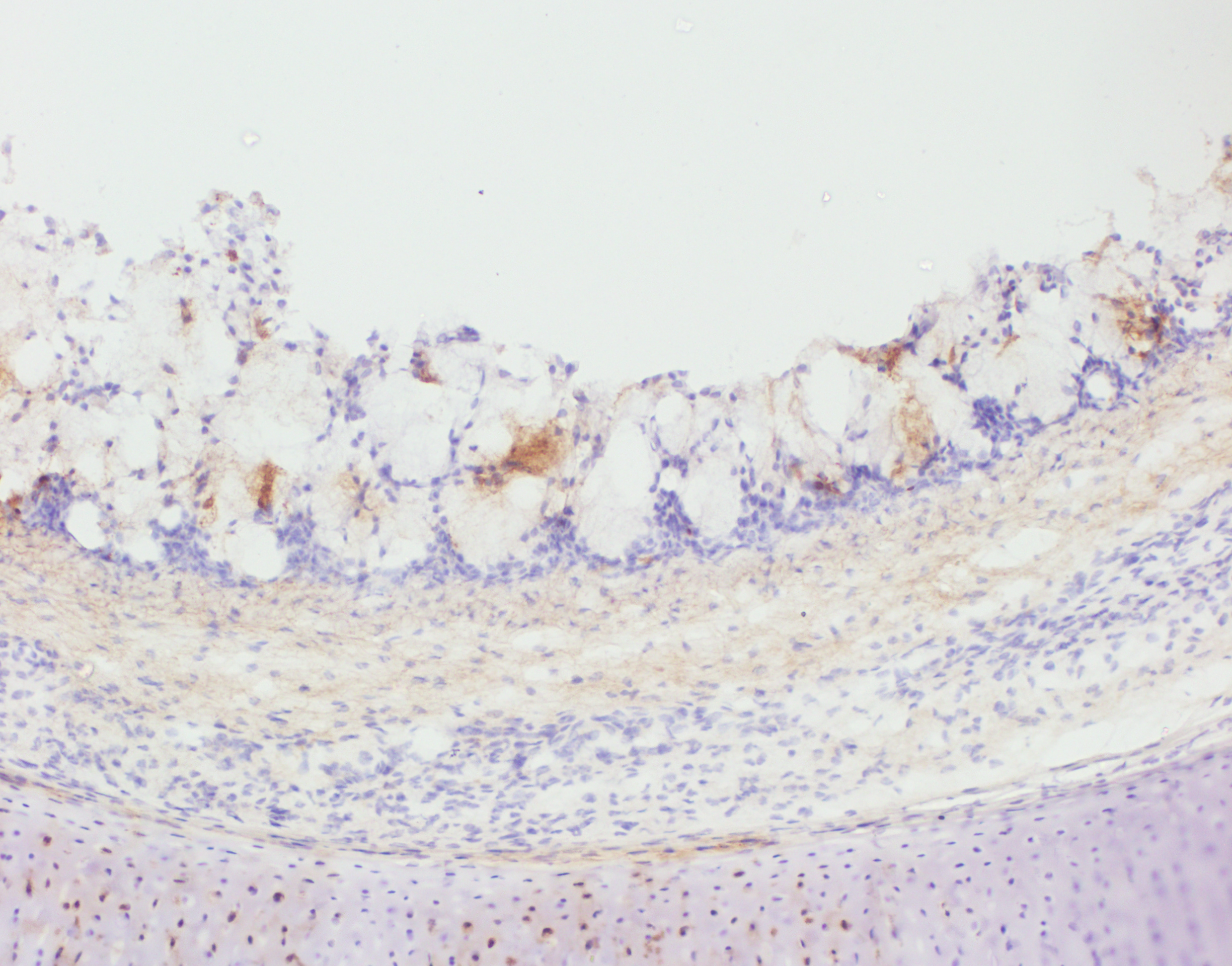

Supplement: Supplementary file 6 [file Data_Sheet_6.ZIP › Immunohistochemistry1/21doa groupI .tif]

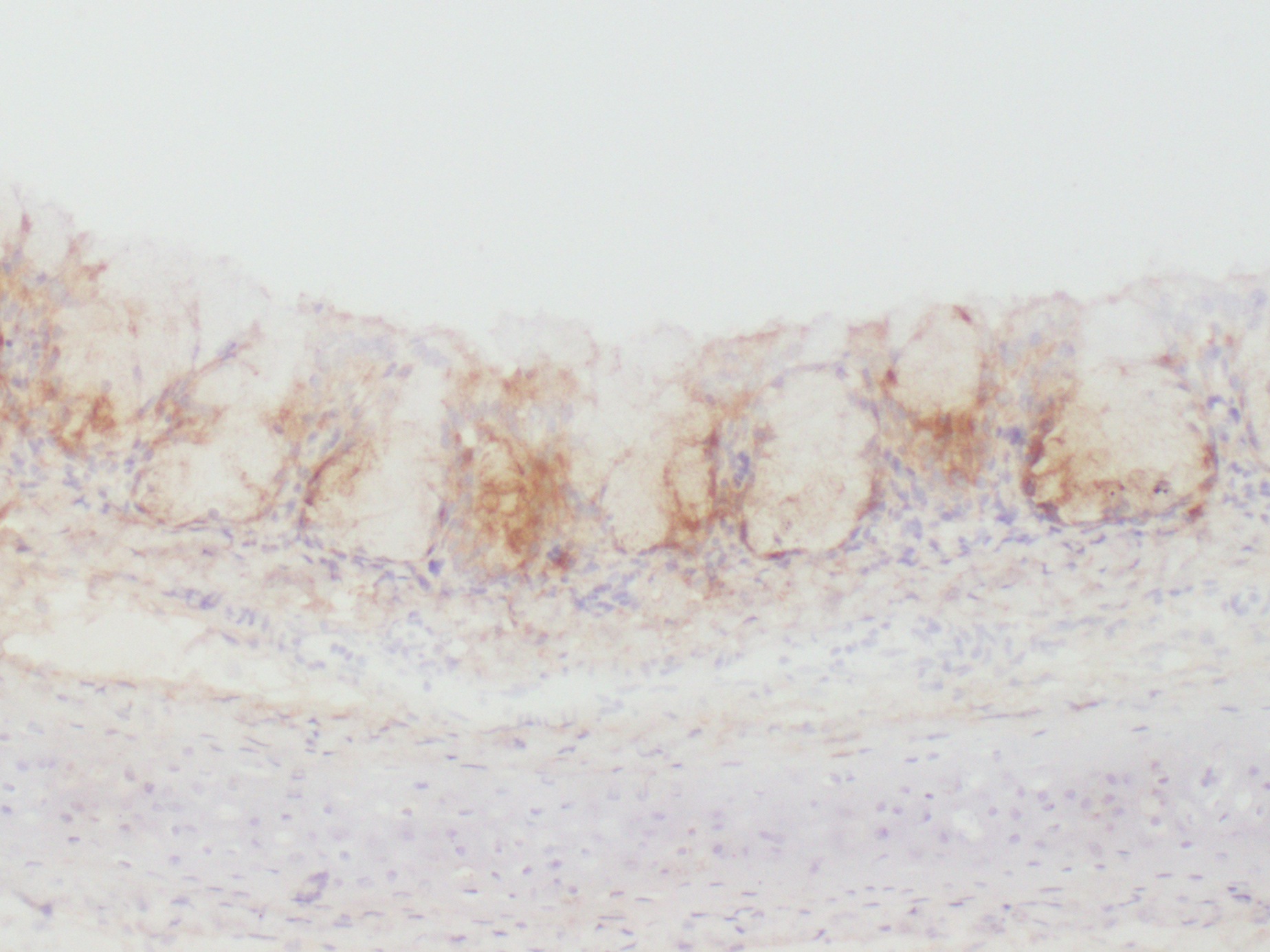

Supplement: Supplementary file 6 [file Data_Sheet_6.ZIP › Immunohistochemistry1/28doa groupI .tif]

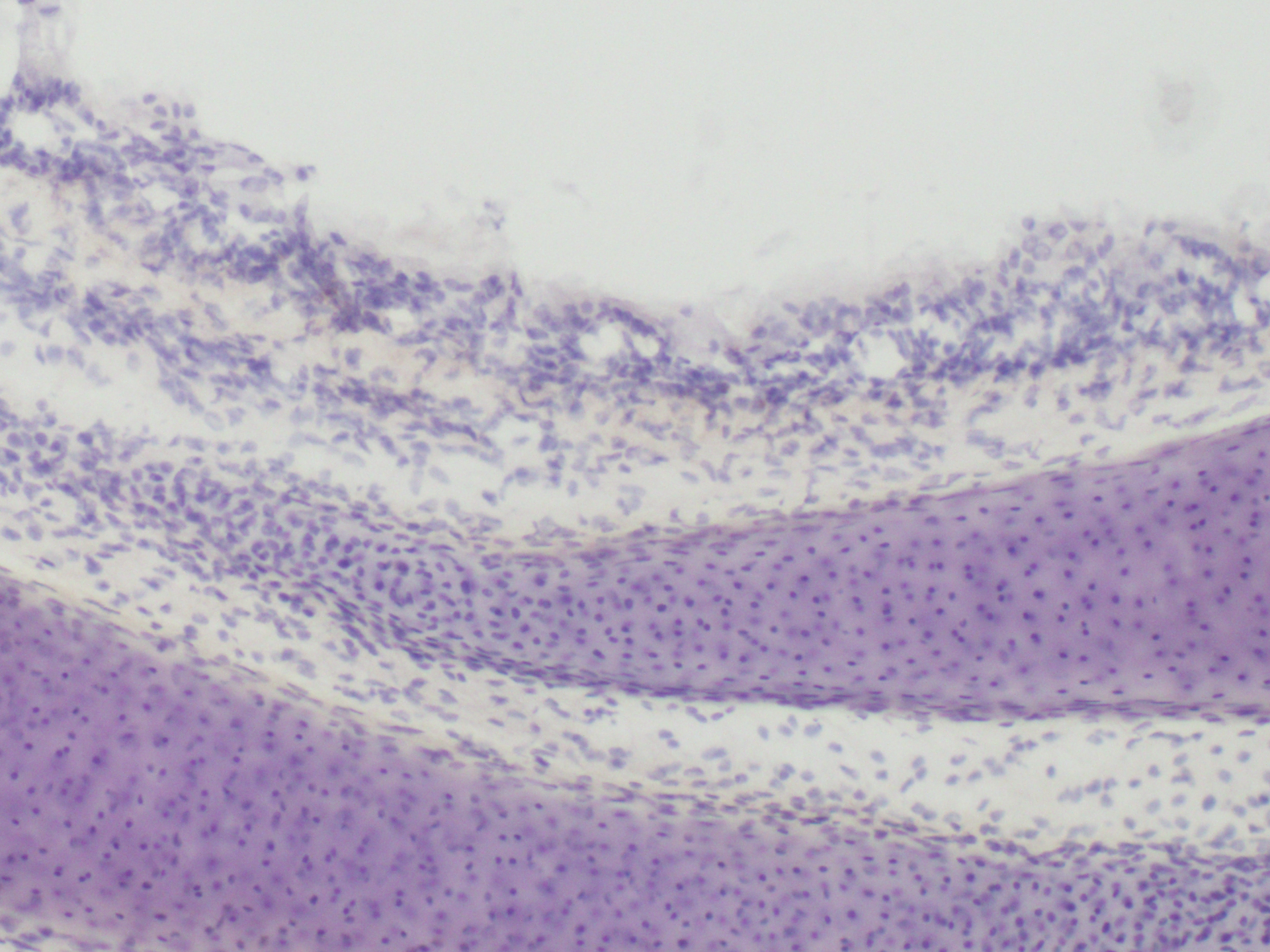

Supplement: Supplementary file 6 [file Data_Sheet_6.ZIP › Immunohistochemistry1/7doa groupI .tif]

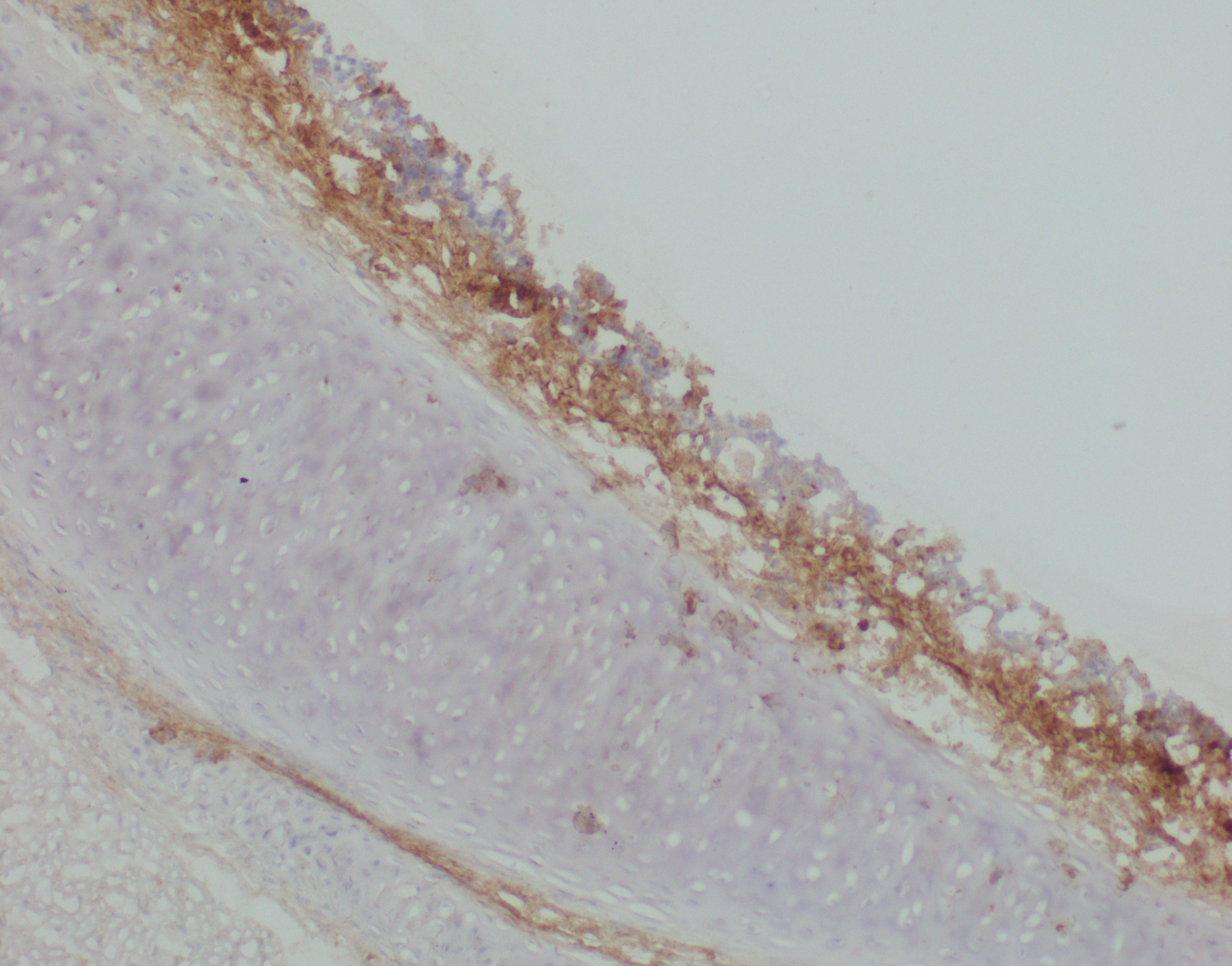

Supplement: Supplementary file 7 [file Data_Sheet_7.ZIP › Immunohistochemistry2/14doa group II.tif]

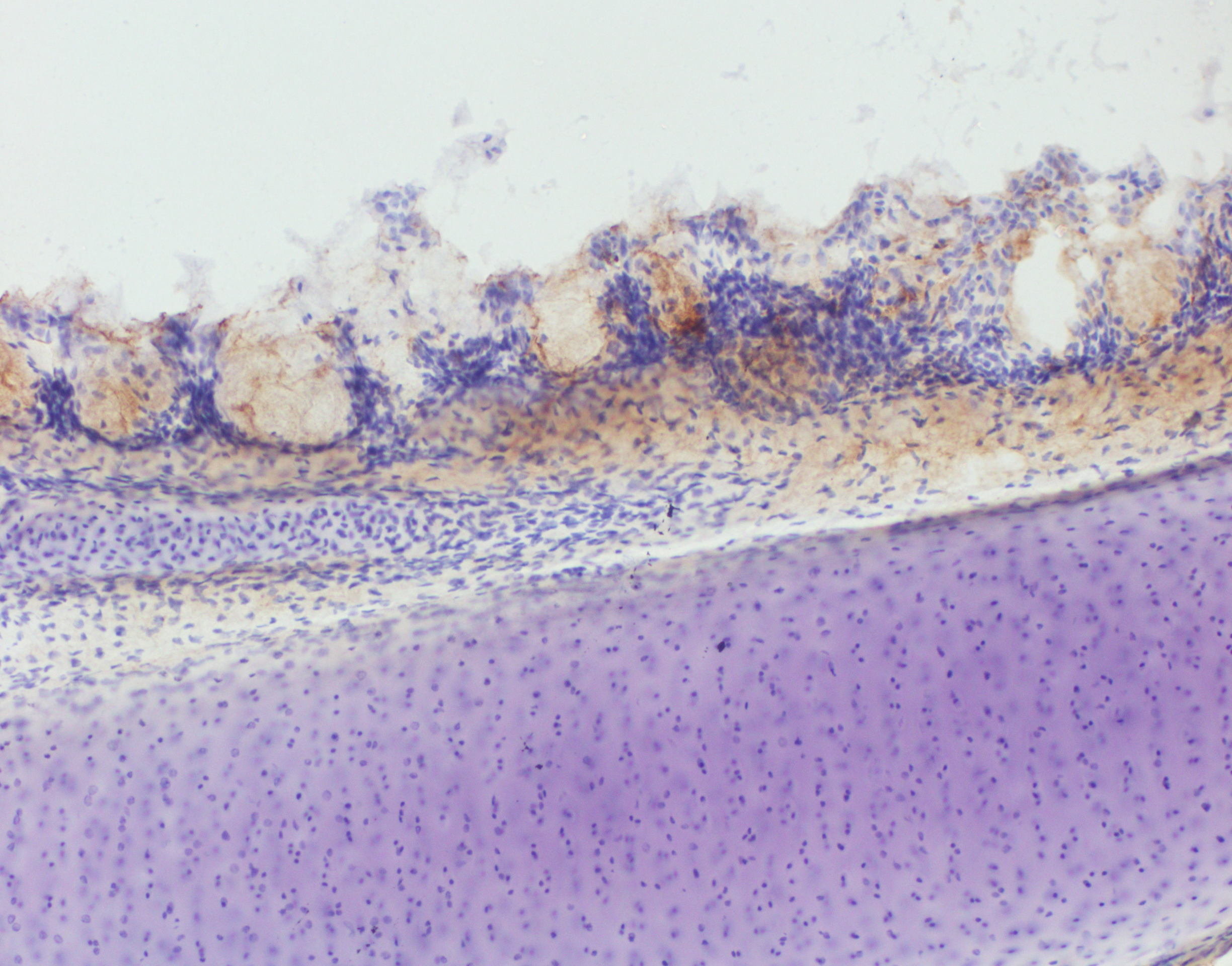

Supplement: Supplementary file 7 [file Data_Sheet_7.ZIP › Immunohistochemistry2/21doa group II.tif]

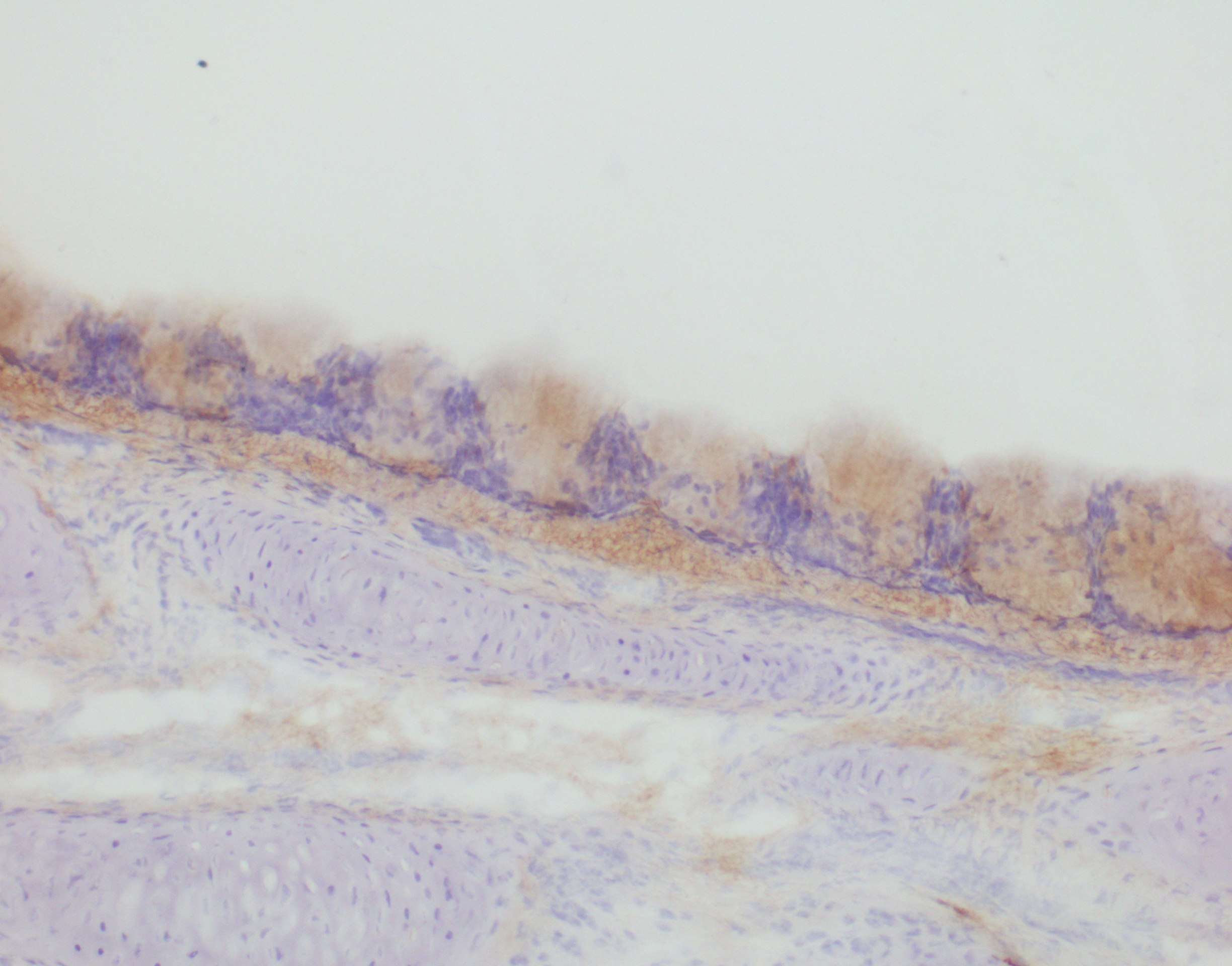

Supplement: Supplementary file 7 [file Data_Sheet_7.ZIP › Immunohistochemistry2/28doa group II.tif]

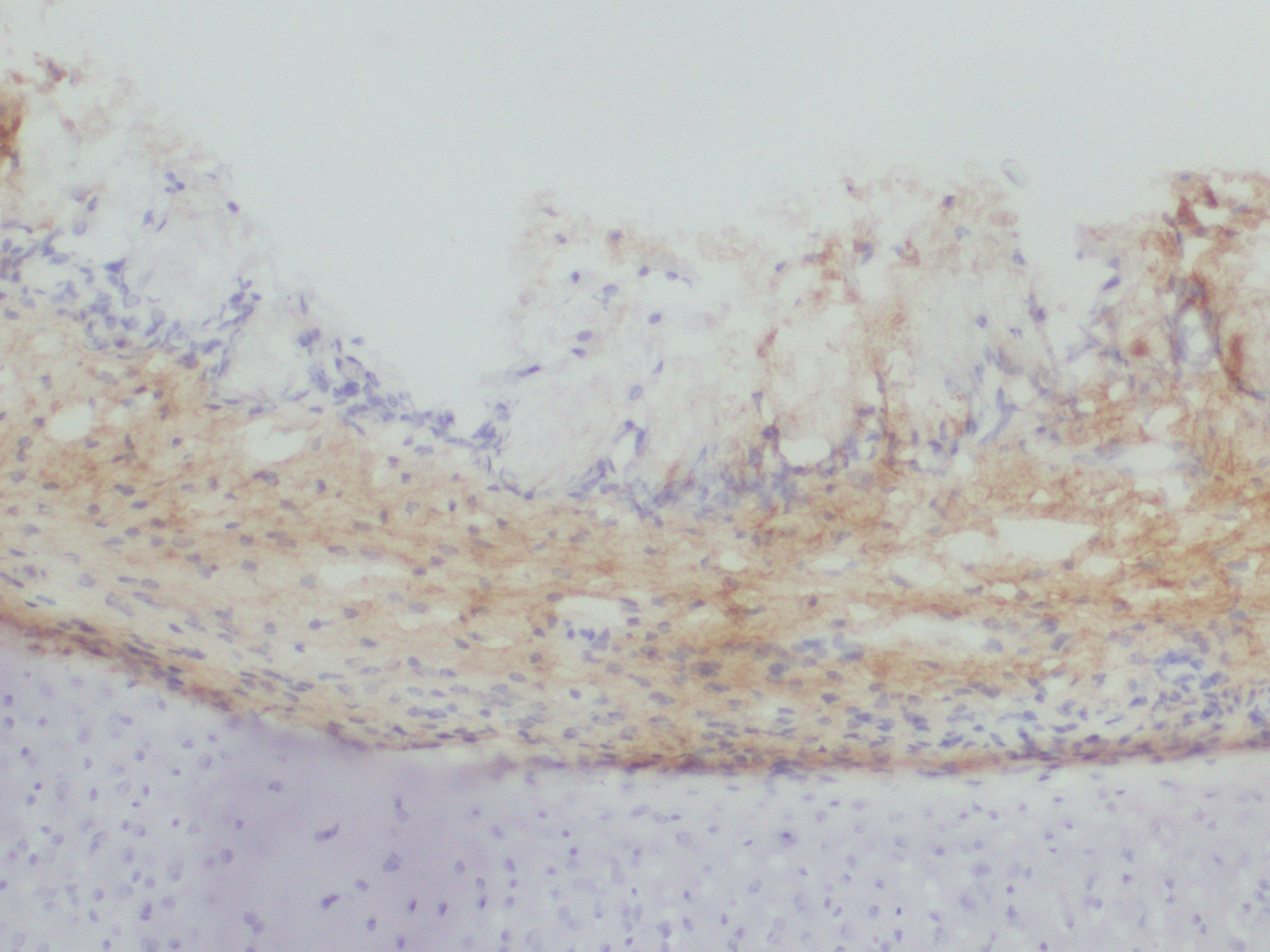

Supplement: Supplementary file 8 [file Data_Sheet_8.ZIP › Immunohistochemistry3/14doa groupIII.tif]

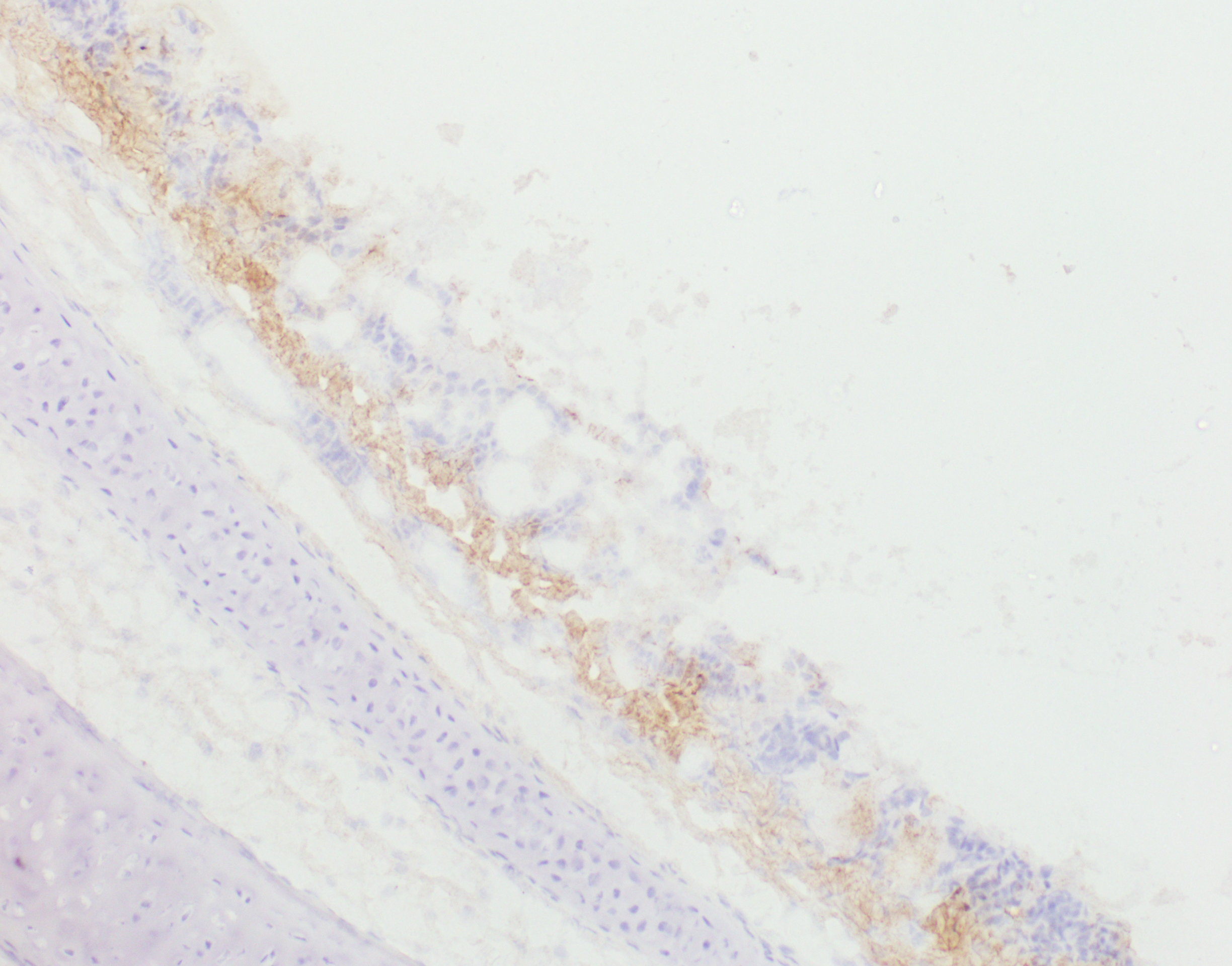

Supplement: Supplementary file 8 [file Data_Sheet_8.ZIP › Immunohistochemistry3/21doa group III.tif]

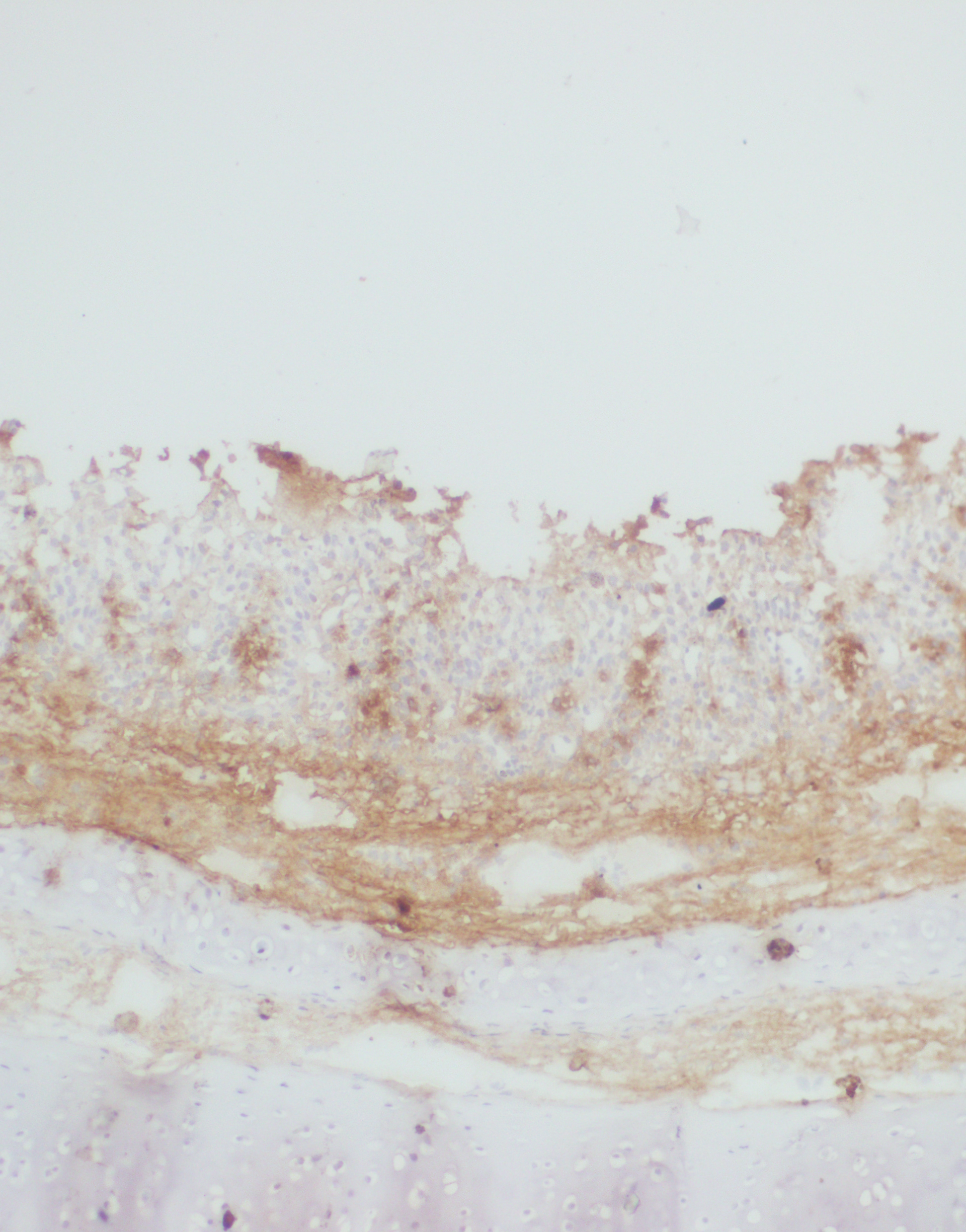

Supplement: Supplementary file 8 [file Data_Sheet_8.ZIP › Immunohistochemistry3/28doa group III.tif]

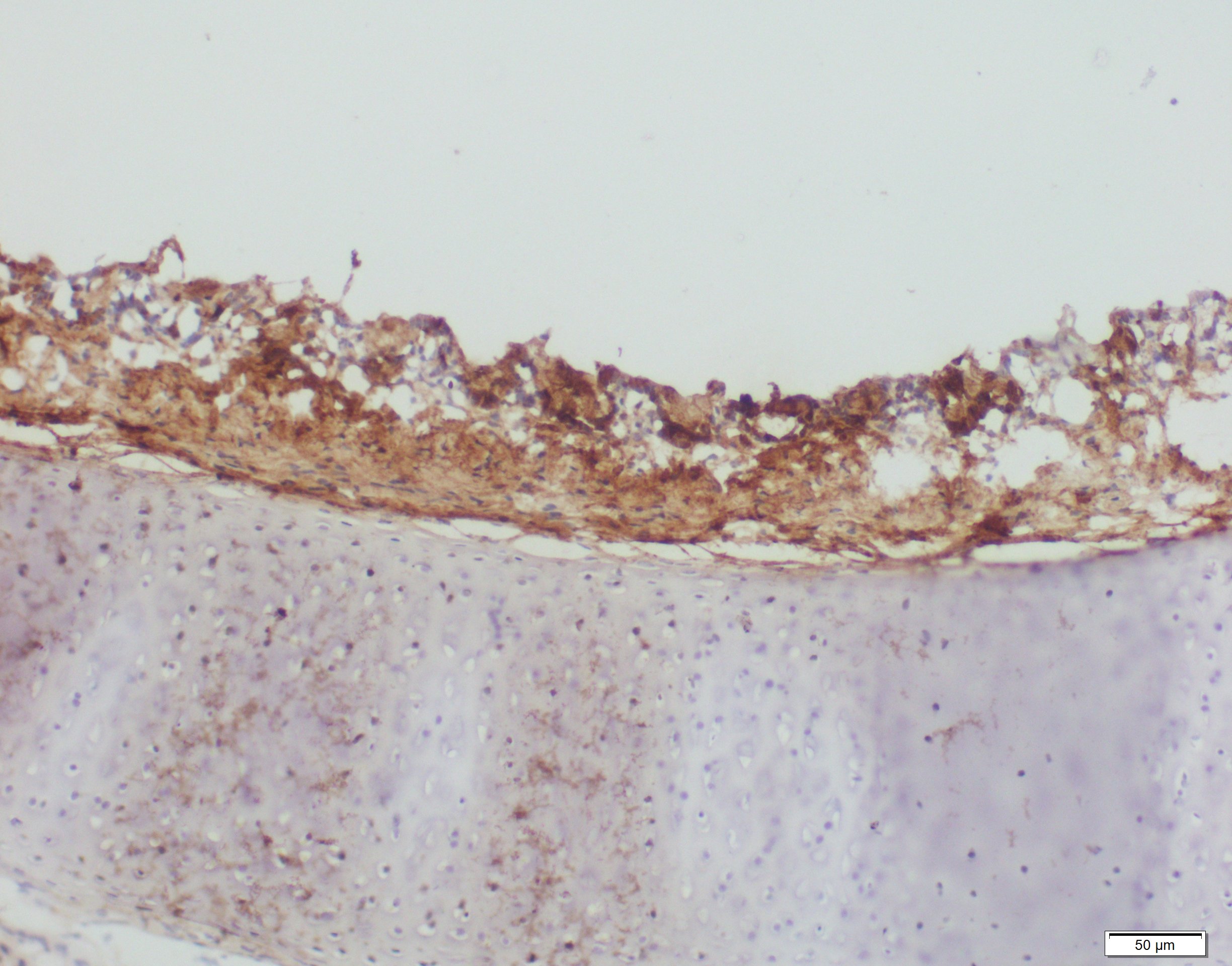

Supplement: Supplementary file 9 [file Data_Sheet_9.ZIP › Immunohistochemistry4/14doa group IV.jpg]

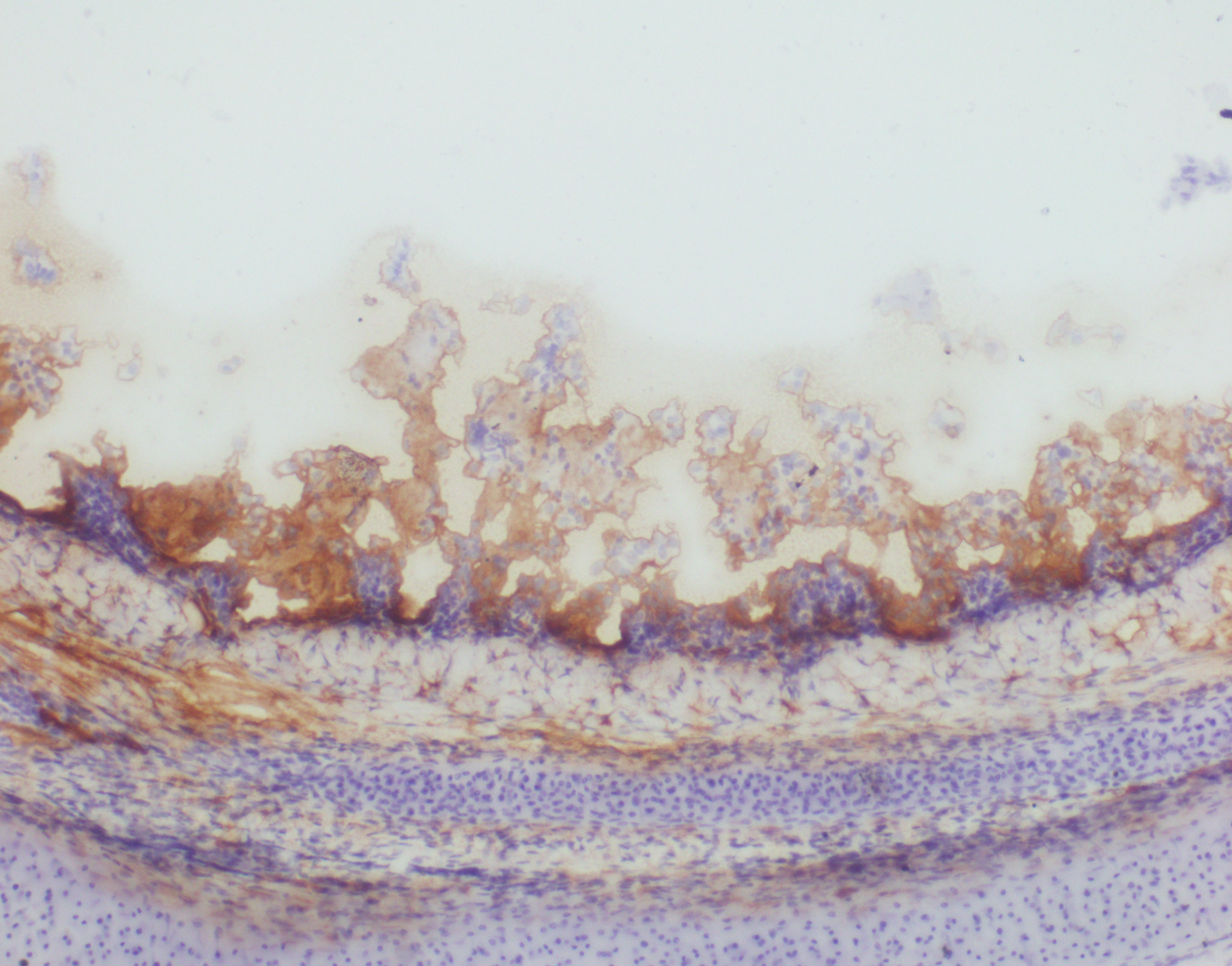

Supplement: Supplementary file 9 [file Data_Sheet_9.ZIP › Immunohistochemistry4/21doa group IV.tif]

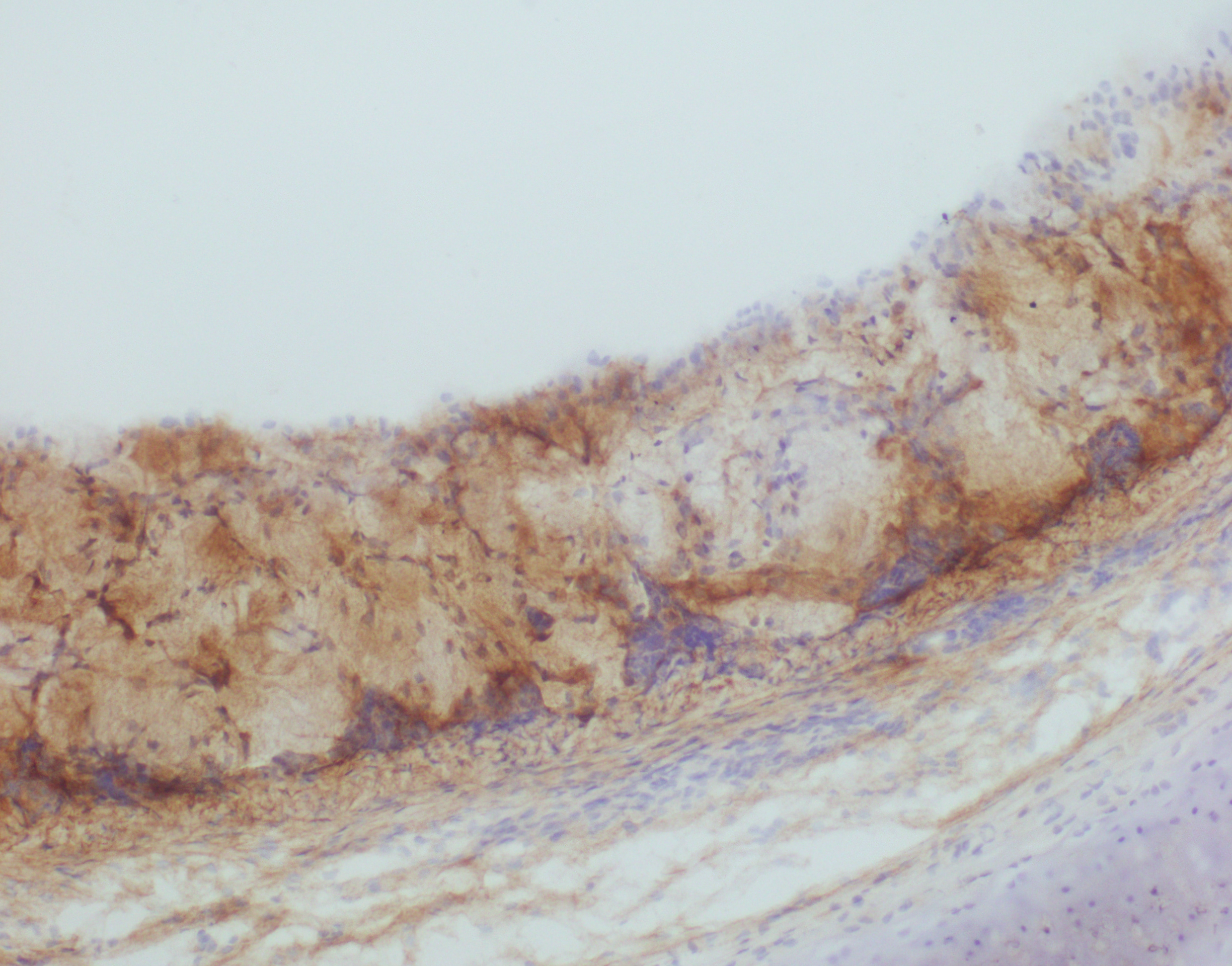

Supplement: Supplementary file 9 [file Data_Sheet_9.ZIP › Immunohistochemistry4/28doa group IV.tif]
