# Supplementary material for: Combined Effect of Shegandilong Granule and Doxycycline on Immune Responses and Protection Against Avian Infectious Bronchitis Virus in Broilers
Source: Front Vet Sci. 2021 Dec 20;8:756629. doi: 10.3389/fvets.2021.756629 (PMC8721878; doi:10.3389/fvets.2021.756629)
Supplement: Supplementary file 10 [file Data_Sheet_10.ZIP › additional files1/supplementary material 1.docx]

**Table1.** **Components of the herbal granule used in this study**

| Herb name | | Role in herbal power | Standard  dose (g) |
| --- | --- | --- | --- |
| Chinese name | Latin name |  |  |
| She Gan | *Belamcanda chinensis (L.)Redouté(radix)* | Monarch drug**^a^** | 250 |
| Di Long | *Pheretima* | Minister drug**^b^** | 200 |
| Bei Dou Gen | *Menispermum dauricum DC (radix)* | Minister drug | 150 |
| Wu Wei Zi | *Schisandra chinensis* | Adjuvant drug**^c^** | 100 |
| Wu Mei | *DarkPlumFruit* | Adjuvant drug | 100 |
| Jie Geng | *Platycodon grandiflorus (Jacq.) A. DC* | Adjuvant drug | 100 |
| Gan Cao | *Glycyrrhiza uralensis Fisch(radix)* | Guide drug**^d^** | 100 |

A traditional herbal formula generally contains different quantities of several herbs with different roles (Monarch, Minister, Adjuvant and Guide). In this herbal granule, the large dose of *Belamcanda chinensis* (*L*.) *Redouté* (*radix*) can clear heat and detoxification, remove phlegm and promote pharynx. *Pheretima* (*radix*) and *Menispermum dauricum DC* (*radix*) as minister drug, which have the function of antipyretic, diuretic, relaxing bronchial trachea, reducing pharyngeal swelling. As an adjuvant drug, *Schisandra chinensis* (*radix*), *Platycodon grandiflorus* and *DarkPlum- Fruit* (*radix*) have the effect of removing blood stasis and cooling blood, astringing lung to stop cough. *Glycyrrhiza uralensis Fisch* (*radix*), a tonifying herbal medicine, harmonizes the herbs and minimizes any potential side effects. All of these herbs work together exhibiting a synergistic action of relieving asthma, relieving phlegm and relieving cough, nourishing yin and clearing heat, collecting astringency and other functions, which are used to treat the symptoms of phlegm-heat stagnation and phlegm ringing in IB.

**^a^** The Monarch drug is the key component of an herbal formula, and it provides the principal therapeutic effects against a related disease.

**^b^** The Minister drug is the synergistic secondary element of an herbal formula. It potentiates the effects of the Monarch drug or treats the accompanying symptoms.

**^c^** The Adjuvant drug is generally intended to enhance the therapeutic effects and mitigate any adverse effects of the Monarch drug and/or Minister drug.

**^d^** The Guide drug is generally used to harmonize the other herbs; it may also be used to facilitate the uptake of other herbs by a specific organ or target tissue.
