# Supplementary material for: Combined Effect of Shegandilong Granule and Doxycycline on Immune Responses and Protection Against Avian Infectious Bronchitis Virus in Broilers
Source: Front Vet Sci. 2021 Dec 20;8:756629. doi: 10.3389/fvets.2021.756629 (PMC8721878; doi:10.3389/fvets.2021.756629)
Supplement: Supplementary file 10 [file Data_Sheet_10.ZIP › additional files1/supplementary material 2.docx]

**Table2.** The primer sequences for qRT-PCR

| **Gene** | **Primer sequence**  **（5’-3’）** | | **Accession**  **number** | | **Annealing temperature**  **(ºC)** | **Product size (bp)** | |
| --- | --- | --- | --- | --- | --- | --- | --- |
| IBV N | | F:GACGGAGGACCTGATGGTAA  R: CCCTTCTTCTGCTGATCCTG | | MK937830.1 | 58.5 | | 206 |
| IL-6 | | F:GTTCGCCTTTCAGACCTACCTG  R:ATCGGGATTTATCACCATCTGC | | NM_20468.1 | 60.9 | | 130 |
| IL-1β | | F: CCTTCGACATCTTCGACATCAA  R: AATGTTGAGCCTCACTTTCTGG | | NM_204524.1 | 58.5 | | 113 |
| TNF-α | | F: TGCTGTTCTATGACCGCC  R:CTTTCAGAGCATCAACGCA | | AY765397 | 57.0 | | 219 |
| IFN-γ | | F: AGCCGCACATCAAACACATA  R: AAGTCGTTCATCGGGAGCTT | | NM_205149.1 | 58.1 | | 115 |
| β-actin | | F:CCCAAAGCCAACAGAGAGAA  R: CCATCACCAGAGTCCATCAC | | NM_205518 | 57.7 | | 140 |

Abbreviation: **qRT-PCR**, quantitative real-time polymerase chain reaction; IL-6, interleukin-6; IL-1β, interleukin-1β; TNF-α, tumor necrosis factor-alpha; IFN-γ ,  interferon gamma. F, Forward primer; R, Reverse primer.
